# Olefination of Alcohols and Alkyl Halides via Oxidative Alkyl Electrophile–Olefin Metathesis

**DOI:** 10.1021/acs.orglett.6c01890

**Published:** 2026-06-26

**Authors:** Jason Wu, Elise Meng, Molly E. Jones, Tristan H. Lambert

**Affiliations:** Department of Chemistry and Chemical Biology, 5922 Cornell University, Ithaca, New York 14853, United States

## Abstract

A method for the olefination of benzylic halides and alcohols with unactivated alkenes via oxidative alkyl electrophile-olefin metathesis (AEOM) is described. The procedure employs a simple bicyclic diazene to promote the formation of a new carbon–carbon double bond between an alcohol or alkyl halide and an olefin through a sequence of [3 + 2] cycloaddition and cycloreversion steps. The synthesis of substituted styrenes and cinnamate esters is demonstrated.

## Graphical abstract

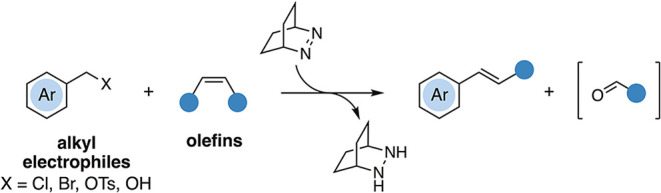

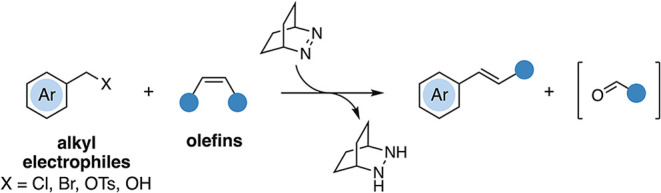

Olefins are among the most fundamental structural motifs in organic chemistry, and methods for their synthesis occupy a central role in both academic and industrial settings.^1,2^ Classical approaches to olefin formation, including elimination reaction,^3^ the Wittig reaction^4^ and related olefinations,^5^ and transition metal-catalyzed cross-coupling processes,^6^ provide powerful and complementary strategies for alkene construction. A more modern approach involves metathesis reactions^7^ that enable the direct exchange of functional group fragments, often allowing rapid access to complex products from simple precursors. While substantial advances have been made in olefin metathesis^7^ and carbonyl–olefin metathesis,^8,9^ the extension of metathesis concepts to other abundant functional groups remains comparatively underdeveloped.

In this context, we have been interested in the use of hydrazonium intermediates as a platform for bond reorganization. Our prior studies in carbonyl–olefin metathesis (COM) established that hydrazonium ions, generated from condensation of hydrazines with carbonyl compounds, can engage olefins in reversible [3 + 2]-cycloaddition/cycloreversion sequences to effect productive fragment exchange.^10^ Building on this mechanistic framework, we recently demonstrated that analogous hydrazonium intermediates generated by the alkylation of diazenes can enable a metathetical exchange between alkyl electrophiles and olefins (Figure 1A).^11^ Because this reaction generated an aldehyde coproduct rather than a new alkyl halide, we termed this process oxidative alkyl halide–olefin metathesis. In that system, intramolecular cyclization of alkenyl alkyl halides such as **1** furnished polycyclic olefin products **2**, thereby establishing that alkyl electrophiles can participate in metathesis-like reactivity. However, this transformation was limited to intramolecular processes and did not provide a general strategy for intermolecular fragment exchange or olefin synthesis.

**Figure 1:**
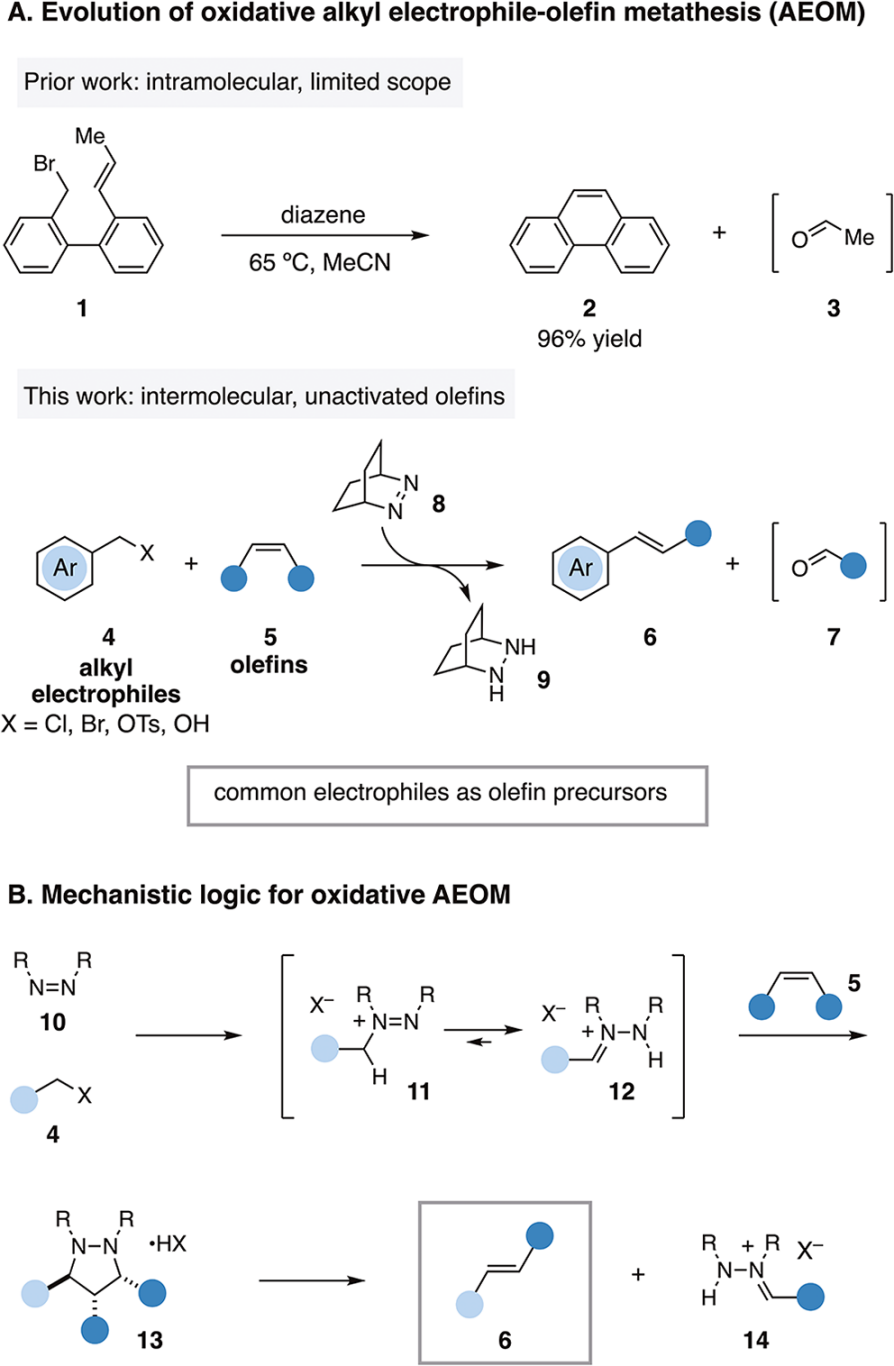
Oxidative alkyl electrophile-olefin metathesis.

We questioned whether this reactivity could be extended beyond cyclization manifolds to enable intermolecular exchange between alkyl electrophiles **4** and olefins **5**. Such a transformation would constitute a cross alkyl electrophile–olefin metathesis (AEOM), in which common functional groups such as alcohols and alkyl halides could serve as precursors to alkenes. Conceptually, this approach would provide a complementary disconnection to existing olefination strategies by enabling direct conversion of electrophiles into olefins under nonbasic conditions. Achieving this outcome is challenging, however, as intermolecular exchange must outcompete substitution, elimination, and hydrolysis while surmounting the barriers to cycloaddition and cycloreversion and avoiding diazene denitrogenation. Here, we report that these challenges can be overcome, and that diazene-mediated reactivity can be extended to a more general intermolecular regime.

The general blueprint for this AEOM idea is shown in Figure 1B. Alkylation of a diazene **10** by an alkyl electrophile **4** forms diazenium ion **11**, which can undergo tautomerization to form the corresponding hydrazonium **12**. Cycloaddition of **12** with an olefin **5** affords a pyrazolidine cycloadduct intermediate **13**. Cycloreversion of this intermediate furnishes metathesized olefin **6** and hydrazonium **14**. The latter, when hydrolyzed, furnishes an oxidized, metathesized alkyl fragment in the form of an aldehyde and the reduced hydrazine (**7** and **9**).

The identification of optimized procedures for AEOM was straightforward (see SI for detailed optimization studies). In the case of benzyl bromide **15** (Figure 2A), reaction with 3 equiv *cis*-4-octene (**16**) and 1 equiv diazene **8** at 120 °C in CHCl_3_ in a capped vial led to styrene **17** in 84% yield exclusively as the (*E*)-isomer. We believe that this selectivity results from a highly diastereoselective cycloaddition step (see **13** in Figure 1). The same conditions, with the addition of 2 equiv TFA and 4 Å molecular sieves, could be employed for reaction of alcohol **18** (Figure 2B), which furnished **17** in 75% yield as a single stereoisomer.

**Figure 2:**
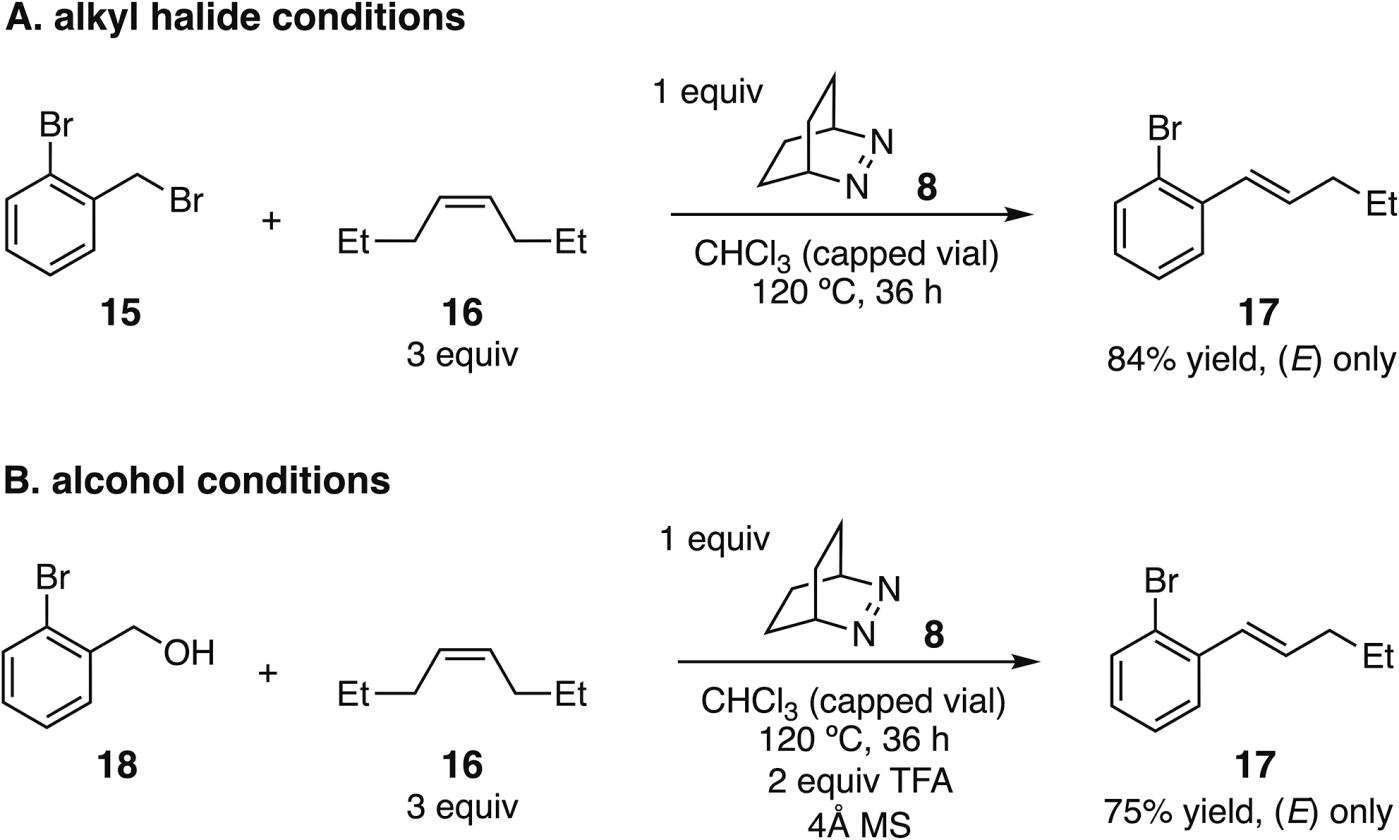
Optimized conditions for oxidative alkyl electrophile-olefin metathesis with benzyl bromide **15** and benzyl alcohol **18**.

Using these optimized conditions, we examined the scope of benzylic electrophiles across several classes of olefin partners (Figure 3).^12^ For reaction with *cis*-4-octene, benzyl bromide, chloride, or p-toluenesulfonate all afforded styrene **19** in good yields. Similarly, substituted styrenyl products **17**, **20**-**22** were produced with good efficiency from either bromide or chloride starting materials. Notably, pentafluorobenzyl bromide was the most reactive substrate examined and delivered the corresponding product **23** in near-quantitative yield.

**Figure 3:**
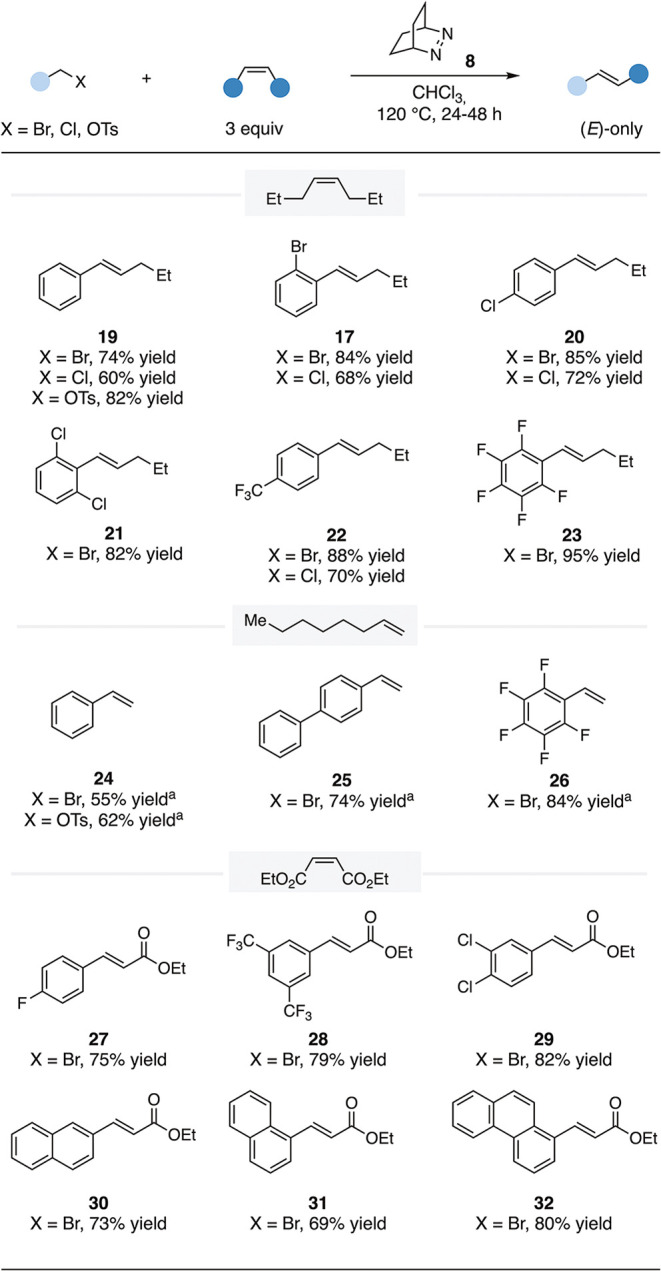
Alkyl bromide and olefin scope studies for cross alkyl halide-olefin metathesis. See SI for detailed procedures. Yields determined on purified products. ^a^Yields determined by ^1^H NMR with mesitylene as internal standard.

Methylenation of electrophiles could also be achieved with the use of a terminal olefin like 1-octene. In this case, benzyl bromide or benzyl p-toluenesulfonate led to the formation of styrene (**24**) in good yields, while styrenes **25** and **26** could be accessed in somewhat higher efficiencies. We believe that the exclusive transfer of the less-substituted carbon clearly reflects a strong regioselection in the cycloaddition step of the metathesis mechanism.

In addition to unactivated olefin reactants, we found that diethyl maleate could be employed, leading to the formation of ethyl cinnamates. In this case, products **27**-**32** were generated in good yields, again with complete stereoselectivity for the *E*-isomers.

We next investigated the scope of AEOM with benzyl alcohols (Figure 4). Using *cis*-4-octene as the olefin reactant, a variety of ortho- and para-substituted products (**19**, **20**, **23**, **33**-**40**) could be generated efficiently ranging from electron-deficient (**23**, **34**-**36**) to electron-rich (**33**, **37**-**38**). Notably, products **39** and **40** possess potentially base-sensitive phenolic ester groups, which are well tolerated under the acidic conditions of this procedure. In addition, we found that the conditions for alcohol AEOM were compatible with the maleate reactant, leading to cinnamates **41**-**48** in good to high yields.

**Figure 4:**
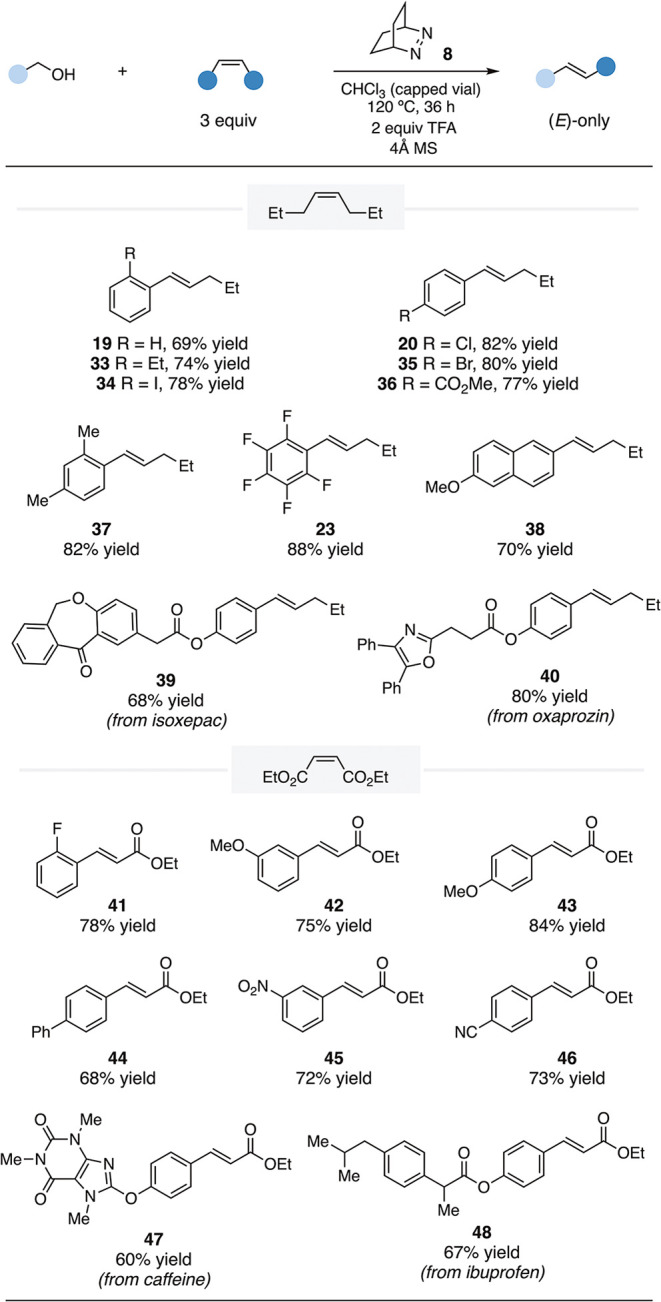
Alcohol and olefin scope studies for cross alcohol-olefin metathesis. See SI for detailed procedures. Yields determined on purified products.

This olefination procedure occurs under relatively mild conditions and is enabled by a simple diazene **8**. The synthesis of **8** is straightforward and can be easily performed on preparative scale (Figure 5).^13^ Combining commercially available 1,3-cyclohexadiene (**49**) and 4-phenyl-1,2,4-triazoline-3,5-dione (PTAD, **50**) at room temperature leads to cycloadduct **51** in nearly quantitative yield. Hydrogenation followed by hydrolytic decomposition of the urazole moiety in the presence of oxygen then furnishes the diazene **8** in high yield. We have regularly prepared up to 4 g of **8** in a single run. In terms of stability, **8** is known to be remarkably resistant to thermal denitrogenation,^14^ in contrast to other diazenes. As evidence of this stability, we found that heating **8** at 120 °C for 24 h in chloroform or toluene resulted in no observable loss of material as judged by ^1^H NMR using an internal standard (see SI for detailed thermal stability studies).

**Figure 5:**
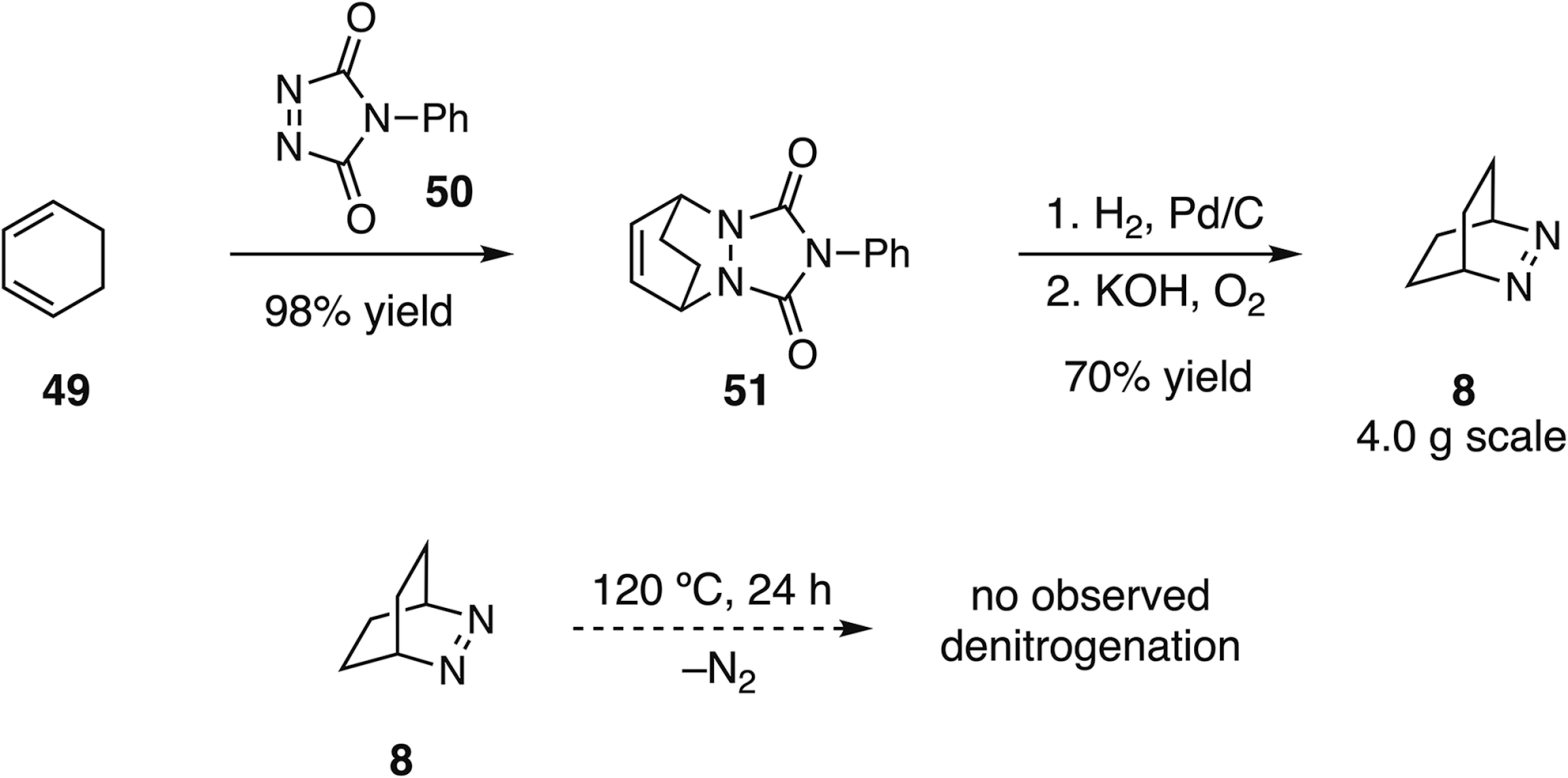
Synthesis and stability of diazene **8**.

In summary, we have demonstrated that the diazene-mediated oxidative AEOM between benzylic electrophiles and unstrained, *cis*-olefins is feasible. Although the present system employs stoichiometric diazene under the optimized conditions,^15^ it establishes the feasibility of intermolecular electrophile–olefin metathesis and defines a new reactivity manifold for hydrazonium-mediated bond exchange. Given the breadth of accessible electrophiles and the mild, nonbasic conditions employed, this approach provides a complementary entry to olefin synthesis and suggests opportunities for further development through catalyst design and expansion to additional substrate classes.

## Supplementary Material



## Data Availability

The data underlying this study are available in the published article and its Supporting Information.

## Notes

Experimental procedures and product characterization data (PDF)
