# Supplementary material for: Olefination of Alcohols and Alkyl Halides via Oxidative Alkyl Electrophile–Olefin Metathesis
Source: Org Lett. 2026 Jun 26;28(27):8554–8. doi: 10.1021/acs.orglett.6c01890 (PMC13366573; doi:10.1021/acs.orglett.6c01890)
Supplement: Supplementary file 1 [file ol6c01890_si_001.pdf]

Supporting Information

Olefination of Alcohols and Alkyl Halides via Oxidative Alkyl Electrophile-Olefin Metathesis

Jason Wu[†], Elise Meng[†], Molly E. Jones[†], and Tristan H. Lambert*[†]

[†] Department of Chemistry and Chemical Biology, Cornell University, Ithaca, New York 14853, USA

Email: Tristan.Lambert@Cornell.edu

Table of Contents:

1. GENERAL INFORMATION 2
2. OPTIMIZATIONS 4
3. DIAZENE SYNTHESIS 16
4. DIAZENE STABILITY 18
5. SYNTHESIS OF ALCOHOLS 19
6. PRODUCT SYNTHESIS AND CHARACTERIZATION 24
7. NMR SPECTRA 41
8. REFERENCES 105

1. GENERAL INFORMATION

General Methods:

Commercial reagents were purchased from Chemscene, Fisher Chemicals, Sigma-Aldrich, BeanTown Chemical Company, Oakwood Chemical Company, TCI, Acros Organics, Ambeed, Santa Cruz Biotechnology, Astatech and Combi-Blocks and used without purification unless otherwise noted. Specifically, compounds 15, 16, 18, 49, S17–S21, S23–S26, and S28–S31 are commercially available and were used as received without further purification.

All reactions were performed in a fume hood under atmospheric pressure unless otherwise noted. Vials and stir-bars used were purchased from Fisherbrand™: Vial 1 DRAM with Black Phenolic Screw Cap and Polyvinyl-faced pulp Liner and PTFE-silicone septa were purchased from JG Finneran: 13mm x 0.060" Red PTFE/Silicone Septa. All reactions requiring elevated temperatures were conducted using either a TRI-BLOCK™ reaction block (Chemglass) or an OPTICHEM® reaction block (Chemglass) maintained at the indicated temperature.

Reactions were monitored by thin-layer chromatography (TLC) on EMD Silica Gel 60 F254 plates under 254 nm UV light. Flash column chromatography (FCC) was performed using silica gel 60 (230-400 mesh) from SilicaFlash. Preparative TLC was performed using MilliporeSigma™ Supelco™ Preparative TLC Plates Silica Gel. Organic mixtures and solutions were concentrated under reduced pressure on a Büchi rotary evaporator R-200.

Proton nuclear magnetic resonance (1H NMR), fluorine nuclear magnetic resonance (19F NMR) and carbon nuclear magnetic resonance (13C NMR) data were recorded on Bruker Magnet System (500 MHz) at 25 °C unless otherwise noted. Spectra were analyzed using Mestrenova by Mestrelab Research. All chemical shifts are reported in parts per million (ppm) downfield from tetramethylsilane (TMS). Proton resonances are referenced to residual protium in the respective NMR solvent. Carbon resonances are referenced to carbon resonances of the respective NMR solvent. Representation of NMR data is as follows: chemical shift, multiplicity (s = singlet, d = doublet, t = triplet, q = quartet, p = pentet, sext = sextet, sept = septet, m = multiplet, br = broad), coupling constant (Hz), integration.

Mass spectral (MS) data are obtained on Advion Mass Spectrometer equipped with an Atmospheric Pressure Chemical Ionization (APCI) or Electrospray Ionization (ESI) module. High resolution mass spectral (HRMS) data are obtained with direct analysis in real-time mass spectrometry (DART-MS).

Table of Abbreviations:

Abbreviation	
PTFE	Polytetrafluoroethylene or Teflon
t-BuOK	Potassium tert-butoxide
THF	Tetrahydrofuran
DCC	*N,N′*-Dicyclohexylcarbodiimide
DMAP	4-Dimethylaminopyridine
TFE	Trifluoroethanol
DCE	Dichloroethane
DME	Dimethoxyethane
DMF	Dimethylformamide
NMP	*N*-methylpyrrolidone
DMSO	Dimethyl sulfoxide
i-PrOH	Isopropanol (2-propanol)
TFA	Trifluoroacetic acid
EtOAc	Ethyl acetate

2. OPTIMIZATIONS

Table T1. Optimization of benzyl bromide with various diazenes:

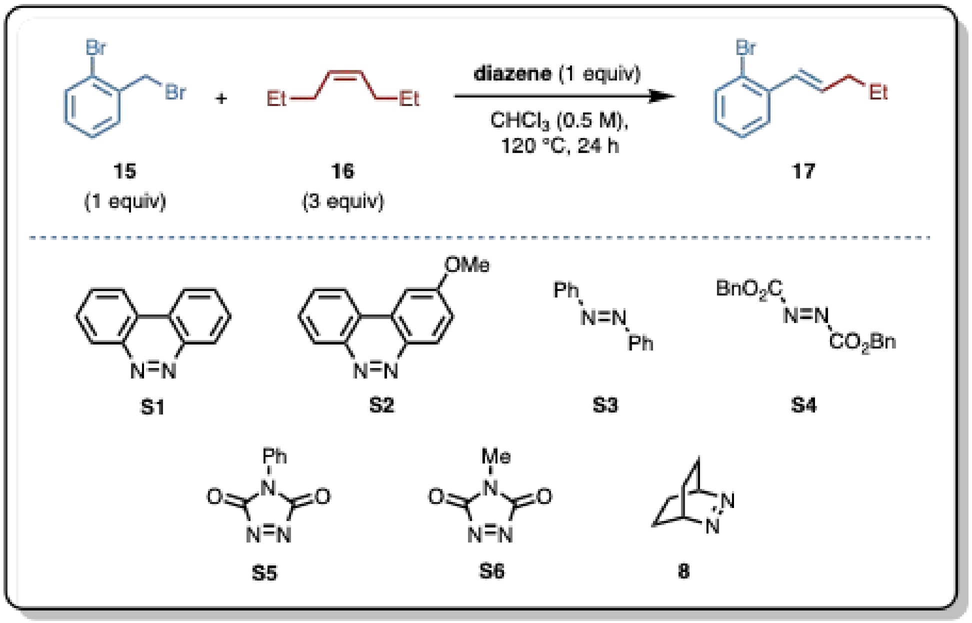

Entry	Diazene	Yield (%)*
1	--	n.d.
2	S1	n.d.
3	S2	n.d.
4	S3	n.d.
5	S4	n.d.
6	S5	n.d.
7	S6	n.d.
**8**	**8**	**62**

** yields determined by ^{1}H NMR spectroscopy with mesitylene as internal standard*

Table T2. Optimization (solvent screening):

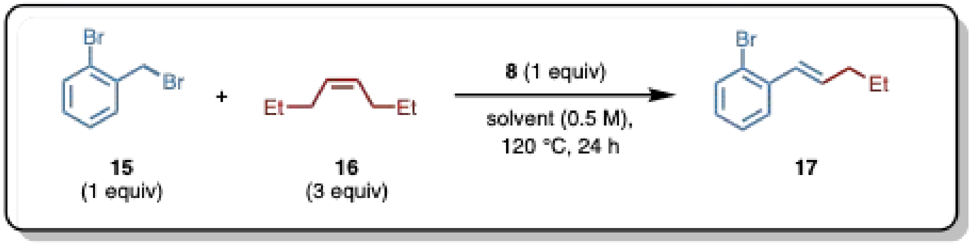

Entry	Solvent	Yield (%)*
**1**	**chloroform**	**62**
2	chlorobenzene	48
3	acetonitrile	37
4	tetrahydrofuran (THF)	34
5	isopropanol	29
6	ethanol	26
7	neat	20
8	ethyl acetate	14
9	dimethylformamide (DMF)	8
10	methanol	5
11	benzene	2
12	ethylene glycol	n.d.
13	1,4-dioxane	n.d.
14	dimethyl sulfoxide (DMSO)	n.d.
15	toluene	n.d.

yields determined by ^{1}H NMR spectroscopy with mesitylene as internal standard, n.d.= not detected

Table T3. Optimization (additive screening):

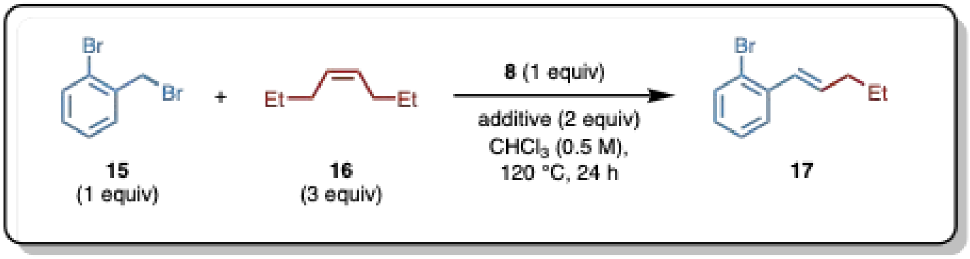

Entry	Additive	Yield (%)*
**1**	**none**	**62**
2	formic acid	20
3	acetic acid	30
4	trichloroacetic acid	6
5	trifluoroacetic acid (TFA)	29
6	p-toluenesulfonic acid	trace
7	triflic acid	n.d.
8	HBF4·H_2O	16
9	HBF4·Et_2O	n.d.
10	sulfuric acid	n.d.
11	perchloric acid	n.d.
12	HCl in acetic acid	11
13	HBr in acetic acid	n.d.

** yields determined by ^1H NMR spectroscopy with mesitylene as internal standard, n.d.= not detected*

Table T4. Optimization (substrate equivalents/concentration/temperature/time):

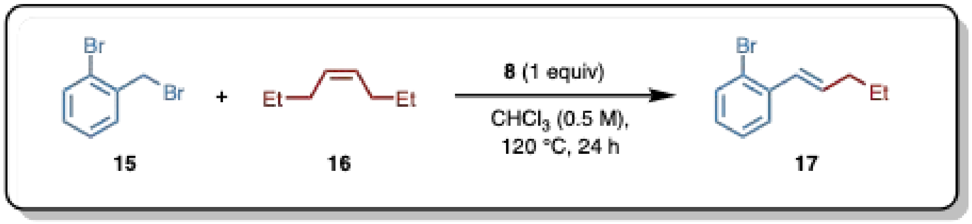

Entry	Olefin (equiv)	Halide (equiv)	Temp. (°C)	Conc. [M]	Time (h)	Yield (%)*
1	1.0	1.0	120	0.5	24	12
2	2.0	1.0	120	0.5	24	36
3	3.0	1.0	120	0.5	24	62
4	4.0	1.0	120	0.5	24	64
5	5.0	1.0	120	0.5	24	60
6	10.0	1.0	120	0.5	24	62
7	1.0	2.0	120	0.5	24	22
8	1.0	3.0	120	0.5	24	15
9	1.0	4.0	120	0.5	24	12
10	1.0	5.0	120	0.5	24	10
11	1.0	10.0	120	0.5	24	8
12	3.0	1.0	120	0.1	24	28
13	3.0	1.0	120	0.2	24	37
14	3.0	1.0	120	0.3	24	44
15	3.0	1.0	120	0.4	24	55
16	3.0	1.0	120	1.0	24	58
17	3.0	1.0	120	2.0	24	60
18	3.0	1.0	120	3.0	24	50
19	3.0	1.0	25	0.5	24	n.d.
20	3.0	1.0	40	0.5	24	n.d.
21	3.0	1.0	60	0.5	24	n.d.
22	3.0	1.0	70	0.5	24	n.d.
23	3.0	1.0	80	0.5	24	5
24	3.0	0.5	90	0.5	24	10
25	3.0	0.5	100	0.5	24	25
26	3.0	0.5	110	0.5	24	34
27	3.0	0.5	130	0.5	24	63
28	3.0	0.5	140	0.5	24	65
29	3.0	1.0	120	0.5	4	16
30	3.0	1.0	120	0.5	8	35
31	3.0	1.0	120	0.5	12	48
**32**	**3.0**	**1.0**	**120**	**0.5**	**36**	**84**
33	3.0	1.0	120	0.5	48	82
34	3.0	1.0	120	0.5	72	75
35	3.0	1.0	120	0.5	96	70

** yields determined by ^{1}H NMR spectroscopy with mesitylene as internal standard, n.d.= not detected*

Table T5. Optimization of benzyl alcohol with various diazenes:

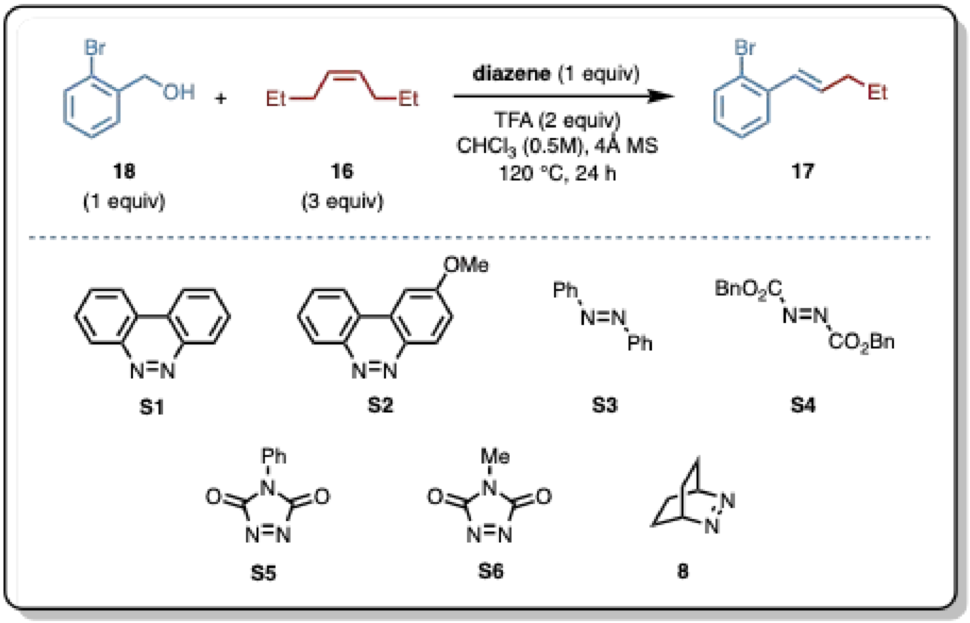

Entry	Diazene	Yield (%)*
1	--	n.d.
2	S1	n.d.
3	S2	n.d.
4	S3	n.d.
5	S4	n.d.
6	S5	n.d.
7	S6	n.d.
**8**	**8**	**55**

** yields determined by ^{1}H NMR spectroscopy with mesitylene as internal standard*

Table T6. Optimization (solvent screening):

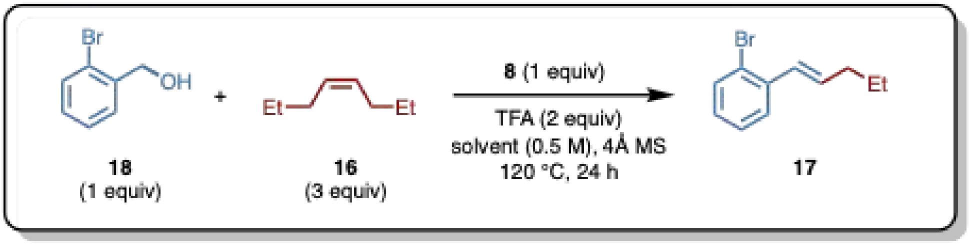

Entry	Solvent	Yield (%)*
**1**	**chloroform**	**55**
2	chlorobenzene	40
3	*n*-butanol	35
4	tetrahydrofuran (THF)	30
5	isopropanol	25
6	acetonitrile	24
7	ethanol	10
8	neat	9
9	dimethylformamide (DMF)	8
10	methanol	6
11	benzene	n.d.
12	ethylene glycol	n.d.
13	dimethyl sulfoxide (DMSO)	n.d.
14	1,4-dioxane	n.d.
15	toluene	n.d.

***** *yields determined by ^{1}H NMR spectroscopy with mesitylene as internal standard, n.d.= not detected***

Table T7. Optimization (acid screening):

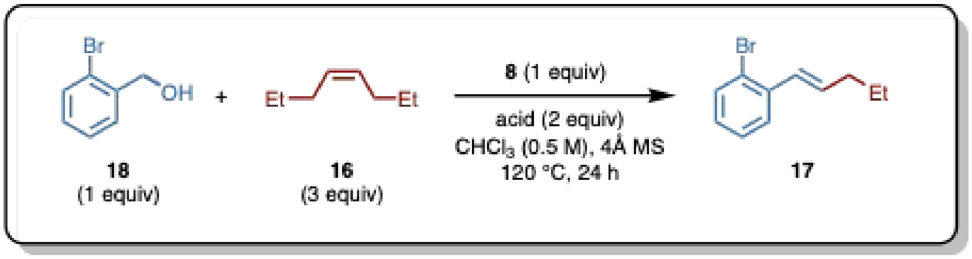

Entry	Additive	Yield (%)*
1	none	n.d.
2	formic acid	14
3	acetic acid	21
4	trichloroacetic acid	5
**5**	**trifluoroacetic acid (TFA)**	**55**
6	p-toluenesulfonic acid	8
7	triflic acid	n.d.
8	HBF4·H_2O	14
9	HBF4·Et_2O	n.d.
10	sulfuric acid	n.d.
11	perchloric acid	n.d.
12	HCl in acetic acid	18
13	HBr in acetic acid	n.d.

** yields determined by ^{1}H NMR spectroscopy with mesitylene as internal standard, n.d.= not detected*

Table T8. Optimization (substrate equivalents/concentration/temperature/time):

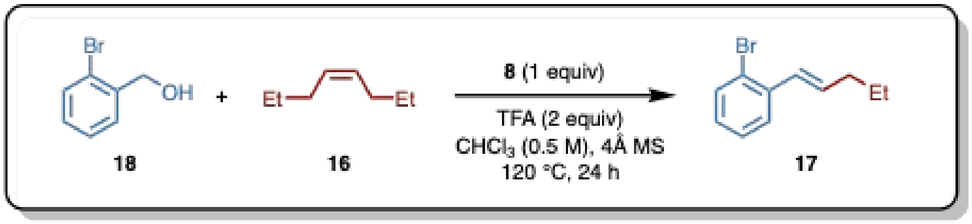

Entry	Olefin (equiv)	Halide (equiv)	Temp. (°C)	Conc. [M]	Time (h)	Yield (%)*
1	1.0	1.0	120	0.5	24	15
2	2.0	1.0	120	0.5	24	40
3	3.0	1.0	120	0.5	24	55
4	4.0	1.0	120	0.5	24	54
5	5.0	1.0	120	0.5	24	55
6	10.0	1.0	120	0.5	24	56
7	1.0	2.0	120	0.5	24	20
8	1.0	3.0	120	0.5	24	14
9	1.0	4.0	120	0.5	24	12
10	1.0	5.0	120	0.5	24	7
11	1.0	10.0	120	0.5	24	5
12	3.0	1.0	120	0.1	24	30
13	3.0	1.0	120	0.2	24	37
14	3.0	1.0	120	0.3	24	40
15	3.0	1.0	120	0.4	24	48
16	3.0	1.0	120	1.0	24	50
17	3.0	1.0	120	2.0	24	53
18	3.0	1.0	120	3.0	24	44
19	3.0	1.0	25	0.5	24	n.d.
20	3.0	1.0	40	0.5	24	n.d.
21	3.0	1.0	60	0.5	24	n.d.
22	3.0	1.0	70	0.5	24	n.d.
23	3.0	1.0	80	0.5	24	2
24	3.0	0.5	90	0.5	24	5
25	3.0	0.5	100	0.5	24	19
26	3.0	0.5	110	0.5	24	30
27	3.0	0.5	130	0.5	24	56
28	3.0	0.5	140	0.5	24	60
29	3.0	1.0	120	0.5	4	12
30	3.0	1.0	120	0.5	8	30
31	3.0	1.0	120	0.5	12	42
**32**	**3.0**	**1.0**	**120**	**0.5**	**36**	**75**
33	3.0	1.0	120	0.5	48	72
34	3.0	1.0	120	0.5	72	67
35	3.0	1.0	120	0.5	96	65

** yields determined by ^{1}H NMR spectroscopy with mesitylene as internal standard, n.d.= not detected*

Table T9. Substrate compatibility for *trans*-olefins and trisubstituted olefins:

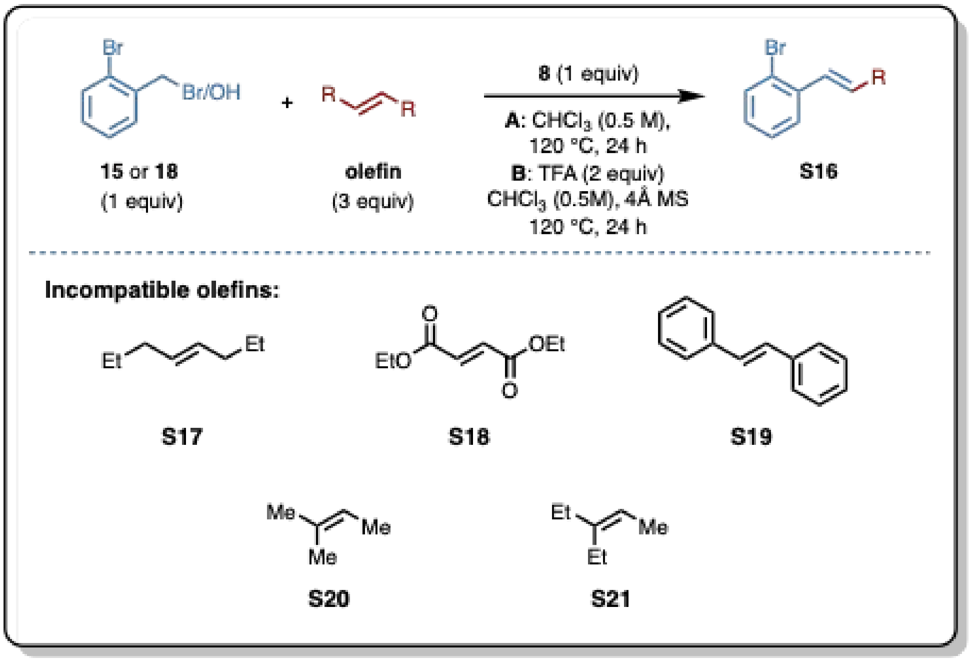

Entry	Electrophile	Olefin	Condition	Yield (%)*
1	15	S17	A	n.d.
2	15	S18	A	n.d.
3	15	S19	A	n.d.
4	15	S20	A	n.d.
5	15	S21	A	n.d.
6	18	S17	B	n.d.
7	18	S18	B	n.d.
8	18	S19	B	n.d.
9	18	S20	B	n.d.
10	18	S21	B	n.d.

** yields determined by ^{1}H NMR spectroscopy with mesitylene as internal standard*
n.d. = not detected

Table T10. Substrate compatibility for aliphatic and allylic electrophiles

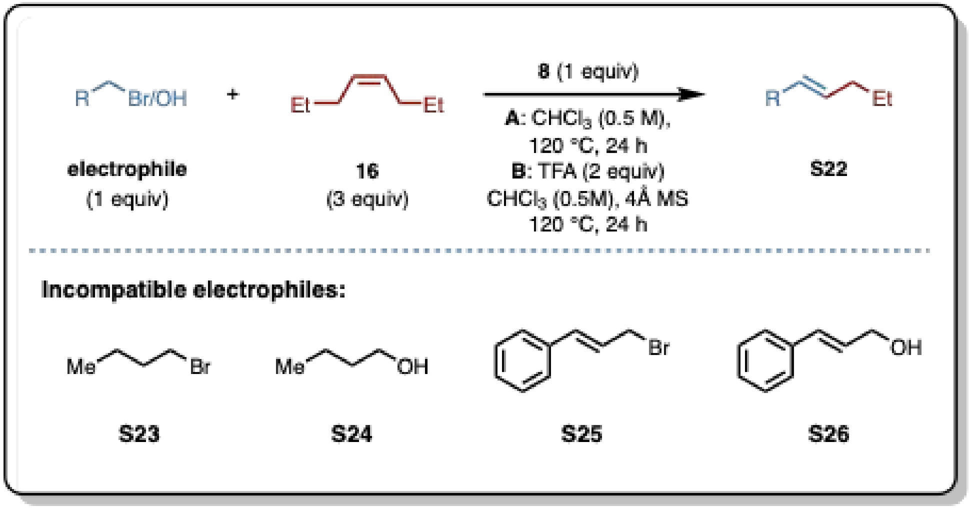

Entry	Electrophile	Condition	Yield (%)*
1	S23	A	n.d.
2	S24	B	n.d.
3	S25	A	n.d.
4	S26	B	n.d.

** yields determined by ^{1}H NMR spectroscopy with mesitylene as internal standard*
n.d. = not detected

Table T11. Substrate compatibility for secondary benzylic electrophiles

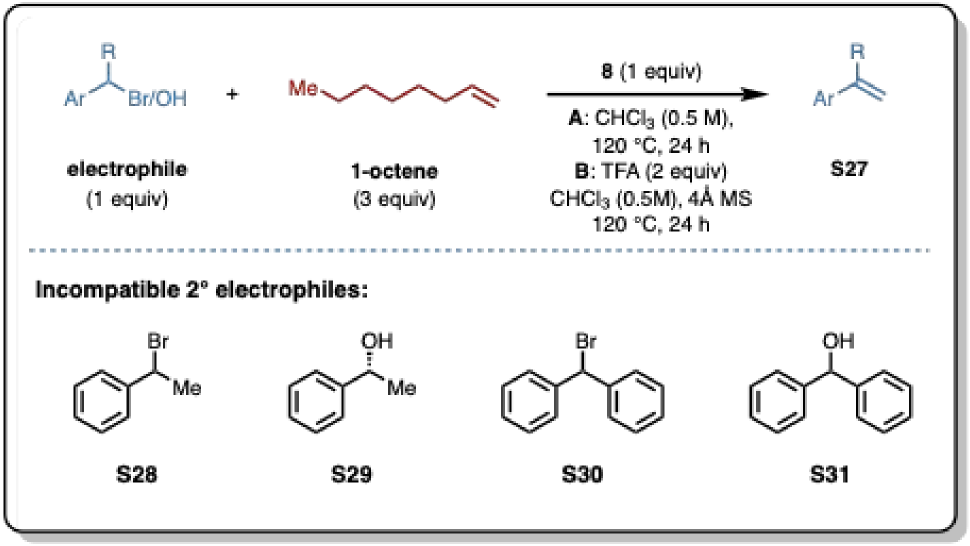

Entry	Electrophile	Condition	Yield (%)*
1	S28	A	n.d.
2	S29	B	n.d.
3	S30	A	n.d.
4	S31	B	n.d.

** yields determined by ^{1}H NMR spectroscopy with mesitylene as internal standard*
n.d. = not detected

Table T12. Attempts for catalytic AEOM reactivity:

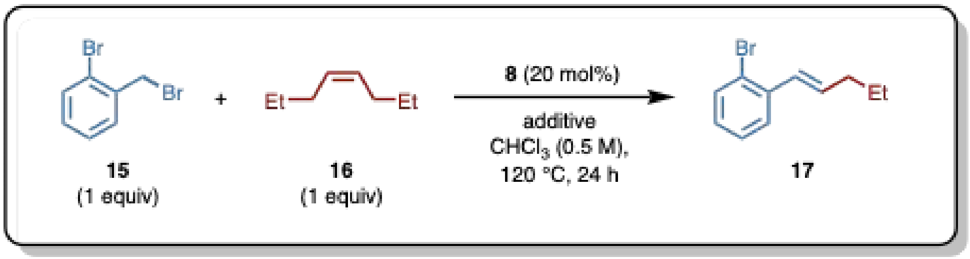

Entry	Additive	Yield (%)*
**1**	**none**	**18**
2	Et_3N (20 mol%)	11
3	Et_3N (40 mol%)	6
4	Et_3N (1 equiv)	trace
5	H_2O (20 mol%)	13
6	H_2O (40 mol%)	8
7	H_2O (1 equiv)	4
8	*i*-Pr_2NEt (20 mol%)	12
9	*i*-Pr_2NEt (40 mol%)	7
10	*i*-Pr_2NEt (1 equiv)	trace

***** yields determined by ^1H NMR spectroscopy with mesitylene as internal standard, n.d.= not detected***

3. DIAZENE SYNTHESIS

Other acyclic and cyclic diazenes used were commercially available from various vendors.

Synthesis of 2-methoxybenzo[*c*]cinnoline (S2):
Prepared according to our previous report.[1] The spectral and analytical data agreed with the reported values.

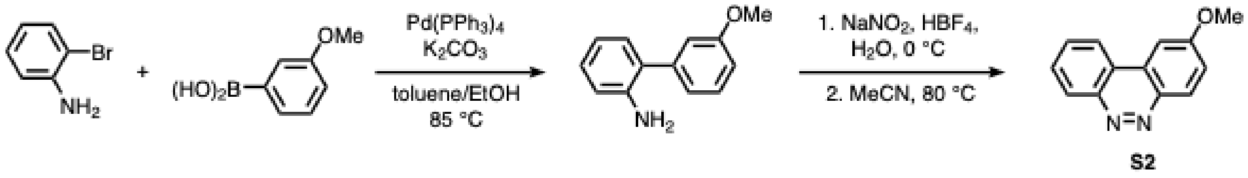

Synthesis of 4-phenyl-3*H*-1,2,4-triazole-3,5(4*H*)-dione (S5):
Prepared according to our previous report.[4] The spectral and analytical data agreed with the reported values.

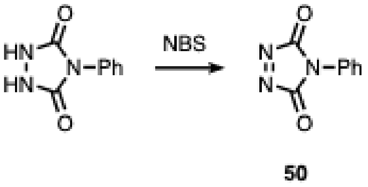

Synthesis of 4-methyl-3*H*-1,2,4-triazole-3,5(4*H*)-dione (S6):
Prepared according to literature report.[5] The spectral and analytical data agreed with the reported values.

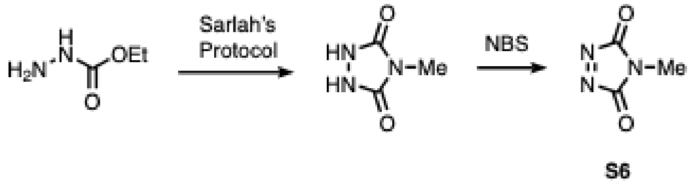

Synthesis of 2,3-Diazabicyclo[2.2.2]oct-2-ene (8):
Prepared according to our previous report.[4] The spectral and analytical data agreed with the reported values.

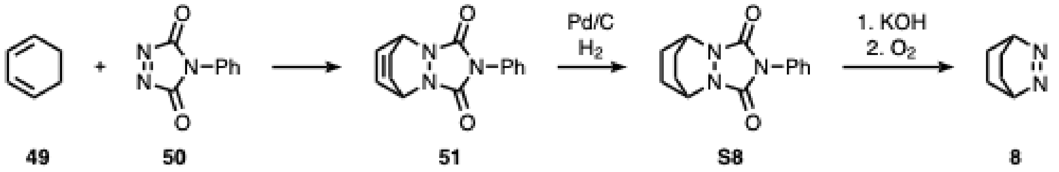

Gram-scale synthesis (8):

2-Phenyl-5,8-dihydro-1*H*-5,8-ethano[1,2,4]triazolo[1,2-*a*]pyridazine-1,3(2*H*)-dione (50):
In a 250 mL round bottom flask, 1,3-cyclohexadiene (6.6 mL, 68 mmol) was added dropwise slowly to a 1 M solution of 4-phenyl-3*H*-1,2,4-triazole-3,5(4*H*)-dione (PTAD) (12.0 g, 68 mmol) in acetone (68 mL). The color of the solution changed from bright red to dull yellow upon reaction completion. The reaction was left to stir for 1 h. Pentane (30 mL) was added into the solution mixture and the resulting white solid precipitate was collected and rinsed with pentane (30 mL) via vacuum filtration and dried *in vacuo* to furnish the title compound (17.2 g, 98% yield). The spectral and analytical data agreed with the reported values.[4]

^{1}H NMR (500 MHz, $CDCl_3$) δ 7.49 – 7.38 (m, 4H), 7.34 (dq, J = 5.5, 2.8 Hz, 1H), 6.73 – 6.26 (m, 2H), 4.95 (dt, J = 4.3, 1.5 Hz, 2H), 2.21 (dt, J = 9.1, 1.6 Hz, 2H), 1.60 (ddd, J = 11.2, 3.5, 2.0 Hz, 2H)
^{13}C NMR (126 MHz, $CDCl_3$) δ 156.3, 131.6, 130.7, 129.3, 128.4, 125.7, 50.4, 22.2

2-Phenyltetrahydro-1*H*-5,8-ethano[1,2,4]triazolo[1,2-*a*]pyridazine-1,3(2*H*)-dione (51):
Compound S1 (15.3 g, 60 mmol), Pd/C (0.3 g, 3.0 mmol) and MeOH (200 mL, 0.3 M) were combined in a 500 mL round bottom flask equipped with a stir-bar. The flask was purged 5 times with a balloon of H_2 and the reaction mixture was stirred vigorously under an atmosphere of H_2 for 16 h. The mixture was then filtered through Celite® and concentrated *in vacuo* to yield the title compound as a white solid (15.2 g, 99% yield). The spectral and analytical data agreed with the reported values.[4]

^{1}H NMR (500 MHz, $CDCl_3$) δ 7.54 (d, J = 7.6 Hz, 2H), 7.46 (t, J = 7.8 Hz, 2H), 7.35 (t, J = 7.5 Hz, 1H), 4.41 (s, 2H), 2.21 – 2.00 (m, 4H), 1.87 (d, J = 8.5 Hz, 4H)
^{13}C NMR (126 MHz, $CDCl_3$) δ 129.2, 128.1, 125.6, 48.2, 24.9

2,3-Diazabicyclo[2.2.2]oct-2-ene (8):
Compound S2 (13.4 g, 52 mmol) and *i*-PrOH (500 mL) were charged to a 1 L round bottom flask equipped with a stir-bar and a reflux condenser. The flask was purged with N_2 for 15 min. Solid KOH (15 equiv) pellets were added under a flow of N_2. The reaction was heated to reflux for 2 h and then cooled to room temperature. Air was bubbled into the reaction mixture overnight. The mixture was diluted with water and then extracted with CH_2Cl_2 (5 x 200 mL). The organic layers were combined and washed with 1 M aqueous HCl (150 mL). The organic layer was dried over Na_2SO_4, filtered, and carefully concentrated *in vacuo*. The resultant yellow solid was rinsed with hot pentane (2 x 100 mL). The colorless pentane solution was decanted carefully, leaving an amber residue behind. The combined pentane solution was then subjected to crystallization to furnish the title compound as a white solid (4.0 g, 70% yield). The spectral and analytical data agreed with the previously reported values.[4]

^{1}H NMR (500 MHz, $CDCl_3$) δ 5.10 (s, 2H), 1.57 (d, J = 7.9 Hz, 4H), 1.30 – 1.27 (m, 4H)
^{13}C NMR (126 MHz, $CDCl_3$) δ 61.1, 21.3

---

4. DIAZENE STABILITY

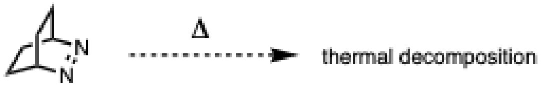

8

Entry	Temp. (°C)	Solvent	Time (h)	Recovery* (%)
1	25	$CHCl_3$	1	100
2	25	$CHCl_3$	2	100
3	25	$CHCl_3$	4	100
4	25	$CHCl_3$	8	100
5	25	$CHCl_3$	12	100
6	25	$CHCl_3$	24	100
7	40	$CHCl_3$	1	100
8	40	$CHCl_3$	2	100
9	40	$CHCl_3$	4	100
10	40	$CHCl_3$	8	100
11	40	$CHCl_3$	12	100
12	40	$CHCl_3$	24	100
13	60	$CHCl_3$	1	100
14	60	$CHCl_3$	2	100
15	60	$CHCl_3$	4	100
16	60	$CHCl_3$	8	100
17	60	$CHCl_3$	12	100
18	60	$CHCl_3$	24	100
19	80	$CHCl_3$	1	100
20	80	$CHCl_3$	2	100
21	80	$CHCl_3$	4	100
22	80	$CHCl_3$	8	100
23	80	$CHCl_3$	12	100
24	80	$CHCl_3$	24	100
25	100	$CHCl_3$	1	100
26	100	$CHCl_3$	2	100
27	100	$CHCl_3$	4	100
28	100	$CHCl_3$	8	100
29	100	$CHCl_3$	12	100
30	100	$CHCl_3$	24	100
31	120	$CHCl_3$	1	100
32	120	$CHCl_3$	2	100
33	120	$CHCl_3$	4	100
34	120	$CHCl_3$	8	100
35	120	$CHCl_3$	12	100
36	120	$CHCl_3$	24	100
37	120	toluene	1	100
38	120	toluene	2	100
39	120	toluene	4	100
40	120	toluene	8	100
41	120	toluene	12	100
42	120	toluene	24	100

***** decomposition determined by ^{1}H NMR spectroscopy with mesitylene as internal standard,*

5. SYNTHESIS OF ALCOHOLS

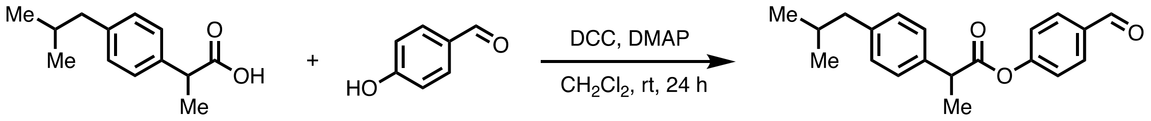

Compound (S7): 4-formylphenyl 2-(4-isobutylphenyl)propanoate

To a stirred solution of ibuprofen (1.03 g, 5.0 mmol, 1.0 equiv) in CH_2Cl_2 (25 mL, 0.2 M), 4-hydroxybenzaldehyde (733 mg, 6.0 mmol, 1.2 equiv), DMAP (61 mg, 0.50 mmol, 0.10 equiv) and DCC (1.55 g, 7.5 mmol, 1.5 equiv) were added under N_2 atmosphere. The mixture was stirred at room temperature overnight. After filtration through Celite®, the filtrate was concentrated and purified by silica gel chromatography (20% EtOAc/hexanes) to afford the title compound as a colorless oil (1.52 g, 98% yield). The spectral and analytical data agreed with the previously reported values.[2]

^{1}H NMR (500 MHz, $CDCl_3$) δ 9.96 (s, 1H), 7.91 – 7.82 (m, 2H), 7.32 – 7.28 (m, 2H), 7.21 – 7.13 (m, 4H), 3.97 (q, J = 7.1 Hz, 1H), 2.48 (d, J = 7.3 Hz, 2H), 1.87 (m, 1H), 1.62 (d, J = 7.2 Hz, 3H), 0.92 (d, J = 6.6 Hz, 6H)
^{13}C NMR (126 MHz, $CDCl_3$) δ 191.0, 172.7, 155.7, 141.2, 136.9, 134.0, 131.2, 129.7, 127.3, 122.3, 45.4, 45.1, 30.3, 22.5, 18.5
DART-MS m/z calcd for $C_{20}H_{23}O_3^+$ $[M + H]^+$ = 311.1642, found 311.1662

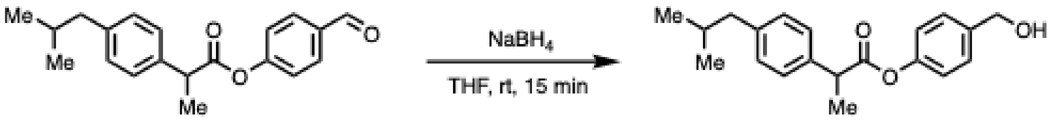

Compound (S8): 4-(hydroxymethyl)phenyl 2-(4-isobutylphenyl)propanoate

To a stirred solution of 4-formylphenyl 2-(4-isobutylphenyl)propanoate (310 mg, 1.0 mmol, 1.0 equiv) in THF (1.0 mL, 1.0 M), $NaBH_4$ (42 mg, 1.1 mmol, 1.1 equiv) was added under N_2 atmosphere. The mixture was stirred at room temperature for 15 min. After consumption of all starting material as determined by TLC, distilled water was added to the reaction mixture, and this solution was then stirred for an additional 5 min. The mixture was extracted with EtOAc (10 mL x 3) and the combined organic layers were dried over anhydrous sodium sulfate. The organic layer was concentrated and purified by silica gel chromatography (30% EtOAc/hexanes) to afford the title compound as a colorless oil (275 mg, 88% yield).

^{1}H NMR (500 MHz, $CDCl_3$) δ 7.40 – 7.27 (m, 4H), 7.17 – 7.12 (m, 2H), 7.02 – 6.94 (m, 2H), 4.66 (s, 2H), 3.94 (q, J = 7.1 Hz, 1H), 2.47 (d, J = 7.2 Hz, 2H), 1.87 (dh, J = 13.5, 6.8 Hz, 1H), 1.60 (d, J = 7.1 Hz, 3H), 0.91 (d, J = 6.6 Hz, 6H)
^{13}C NMR (126 MHz, $CDCl_3$) δ 173.4, 150.4, 141.0, 138.5, 137.3, 129.7, 128.1, 127.3, 121.7, 64.9, 45.4, 45.2, 30.3, 22.5, 18.7

DART-MS m/z calcd for $C_{20}H_{25}O_3^+$ $[M + H]^+$ = 313.1798, found 313.1801

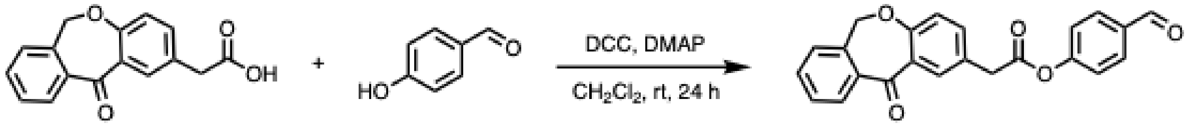

Compound (S9): 4-formylphenyl 2-(11-oxo-6,11-dihydrodibenzo[*b,e*]oxepin-2-yl)acetate

To a stirred solution of isoxepac (1.79 g, 5.0 mmol, 1.0 equiv) in CH_2Cl_2 (25 mL, 0.2 M), 4-hydroxybenzaldehyde (733 mg, 6.0 mmol, 1.2 equiv), DMAP (61 mg, 0.50 mmol, 0.10 equiv) and DCC (1.55 g, 7.5 mmol, 1.5 equiv) were added under N_2 atmosphere. The mixture was stirred at room temperature overnight. After filtration through Celite®, the filtrate was concentrated and purified by silica gel chromatography (10% EtOAc/hexanes) to afford the title compound as an off-white solid (1.79 g, 96% yield).

^{1}H NMR (500 MHz, $CDCl_3$) δ 9.98 (s, 1H), 8.23 (d, *J* = 2.4 Hz, 1H), 7.95 – 7.87 (m, 3H), 7.58 (td, *J* = 7.5, 1.4 Hz, 1H), 7.54 – 7.46 (m, 2H), 7.38 (dd, *J* = 7.4, 1.2 Hz, 1H), 7.31 – 7.26 (m, 2H), 7.09 (d, *J* = 8.4 Hz, 1H), 5.21 (s, 2H), 3.92 (s, 2H)
^{13}C NMR (126 MHz, $CDCl_3$) δ 191.0, 190.9, 169.3, 160.9, 155.4, 140.5, 136.4, 135.6, 134.2, 133.1, 132.8, 131.4, 129.7, 129.5, 128.0, 126.8, 125.4, 122.4, 121.6, 73.8, 40.4
DART-MS m/z calcd for $C_{23}H_{17}O_5^+$ $[M + H]^+$ = 373.1071, found 373.1092

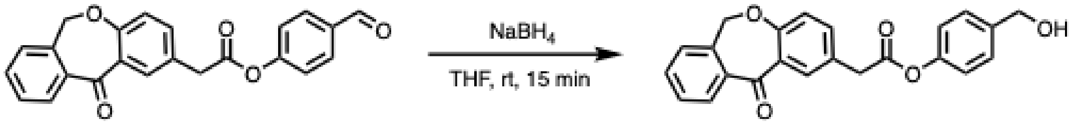

Compound (S10):
4-(hydroxymethyl)phenyl 2-(11-oxo-6,11-dihydrodibenzo[b,e]oxepin-2-yl)acetate

To a stirred solution of 4-formylphenyl 2-(11-oxo-6,11-dihydrodibenzo[*b,e*]oxepin-2-yl)acetate (372 mg, 1.0 mmol, 1.0 equiv) in THF (1.0 mL, 1.0 M), $NaBH_4$ (42 mg, 1.1 mmol, 1.1 equiv) were added under N_2 atmosphere. The mixture was stirred at room temperature for 15 min. After consumption of all starting material as determined by TLC, distilled water was added to the reaction mixture, and this solution was then stirred for an additional 5 min. The mixture was extracted with EtOAc (10 mL x 3), and the combined organic layers were dried over anhydrous sodium sulfate. After removal of solvent by rotary evaporation, the crude residue was purified by silica gel chromatography (40% EtOAc/hexanes) to afford the title compound as a colorless oil (352 mg, 94% yield).

^{1}H NMR (500 MHz, $CDCl_3$) δ 8.21 (d, *J* = 2.4 Hz, 1H), 7.88 (dd, *J* = 7.9, 1.4 Hz, 1H), 7.58 – 7.42 (m, 3H), 7.37 – 7.28 (m, 3H), 7.05 (t, *J* = 8.3 Hz, 3H), 5.17 (s, 2H), 4.61 (s, 2H), 3.87 (s, 2H), 2.31 (s, 1H)

^{13}C NMR (126 MHz, $CDCl_3$) δ 191.0, 170.1, 160.7, 150.0, 140.4, 138.8, 136.4, 135.6, 132.9, 132.7, 129.5, 129.4, 128.0, 127.9, 127.2, 125.3, 121.5, 121.3, 73.7, 64.6, 40.3
DART-MS m/z calcd for $C_{23}H_{19}O_5^+$ $[M + H]^+$ = 375.1227, found 375.1230

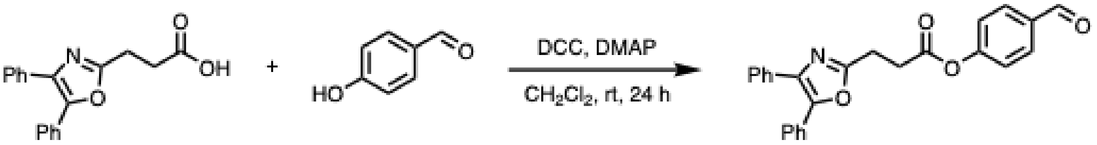

Compound (S11): 4-formylphenyl 3-(4,5-diphenyloxazol-2-yl)propanoate
To a stirred solution of oxaprozin (1.47 g, 5.0 mmol, 1.0 equiv) in CH_2Cl_2 (25 mL, 0.2 M), 4-hydroxybenzaldehyde (733 mg, 6.0 mmol, 1.2 equiv), DMAP (61 mg, 0.50 mmol, 0.10 equiv) and DCC (1.55 g, 7.5 mmol, 1.5 equiv) were added under N_2 atmosphere. The mixture was stirred at room temperature overnight. After filtration through Celite®, the filtrate was concentrated and purified by silica gel chromatography (30% EtOAc/hexanes) to afford the title compound as a white solid (1.95 g, 98% yield).

^{1}H NMR (500 MHz, $CDCl_3$) δ 9.99 (s, 1H), 7.93 – 7.87 (m, 2H), 7.65 (dt, J = 6.4, 1.5 Hz, 2H), 7.61 – 7.55 (m, 2H), 7.40 – 7.28 (m, 8H), 3.32 (td, J = 7.2, 0.9 Hz, 2H), 3.21 (td, J = 7.2, 1.0 Hz, 2H)
^{13}C NMR (126 MHz, $CDCl_3$) δ 191.1, 170.2, 161.3, 155.5, 145.8, 135.3, 134.2, 132.5, 131.4, 129.0, 128.8, 128.7, 128.3, 128.0, 126.7, 122.5, 31.4, 23.6
DART-MS m/z calcd for $C_{25}H_{20}NO_4^+$ $[M + H]^+$ = 398.1387, found 398.1405

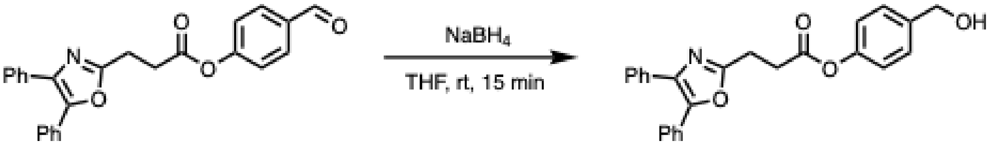

Compound (S12): 4-(hydroxymethyl)phenyl 3-(4,5-diphenyloxazol-2-yl)propanoate
To a stirred solution of 4-formylphenyl 3-(4,5-diphenyloxazol-2-yl)propanoate (397 mg, 1.0 mmol, 1.0 equiv) in THF (1.0 mL, 1.0 M), $NaBH_4$ (42 mg, 1.1 mmol, 1.1 equiv) were added under N_2 atmosphere. The mixture was stirred at room temperature for 15 min. After consumption of all starting material as determined by TLC, distilled water was added to the reaction mixture, and this solution was then stirred for an additional 5 min. The mixture was extracted with EtOAc (10 mL x 3) and dried over anhydrous sodium sulfate. The organic layer was concentrated and purified by silica gel chromatography (40% EtOAc/hexanes) to afford the title compound as a colorless oil (360 mg, 90% yield).

^{1}H NMR (500 MHz, $CDCl_3$) δ 7.68 – 7.62 (m, 2H), 7.61 – 7.55 (m, 2H), 7.41 – 7.29 (m, 9H), 7.12 – 7.06 (m, 2H), 4.66 (s, 2H), 3.30 (t, J = 7.5 Hz, 2H), 3.16 (t, J = 7.2 Hz, 2H)
^{13}C NMR (126 MHz, $CDCl_3$) δ 170.8, 161.6, 150.1, 145.7, 138.8, 135.3, 132.5, 129.0, 128.8, 128.7, 128.7, 128.3, 128.2, 128.0, 126.6, 121.7, 64.8, 31.4, 23.6

DART-MS m/z calcd for $C_{25}H_{22}NO_4^+$ $[M + H]^+$ = 400.1543, found 400.1544

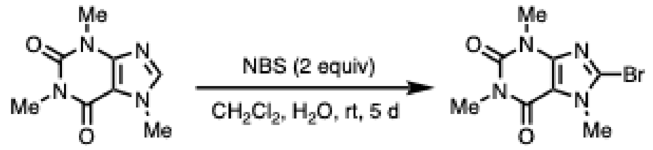

Compound (S13): 8-bromo-1,3,7-trimethyl-3,7-dihydro-1*H*-purine-2,6-dione
In a 500 mL round bottom flask, caffeine (1.94 g, 0.1 mol) and NBS (35.2 g, 0.2 mol) were added to 300 mL of CH_2Cl_2. After the dissolution of the solids, 100 mL of water was added. The mixture was stirred vigorously for 5 days at room temperature. Then cold 2 M NaOH solution (100 mL) was added to decolorize the solution. The organic layer was separated, washed with water (2 × 200 mL), dried over Na_2SO_4, filtered and concentrated *in vacuo* to afford the title compound as a white solid (26.2 g, 96% yield). The spectral and analytical data agreed with the previously reported values.[14]

^{1}H NMR (500 MHz, $CDCl_3$) δ 3.94 (s, 3H), 3.53 (s, 3H), 3.37 (s, 3H)
^{13}C NMR (126 MHz, $CDCl_3$) δ 154.5, 151.3, 148.1, 128.2, 109.4, 34.0, 29.9, 28.1
DART-MS m/z calcd for $C_8H_9BrN_4O_2^+$ $[M + H]^+$ = 271.9909, found 271.9930

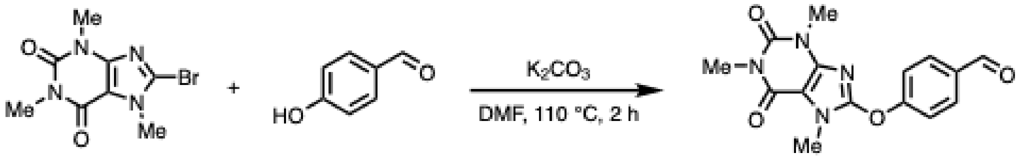

Compound (S14):
4-((1,3,7-trimethyl-2,6-dioxo-2,3,6,7-tetrahydro-1*H*-purin-8-yl)oxy)benzaldehyde
To a stirred solution of 8-bromo-1,3,7-trimethyl-3,7-dihydro-1*H*-purine-2,6-dione **S23** (1.37 g, 5.0 mmol, 1.0 equiv) in DMF (140 mL), 4-hydroxybenzaldehyde (611 mg, 5.0 mmol, 1.0 equiv), and K_2CO_3 (691 mg, 5.0 mmol, 1.0 equiv) were added under N_2 atmosphere. The mixture was stirred and heated at 110 ˚C for 2 hours. The reaction was cooled to room temperature and further cooled under an ice-bath. The formed solid was filtered and washed by cold hexanes to afford the title compound as a white solid (1.34 g, 85% yield). The spectral and analytical data agreed with the previously reported values.[15]

^{1}H NMR (500 MHz, $CDCl_3$) δ 10.00 (s, 1H), 7.95 (d, *J* = 8.3 Hz, 2H), 7.50 (d, *J* = 8.3 Hz, 2H), 3.88 (s, 3H), 3.45 (s, 3H), 3.39 (s, 3H)
^{13}C NMR (126 MHz, $CDCl_3$) δ 190.7, 157.8, 155.0, 152.2, 151.6, 145.7, 133.8, 131.8, 119.7, 104.1, 30.7, 30.0, 28.0
DART-MS m/z calcd for $C_{15}H_{15}N_4O_4^+$ $[M + H]^+$ = 315.1088, found 315.1106

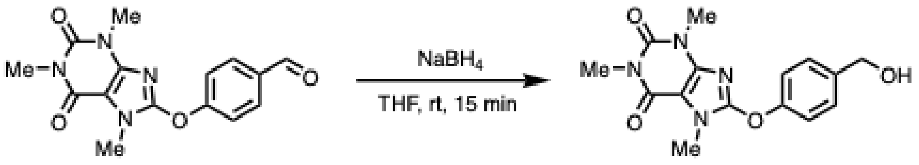

Compound (S15):
8-(4-(hydroxymethyl)phenoxy)-1,3,7-trimethyl-3,7-dihydro-1*H*-purine-2,6-dione

To a stirred solution of 4-((1,3,7-trimethyl-2,6-dioxo-2,3,6,7-tetrahydro-1*H*-purin-8-yl)oxy)benzaldehyde (314 mg, 1.0 mmol, 1.0 equiv) in THF (1.0 mL, 1.0 M), $NaBH_4$ (42 mg, 1.1 mmol, 1.1 equiv) were added under N_2 atmosphere. The mixture was stirred at room temperature for 15 min. After consumption of all starting material as determined by TLC, distilled water was added to the reaction mixture, and this solution was then stirred for an additional 5 min. The mixture was extracted with EtOAc (10 mL x 3) and dried over anhydrous sodium sulfate. The organic layer was concentrated and purified by recrystallization (diethyl ether) to afford the title compound as a white solid (291 mg, 92% yield).

^{1}H NMR (500 MHz, DMSO) δ 7.42 – 7.36 (m, 2H), 7.33 – 7.27 (m, 2H), 5.28 (s, 1H), 4.51 (s, 2H), 3.76 (s, 3H), 3.25 (s, 3H), 3.21 (s, 3H)
^{13}C NMR (126 MHz, DMSO) δ 154.0, 153.4, 152.1, 150.9, 145.3, 140.1, 127.9, 119.5, 103.1, 62.3, 30.2, 29.5, 27.5
DART-MS m/z calcd for $C_{15}H_{17}N_4O_4^+$ $[M + H]^+$ = 317.1244, found 317.1264

6. PRODUCT SYNTHESIS AND CHARACTERIZATION

General Procedure A (for alkyl halides):

To a flame-dried 1 dram (4 mL) vial, diazene (0.2 mmol, 1.0 equiv), benzylic halide (1.0 equiv), olefin (3.0 equiv), and dry solvent (0.5 M) were added under nitrogen atmosphere. A PTFE-silicone septa-lined cap was fixed onto the vial and was secured with electrical tape to prevent solvent from escaping. The solution was stirred for 24 to 48 h at 120 °C. The reaction was then cooled to room temperature, concentrated *in vacuo*, and purified by silica gel chromatography (FCC) or PrepTLC to afford the desired product. When the desired olefin product was volatile, yields were determined by quantitative ^{1}H NMR using mesitylene as internal standard. *This procedure was followed unless otherwise noted below.*

General Procedure B (for alcohols):

To a flame-dried 1 dram (4 mL) vial, diazene (0.2 mmol, 1.0 equiv), benzylic alcohol (1.0 equiv), olefin (3.0 equiv), trifluoroacetic acid (2.0 equiv), 4Å molecular sieves (MS), and dry solvent (0.5 M) were added under nitrogen atmosphere. A PTFE-silicone septa-lined cap was fixed onto the vial and was secured with electrical tape to prevent solvent from escaping. The solution was stirred for 24 to 48 h at 120 °C. The reaction mixture was then cooled to room temperature, concentrated *in vacuo*, and purified by silica gel chromatography (FCC) or PrepTLC to afford the desired product. When the desired olefin product was volatile, yields were determined by quantitative ^{1}H NMR using mesitylene as internal standard. *This procedure was followed unless otherwise noted below.*

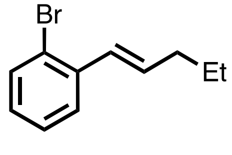

Compound (17): (*E*)-1-bromo-2-(pent-1-en-1-yl)benzene
Prepared according to Procedure A from 2-bromobenzyl bromide (0.2 mmol, 1.0 equiv), *cis*-4-octene (0.6 mmol, 3.0 equiv) in dry $CHCl_3$ (0.5 M) over 24 h to afford the title compound as a colorless oil (39 mg, 84% yield). FCC solvent: pentane

Prepared according to Procedure A from 2-bromobenzyl chloride (0.2 mmol, 1.0 equiv), *cis*-4-octene (0.6 mmol, 3.0 equiv) in dry $CHCl_3$ (0.5 M) over 24 h to afford the title compound as a colorless oil (39 mg, 68% yield). FCC solvent: pentane

^{1}H NMR (500 MHz, $CDCl_3$) δ 7.52 (dd, *J* = 8.0, 1.3 Hz, 1H), 7.49 (dd, *J* = 7.8, 1.7 Hz, 1H), 7.26 – 7.21 (m, 1H), 7.05 (td, *J* = 7.7, 1.7 Hz, 1H), 6.71 (dt, *J* = 15.7, 1.7 Hz, 1H), 6.17 (dt, *J* = 15.7, 7.0 Hz, 1H), 2.24 (qd, *J* = 7.2, 1.6 Hz, 2H), 1.53 (sext, *J* = 7.2 Hz, 2H), 0.98 (t, *J* = 7.4 Hz, 3H)
^{13}C NMR (126 MHz, $CDCl_3$) δ 137.9, 134.2, 132.9, 128.9, 128.2, 127.5, 127.0, 123.3, 35.3, 22.5, 13.9
DART-MS m/z calcd for $C_{11}H_{14}BrO^+$ $[M + OH]^+$ = 241.0223, found 241.0243

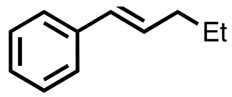

Compound (19): (*E*)-pent-1-en-1-ylbenzene
Prepared according to Procedure B from benzyl alcohol (0.2 mmol, 1.0 equiv), *cis*-4-octene (0.6 mmol, 3.0 equiv), trifluoroacetic acid (2.0 equiv), 4Å molecular sieves (MS) in dry $CHCl_3$ (0.5 M) over 36 h to afford the title compound as a colorless oil (20 mg, 69% yield). FCC solvent: pentane

Prepared according to Procedure A from benzyl bromide (0.2 mmol, 1.0 equiv), *cis*-4-octene (0.6 mmol, 3.0 equiv) in dry $CHCl_3$ (0.5 M) over 36 h to afford the title compound as a colorless oil (22 mg, 74% yield). FCC solvent: pentane

Prepared according to Procedure A from benzyl chloride (0.2 mmol, 1.0 equiv), *cis*-4-octene (0.6 mmol, 3.0 equiv) in dry $CHCl_3$ (0.5 M) over 36 h to afford the title compound as a colorless oil (18 mg, 60% yield). FCC solvent: pentane

^{1}H NMR (500 MHz, $CDCl_3$) δ 7.36 – 7.33 (m, 2H), 7.29 (dd, J = 8.5, 6.9 Hz, 2H), 7.22 – 7.16 (m, 1H), 6.39 (dt, J = 15.9, 1.5 Hz, 1H), 6.23 (dt, J = 15.8, 6.9 Hz, 1H), 2.19 (qd, J = 7.0, 1.5 Hz, 2H), 1.50 (sext, J = 7.3 Hz, 2H), 0.96 (t, J = 7.4 Hz, 3H)
^{13}C NMR (126 MHz, $CDCl_3$) δ 138.1, 131.1, 130.0, 128.6, 126.9, 126.1, 35.3, 22.7, 13.9
DART-MS m/z calcd for $C_{11}H_{15}O^+$ $[M + OH]^+$ = 163.1117, found 163.1124

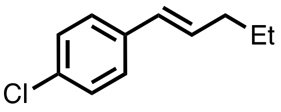

Compound (20): (*E*)-1-chloro-4-(pent-1-en-1-yl)benzene
Prepared according to Procedure B from 4-chlorobenzyl alcohol (0.2 mmol, 1.0 equiv), *cis*-4-octene (0.6 mmol, 3.0 equiv), trifluoroacetic acid (2.0 equiv), 4Å molecular sieves (MS) in dry $CHCl_3$ (0.5 M) over 24 h to afford the title compound as a colorless oil (30 mg, 82% yield). FCC solvent: pentane

Prepared according to Procedure A from 4-chlorobenzyl bromide (0.2 mmol, 1.0 equiv), *cis*-4-octene (0.6 mmol, 3.0 equiv) in dry $CHCl_3$ (0.5 M) over 36 h to afford the title compound as a colorless oil (31 mg, 85% yield). FCC solvent: pentane

Prepared according to Procedure A from 4-chlorobenzyl chloride (0.2 mmol, 1.0 equiv), *cis*-4-octene (0.6 mmol, 3.0 equiv) in dry $CHCl_3$ (0.5 M) over 36 h to afford the title compound as a colorless oil (26 mg, 72% yield). FCC solvent: pentane

^{1}H NMR (500 MHz, $CDCl_3$) δ 7.28 – 7.22 (m, 4H), 6.33 (dt, *J* = 15.8, 1.4 Hz, 1H), 6.20 (dt, *J* = 15.8, 6.8 Hz, 1H), 2.18 (qd, *J* = 7.0, 1.4 Hz, 2H), 1.49 (sext, J = 7.4 Hz, 2H), 0.95 (t, *J* = 7.3 Hz, 3H)
^{13}C NMR (126 MHz, $CDCl_3$) δ 136.6, 132.4, 131.9, 128.9, 128.7, 127.3, 35.2, 22.6, 13.9
DART-MS m/z calcd for $C_{11}H_{14}ClO^+$ $[M + OH]^+$ = 197.0728, found 197.0730

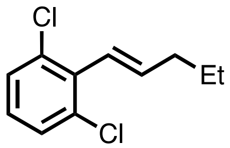

Compound (21): (*E*)-1,3-dichloro-2-(pent-1-en-1-yl)benzene
Prepared according to Procedure A from 2-(bromomethyl)-1,3-dichlorobenzene (0.2 mmol, 1.0 equiv), *cis*-4-octene (0.6 mmol, 3.0 equiv) in dry $CHCl_3$ (0.5 M) over 24 h to afford the title compound as a colorless oil (35 mg, 82% yield). FCC solvent: pentane

^{1}H NMR (500 MHz, $CDCl_3$) δ 7.29 (d, *J* = 8.0 Hz, 2H), 7.05 (t, *J* = 8.0 Hz, 1H), 6.34 (dt, *J* = 16.1, 1.5 Hz, 1H), 6.20 (dt, *J* = 16.2, 6.8 Hz, 1H), 2.27 (qd, *J* = 7.1, 1.5 Hz, 2H), 1.55 (sext, J = 7.3 Hz, 2H), 1.00 (t, *J* = 7.4 Hz, 3H)
^{13}C NMR (126 MHz, $CDCl_3$) δ 139.8, 135.5, 134.5, 128.4, 127.7, 123.7, 35.6, 22.3, 13.8
DART-MS m/z calcd for $C_{11}H_{13}Cl_2O^+$ $[M + OH]^+$ = 231.0338, found 231.0358

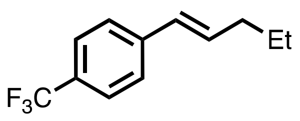

Compound (22): (*E*)-1-(pent-1-en-1-yl)-4-(trifluoromethyl)benzene
Prepared according to Procedure A from 4-(trifluoromethyl)benzyl bromide (0.2 mmol, 1.0 equiv), *cis*-4-octene (0.6 mmol, 3.0 equiv) in dry $CHCl_3$ (0.5 M) over 24 h to afford the title compound as a pale-yellow oil (38 mg, 88% yield). FCC solvent: pentane

Prepared according to Procedure A from 4-(trifluoromethyl)benzyl chloride (0.2 mmol, 1.0 equiv), *cis*-4-octene (0.6 mmol, 3.0 equiv) in dry $CHCl_3$ (0.5 M) over 24 h to afford the title compound as a pale-yellow oil (30 mg, 70% yield). FCC solvent: pentane

^{1}H NMR (500 MHz, $CDCl_3$) δ 7.54 (d, J = 8.1 Hz, 2H), 7.43 (d, J = 8.1 Hz, 2H), 6.41 (d, J = 15.9 Hz, 1H), 6.33 (dt, J = 15.8, 6.6 Hz, 1H), 2.22 (q, J = 7.1 Hz, 2H), 1.51 (sext, J = 7.4 Hz, 2H), 0.97 (t, J = 7.4 Hz, 3H)
^{13}C NMR (126 MHz, $CDCl_3$) δ 141.6 (d, J = 1.6 Hz), 134.0, 128.7 (q, J = 32.4 Hz), 126.2, 125.6 (q, J = 3.9 Hz), 123.4 (q, J = 271.8 Hz), 35.3, 22.5, 13.9
^{19}F NMR (470 MHz, $CDCl_3$) δ -62.38.
DART-MS m/z calcd for $C_{12}H_{14}F_3O^+$ $[M + OH]^+$ = 231.0991, found 231.1010

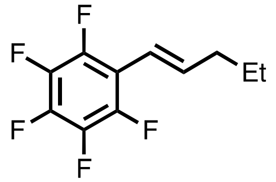

Compound (23): (*E*)-1,2,3,4,5-pentafluoro-6-(pent-1-en-1-yl)benzene
Prepared according to Procedure B from (perfluorophenyl)methanol (0.2 mmol, 1.0 equiv), *cis*-4-octene (0.6 mmol, 3.0 equiv), trifluoroacetic acid (2.0 equiv), 4Å molecular sieves (MS) in dry $CHCl_3$ (0.5 M) over 24 h to afford the title compound as a colorless oil (42 mg, 89% yield). FCC solvent: pentane

Prepared according to Procedure A from 2,3,4,5,6-pentafluorobenzyl bromide (0.2 mmol, 1.0 equiv), *cis*-4-octene (0.6 mmol, 3.0 equiv) in dry $CHCl_3$ (0.5 M) over 24 h to afford the title compound as a colorless oil (45 mg, 95% yield).

Increased scale preparation: Prepared according to the Procedure A from 2,3,4,5,6-pentafluorobenzyl bromide (1.0 mmol, 1.0 equiv), cis-4-octene (3.0 mmol, 3.0 equiv) in dry $CHCl_3$ (0.5 M) over 24 h to afford the title compound as a colorless oil (218 mg, 92% yield).

^{1}H NMR (500 MHz, $CDCl_3$) δ 6.55 (dt, J = 16.3, 7.0 Hz, 1H), 6.26 (dt, J = 16.4, 1.6 Hz, 1H), 2.25 (qd, J = 7.2, 1.3 Hz, 2H), 1.52 (sext, J = 7.4 Hz, 2H), 0.96 (t, J = 7.3 Hz, 3H)

^{13}**C NMR** (126 MHz, $CDCl_3$) δ 146.0 – 145.2 (m), 143.7 (m), 141.2 (m), 140.4 (m), 138.8 (m), 136.8 (m), 114.3 (d, *J* = 2.7 Hz), 113.1 – 112.1 (m), 36.5, 22.2, 13.8
^{19}**F NMR** (470 MHz, $CDCl_3$) δ -143.90 (dd, *J* = 21.8, 7.9 Hz), -158.08 (t, *J* = 20.8 Hz), -163.55 (td, *J* = 21.4, 7.9 Hz)
DART-MS m/z calcd for $C_{11}H_{10}F_5O^+$ $[M + OH]^+$ = 253.0646, found 253.0668

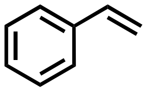

Compound (24): styrene
Prepared according to Procedure A from benzyl bromide (0.2 mmol, 1.0 equiv), 1-octene (0.6 mmol, 3.0 equiv) in dry $CHCl_3$ (0.5 M) over 24 h to afford the title compound characterized by quantitative ^{1}H NMR (55% yield). The spectral and analytical data agreed with the previously reported values.[6]

Prepared according to Procedure A from benzyl tosylate (0.2 mmol, 1.0 equiv), 1-octene (0.6 mmol, 3.0 equiv) in dry $CHCl_3$ (0.5 M) over 24 h to afford the title compound characterized by quantitative ^{1}H NMR (62% yield).

^{1}**H NMR** (500 MHz, $CDCl_3$) δ 7.42 (d, *J* = 8.1 Hz, 2H), 7.33 (t, *J* = 7.2 Hz, 2H), 7.27 (d, *J* = 7.1 Hz, 1H), 6.73 (dd, *J* = 17.6, 10.8 Hz, 1H), 5.76 (d, *J* = 17.6 Hz, 1H), 5.25 (d, *J* = 10.9 Hz, 1H)
^{13}**C NMR** (126 MHz, $CDCl_3$) δ 137.7, 137.0, 128.7, 127.9, 126.3, 114.0
DART-MS m/z calcd for $C_8H_9O^+$ $[M + OH]^+$ = 121.0648, found 121.0654

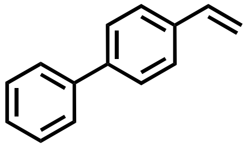

Compound (25): 4-vinyl-1,1'-biphenyl
Prepared according to Procedure A from 4-phenylbenzyl bromide (0.2 mmol, 1.0 equiv), 1-octene (0.6 mmol, 3.0 equiv) in dry $CHCl_3$ (0.5 M) over 24 h to afford the title compound as a white solid (27 mg, 74% yield). The spectral and analytical data agreed with the previously reported values.[7]
FCC solvent: pentane

^{1}**H NMR** (500 MHz, $CDCl_3$) δ 7.63 – 7.56 (m, 4H), 7.51 – 7.47 (m, 2H), 7.44 (dd, *J* = 8.4, 6.9 Hz, 2H), 7.37 – 7.31 (m, 1H), 6.76 (dd, *J* = 17.6, 10.9 Hz, 1H), 5.80 (dd, *J* = 17.6, 0.9 Hz, 1H), 5.28 (dd, *J* = 10.8, 0.9 Hz, 1H)
^{13}**C NMR** (126 MHz, $CDCl_3$) δ 140.9, 140.7, 136.7, 136.6, 128.9, 127.5, 127.4, 127.1, 126.8, 114.1
DART-MS m/z calcd for $C_{14}H_{13}O$ $[M + OH]^+$ = 197.0961, found 197.0968

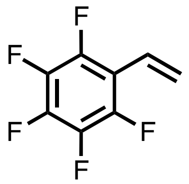

Compound (26): 1,2,3,4,5-pentafluoro-6-vinylbenzene
Prepared according to Procedure A from 2,3,4,5,6-pentafluorobenzyl bromide (0.2 mmol, 1.0 equiv), 1-octene (0.6 mmol, 3.0 equiv) in dry $CHCl_3$ (0.5 M) over 24 h to afford the title compound characterized by quantitative ^{1}H NMR (84% yield). The spectral and analytical data agreed with the previously reported values.[8] FCC solvent: pentane

^{1}H NMR (500 MHz, $CDCl_3$) δ 6.62 (dd, *J* = 18.0, 11.9 Hz, 1H), 6.08 (d, *J* = 18.0 Hz, 1H), 5.71 (d, *J* = 11.9 Hz, 1H)
^{13}C NMR (126 MHz, $CDCl_3$) δ 146.1 (tt, *J* = 7.8, 3.9 Hz), 144.1 (ddt, *J* = 11.7, 7.9, 3.8 Hz), 141.5 – 139.0 (m), 139.0 – 138.6 (m), 137.0 – 136.6 (m), 123.7 (td, *J* = 7.8, 2.5 Hz), 121.6 (q, *J* = 2.3 Hz), 112.4 (td, *J* = 13.9, 4.3 Hz)
^{19}F NMR (470 MHz, $CDCl_3$) δ -143.50 (dd, *J* = 21.4, 8.3 Hz), -155.20 – -158.65 (m), -163.18 (tdd, *J* = 20.2, 12.9, 7.8 Hz)
DART-MS m/z calcd for $C_8H_4F_5O$ $[M + OH]^+$ = 211.0177, found 211.0185

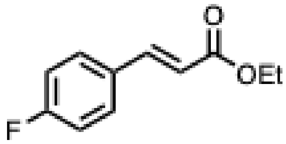

Compound (27): ethyl (*E*)-3-(4-fluorophenyl)acrylate
Prepared according to Procedure A from 4-fluorobenzyl bromide (0.2 mmol, 1.0 equiv), diethyl maleate (0.6 mmol, 3.0 equiv) in dry $CHCl_3$ (0.5 M) over 24 h to afford the title compound as a colorless oil (29 mg, 75% yield). FCC solvent: 5% EtOAc/hexanes

^{1}H NMR (500 MHz, $CDCl_3$) δ 7.64 (d, *J* = 16.0 Hz, 1H), 7.55 – 7.46 (m, 2H), 7.07 (t, *J* = 8.6 Hz, 2H), 6.36 (d, *J* = 16.0 Hz, 1H), 4.26 (q, *J* = 7.1 Hz, 2H), 1.34 (t, *J* = 7.1 Hz, 3H)
^{13}C NMR (126 MHz, $CDCl_3$) δ 167.0, 164.0 (d, *J* = 251.2 Hz), 143.4, 130.86 (d, *J* = 3.4 Hz), 130.0 (d, *J* = 8.5 Hz), 118.2 (d, *J* = 2.4 Hz), 116.2 (d, *J* = 21.8 Hz), 60.7, 14.5
^{19}F NMR (470 MHz, $CDCl_3$) δ -109.76 (td, *J* = 8.7, 4.5 Hz)
DART-MS m/z calcd for $C_{11}H_{12}FO_2^+$ $[M + H]^+$ = 195.0816, found 195.0818

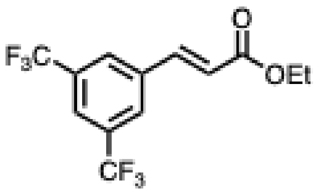

Compound (28): ethyl (*E*)-3-(3,5-bis(trifluoromethyl)phenyl)acrylate
Prepared according to Procedure A from 1-(bromomethyl)-3,5-bis(trifluoromethyl)benzene (0.2 mmol, 1.0 equiv), diethyl maleate (0.6 mmol, 3.0 equiv) in dry $CHCl_3$ (0.5 M) over 24 h to afford the title compound as a colorless oil (49 mg, 79% yield). FCC solvent: 5% EtOAc/hexanes

^{1}H NMR (500 MHz, $CDCl_3$) δ 7.69 (d, *J* = 16.1 Hz, 1H), 7.65 – 7.59 (m, 4H), 6.51 (d, *J* = 16.0 Hz, 1H), 4.28 (q, *J* = 7.1 Hz, 2H), 1.35 (t, *J* = 7.1 Hz, 3H)
^{13}C NMR (126 MHz, $CDCl_3$) δ 166.6, 142.8, 138.0 (d, *J* = 1.5 Hz), 131.9 (q, *J* = 32.7 Hz), 128.3, 126.0 (q, *J* = 3.8 Hz), 124.0 (q, *J* = 272.2 Hz), 121.0, 61.0, 14.4
^{19}F NMR (470 MHz, $CDCl_3$) δ -62.88
DART-MS m/z calcd for $C_{13}H_{11}F_6O_2^+$ $[M + H]^+$ = 313.0658, found 313.0670

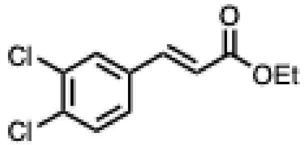

Compound (29): ethyl (*E*)-3-(3,4-dichlorophenyl)acrylate
Prepared according to Procedure A from 4-(bromomethyl)-1,2-dichlorobenzene (0.2 mmol, 1.0 equiv), diethyl maleate (0.6 mmol, 3.0 equiv) in dry $CHCl_3$ (0.5 M) over 24 h to afford the title compound as a colorless oil (40 mg, 82% yield). FCC solvent: 5% EtOAc/hexanes

^{1}H NMR (500 MHz, $CDCl_3$) δ 7.60 (d, *J* = 2.1 Hz, 1H), 7.56 (d, *J* = 16.0 Hz, 1H), 7.45 (d, *J* = 8.3 Hz, 1H), 7.34 (dd, *J* = 8.3, 2.1 Hz, 1H), 6.41 (d, *J* = 16.0 Hz, 1H), 4.27 (q, *J* = 7.1 Hz, 2H), 1.33 (t, *J* = 7.2 Hz, 3H)
^{13}C NMR (126 MHz, $CDCl_3$) δ 166.5, 141.9, 134.7, 134.3, 133.4, 131.0, 129.7, 127.1, 120.3, 60.9, 14.4
DART-MS m/z calcd for $C_{11}H_{11}Cl_2O_2^+$ $[M + H]^+$ = 245.0131, found 245.0150

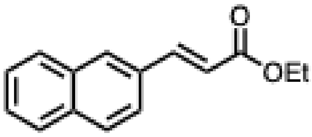

Compound (30): ethyl (*E*)-3-(naphthalen-2-yl)acrylate
Prepared according to Procedure A from 2-(bromomethyl)naphthalene (0.2 mmol, 1.0 equiv), diethyl maleate (0.6 mmol, 3.0 equiv) in dry $CHCl_3$ (0.5 M) over 24 h to afford the title compound as a colorless oil (33 mg, 73% yield). FCC solvent: 5% EtOAc/hexanes

^{1}H NMR (500 MHz, $CDCl_3$) δ 7.93 (s, 1H), 7.89 – 7.80 (m, 4H), 7.67 (dd, J = 8.7, 1.8 Hz, 1H), 7.55 – 7.49 (m, 2H), 6.56 (d, J = 16.0 Hz, 1H), 4.30 (q, J = 7.1 Hz, 2H), 1.37 (t, J = 7.1 Hz, 3H)
^{13}C NMR (126 MHz, $CDCl_3$) δ 167.2, 144.8, 134.3, 133.4, 132.1, 130.0, 128.8, 128.7, 127.9, 127.3, 126.8, 123.6, 118.6, 60.7, 14.5
DART-MS m/z calcd for $C_{15}H_{15}O_2^+$ $[M + H]^+$ = 227.1067, found 227.1078

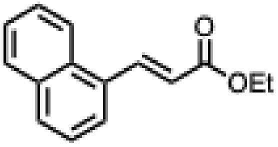

Compound (31): ethyl (*E*)-3-(naphthalen-1-yl)acrylate
Prepared according to Procedure A from 1-(bromomethyl)naphthalene (0.2 mmol, 1.0 equiv), diethyl maleate (0.6 mmol, 3.0 equiv) in dry $CHCl_3$ (0.5 M) over 24 h to afford the title compound as a colorless oil (31 mg, 69% yield). FCC solvent: 5% EtOAc/hexanes

^{1}H NMR (500 MHz, $CDCl_3$) δ 8.54 (d, J = 15.8 Hz, 1H), 8.23 – 8.18 (m, 1H), 7.92 – 7.85 (m, 2H), 7.76 (dt, J = 7.2, 0.9 Hz, 1H), 7.61 – 7.46 (m, 3H), 6.54 (d, J = 15.7 Hz, 1H), 4.33 (q, J = 7.1 Hz, 2H), 1.39 (t, J = 7.1 Hz, 3H)
^{13}C NMR (126 MHz, $CDCl_3$) δ 167.0, 141.7, 133.8, 131.9, 131.5, 130.6, 128.8, 127.0, 126.3, 125.6, 125.1, 123.5, 121.1, 60.7, 14.5
DART-MS m/z calcd for $C_{15}H_{15}O_2^+$ $[M + H]^+$ = 227.1067, found 227.1078

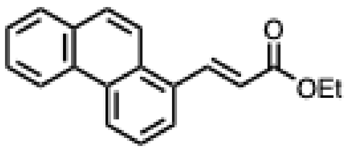

Compound (32): ethyl (*E*)-3-(phenanthren-1-yl)acrylate
Prepared according to Procedure A from 1-(bromomethyl)phenanthrene (0.2 mmol, 1.0 equiv), diethyl maleate (0.6 mmol, 3.0 equiv) in dry $CHCl_3$ (0.5 M) over 24 h to afford the title compound as a colorless oil (44 mg, 80% yield). FCC solvent: 5% EtOAc/hexanes

^{1}H NMR (500 MHz, $CDCl_3$) δ 8.74 (dd, *J* = 7.9, 1.6 Hz, 1H), 8.67 (d, *J* = 8.2 Hz, 1H), 8.53 (dd, *J* = 15.7, 0.8 Hz, 1H), 8.22 (dd, *J* = 7.8, 1.7 Hz, 1H), 7.99 (s, 1H), 7.91 (dd, *J* = 7.9, 1.4 Hz, 1H), 7.73 – 7.66 (m, 3H), 7.62 (ddd, *J* = 8.0, 7.0, 1.2 Hz, 1H), 6.62 (d, *J* = 15.7 Hz, 1H), 4.34 (q, *J* = 7.1 Hz, 2H), 1.40 (t, *J* = 7.1 Hz, 3H)
^{13}C NMR (126 MHz, $CDCl_3$) δ 167.0, 142.5, 131.3, 131.2, 131.2, 130.6, 130.2, 129.3, 127.8, 127.2, 127.2, 127.1, 126.7, 124.5, 123.3, 122.8, 121.7, 60.8, 14.5
DART-MS m/z calcd for $C_{19}H_{17}O_2^+$ $[M + H]^+$ = 277.1223, found 277.1225

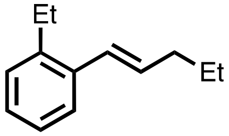

Compound (33): (*E*)-1-ethyl-2-(pent-1-en-1-yl)benzene
Prepared according to Procedure B from 2-ethylbenzyl alcohol (0.2 mmol, 1.0 equiv), *cis*-4-octene (0.6 mmol, 3.0 equiv), trifluoroacetic acid (2.0 equiv), 4Å molecular sieves (MS) in dry $CHCl_3$ (0.5 M) over 36 h to afford the title compound as a colorless oil (26 mg, 74% yield). FCC solvent: pentane

^{1}H NMR (500 MHz, $CDCl_3$) δ 7.45 – 7.39 (m, 1H), 7.20 – 7.09 (m, 3H), 6.62 (dt, *J* = 15.6, 1.5 Hz, 1H), 6.09 (dt, *J* = 15.4, 6.9 Hz, 1H), 2.69 (q, *J* = 7.6 Hz, 2H), 2.21 (qd, *J* = 7.1, 1.5 Hz, 2H), 1.50 (sext, *J* = 7.4 Hz, 2H), 1.20 (t, *J* = 7.6 Hz, 3H), 0.96 (t, *J* = 7.4 Hz, 3H)
^{13}C NMR (126 MHz, $CDCl_3$) δ 141.1, 136.6, 132.6, 128.7, 127.6, 127.1, 126.1, 125.9, 35.6, 26.5, 22.8, 15.4, 13.9
DART-MS m/z calcd for $C_{13}H_{19}O^+$ $[M + OH]^+$ = 191.1430, found 191.1440

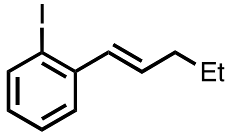

Compound (34): (*E*)-1-iodo-2-(pent-1-en-1-yl)benzene
Prepared according to Procedure B from 2-iodobenzyl alcohol (0.2 mmol, 1.0 equiv), *cis*-4-octene (0.6 mmol, 3.0 equiv), trifluoroacetic acid (2.0 equiv), 4Å molecular sieves (MS) in dry $CHCl_3$ (0.5 M) over 24 h to afford the title compound as a colorless oil (43 mg, 78% yield). FCC solvent: pentane

^{1}H NMR (500 MHz, $CDCl_3$) δ 7.81 (dd, J = 7.9, 1.3 Hz, 1H), 7.45 (dd, J = 7.8, 1.7 Hz, 1H), 7.30 – 7.26 (m, 1H), 6.89 (td, J = 7.6, 1.7 Hz, 1H), 6.56 (dt, J = 15.5, 1.6 Hz, 1H), 6.10 (dt, J = 15.5, 6.9 Hz, 1H), 2.24 (qd, J = 7.1, 1.5 Hz, 2H), 1.53 (sext, J = 7.4 Hz, 2H), 0.98 (t, J = 7.4 Hz, 3H)
^{13}C NMR (126 MHz, $CDCl_3$) δ 141.1, 139.5, 134.4, 133.9, 128.5, 128.4, 126.5, 99.6, 35.2, 22.5, 13.9
DART-MS m/z calcd for $C_{11}H_{14}IO^+$ $[M + OH]^+$ = 289.0084, found 289.0095

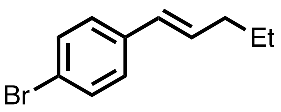

Compound (35): (*E*)-1-bromo-4-(pent-1-en-1-yl)benzene
Prepared according to Procedure B from 4-bromobenzyl alcohol (0.2 mmol, 1.0 equiv), *cis*-4-octene (0.6 mmol, 3.0 equiv), trifluoroacetic acid (2.0 equiv), 4Å molecular sieves (MS) in dry $CHCl_3$ (0.5 M) over 24 h to afford the title compound as a colorless oil (36 mg, 80% yield). FCC solvent: pentane

^{1}H NMR (500 MHz, $CDCl_3$) δ 7.43 – 7.37 (m, 2H), 7.23 – 7.17 (m, 2H), 6.32 (d, J = 16.0 Hz, 1H), 6.22 (dt, J = 15.8, 6.7 Hz, 1H), 2.23 – 2.13 (m, 2H), 1.49 (sext, J = 7.3 Hz, 2H), 0.95 (t, J = 7.4 Hz, 3H)
^{13}C NMR (126 MHz, $CDCl_3$) δ 137.0, 132.0, 131.7, 128.9, 127.6, 120.5, 35.2, 22.6, 13.9
DART-MS m/z calcd for $C_{11}H_{14}BrO^+$ $[M + OH]^+$ = 241.0223, found 241.0244

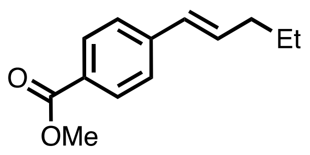

Compound (36): methyl (*E*)-4-(pent-1-en-1-yl)benzoate
Prepared according to Procedure B from methyl 4-(hydroxymethyl)benzoate (0.2 mmol, 1.0 equiv), *cis*-4-octene (0.6 mmol, 3.0 equiv), trifluoroacetic acid (2.0 equiv), 4Å molecular sieves (MS) in dry $CHCl_3$ (0.5 M) over 24 h to afford the title compound as a white solid (32 mg, 77% yield). FCC solvent: 5% EtOAc/hexanes

^{1}H NMR (500 MHz, $CDCl_3$) δ 7.99 – 7.93 (m, 2H), 7.42 – 7.35 (m, 2H), 6.42 (d, J = 16.0 Hz, 1H), 6.36 (dt, J = 15.8, 6.3 Hz, 1H), 3.90 (s, 3H), 2.26 – 2.18 (m, 2H), 1.51 (sext, J = 7.4 Hz, 2H), 0.96 (t, J = 7.3 Hz, 3H)
^{13}C NMR (126 MHz, $CDCl_3$) δ 167.2, 142.6, 134.1, 130.0, 129.3, 128.4, 125.9, 52.1, 35.3, 22.5, 13.9
DART-MS m/z calcd for $C_{13}H_{17}O_2^+$ $[M + H]^+$ = 205.1229, found 205.1240

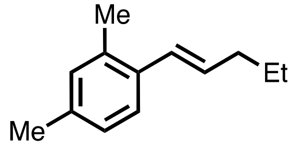

Compound (37): (*E*)-2,4-dimethyl-1-(pent-1-en-1-yl)benzene
Prepared according to Procedure B from (2,4-dimethylphenyl)methanol (0.2 mmol, 1.0 equiv), *cis*-4-octene (0.6 mmol, 3.0 equiv), trifluoroacetic acid (2.0 equiv), 4Å molecular sieves (MS) in dry $CHCl_3$ (0.5 M) over 24 h to afford the title compound as a colorless oil (29 mg, 82% yield). FCC solvent: pentane

^{1}H NMR (500 MHz, $CDCl_3$) δ 7.32 (d, J = 7.6 Hz, 1H), 6.96 (d, J = 7.8 Hz, 2H), 6.54 (dt, J = 15.5, 1.6 Hz, 1H), 6.05 (dt, J = 15.6, 7.0 Hz, 1H), 2.31 (s, 3H), 2.30 (s, 3H), 2.21 (qd, J = 7.1, 1.5 Hz, 2H), 1.51 (sext, J = 7.4 Hz, 2H), 0.97 (t, J = 7.3 Hz, 3H)
^{13}C NMR (126 MHz, $CDCl_3$) δ 136.5, 134.8, 134.4, 131.6, 131.0, 127.7, 126.9, 125.5, 35.6, 22.8, 21.2, 19.9, 13.8
DART-MS m/z calcd for $C_{13}H_{19}O^+$ $[M + OH]^+$ = 191.1430, found 191.1441

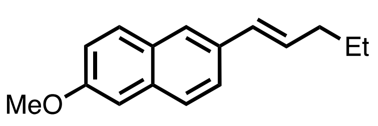

Compound (38): (*E*)-2-methoxy-6-(pent-1-en-1-yl)naphthalene
Prepared according to Procedure B from (6-methoxynaphthalen-2-yl)methanol (0.2 mmol, 1.0 equiv), *cis*-4-octene (0.6 mmol, 3.0 equiv), trifluoroacetic acid (2.0 equiv), 4Å molecular sieves (MS) in dry $CHCl_3$ (0.5 M) over 36 h to afford the title compound as a white solid (32 mg, 70% yield). FCC solvent: 5% EtOAc/hexanes

^{1}H NMR (500 MHz, $CDCl_3$) δ 7.67 (dd, *J* = 10.6, 8.6 Hz, 2H), 7.61 (d, *J* = 1.7 Hz, 1H), 7.55 (dd, *J* = 8.5, 1.8 Hz, 1H), 7.16 – 7.08 (m, 2H), 6.51 (dt, *J* = 15.9, 1.6 Hz, 1H), 6.30 (dt, *J* = 15.8, 7.0 Hz, 1H), 3.91 (s, 3H), 2.23 (qd, *J* = 7.1, 1.5 Hz, 2H), 1.53 (sext, J = 7.6 Hz, 2H), 0.98 (t, *J* = 7.4 Hz, 3H)
^{13}C NMR (126 MHz, $CDCl_3$) δ 157.6, 133.9, 133.5, 130.6, 130.1, 129.5, 129.3, 127.0, 125.3, 124.3, 119.0, 106.0, 55.4, 35.4, 22.8, 13.9
DART-MS m/z calcd for $C_{16}H_{19}O_2^+$ $[M + OH]^+$ = 243.1380, found 243.1391

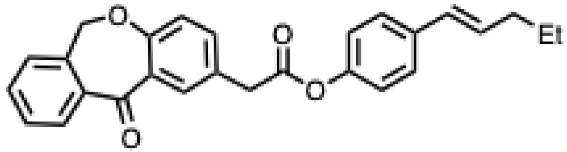

Compound (39):
(*E*)-4-(pent-1-en-1-yl)phenyl 2-(11-oxo-6,11-dihydrodibenzo[*b*,*e*]oxepin-2-yl)acetate
Prepared according to Procedure B from 4-(hydroxymethyl)phenyl 2-(11-oxo-6,11-dihydrodibenzo[*b,e*]oxepin-2-yl)acetate (0.2 mmol, 1.0 equiv), *cis*-4-octene (0.6 mmol, 3.0 equiv), trifluoroacetic acid (2.0 equiv), 4Å molecular sieves (MS) in dry $CHCl_3$ (0.5 M) over 36 h to afford the title compound as an oil (56 mg, 68% yield). FCC solvent: 5% EtOAc/hexanes

^{1}H NMR (500 MHz, $CDCl_3$) δ 8.23 (d, *J* = 2.4 Hz, 1H), 7.91 (dd, *J* = 7.7, 1.4 Hz, 1H), 7.59 – 7.50 (m, 2H), 7.48 (td, *J* = 7.6, 1.3 Hz, 1H), 7.39 – 7.35 (m, 1H), 7.34 – 7.28 (m, 2H), 7.07 (d, *J* = 8.4 Hz, 1H), 7.04 – 6.94 (m, 2H), 6.35 (dt, *J* = 15.8, 1.5 Hz, 1H), 6.17 (dt, *J* = 15.8, 7.0 Hz, 1H), 5.20 (s, 2H), 3.88 (s, 2H), 2.18 (qd, *J* = 7.1, 1.5 Hz, 2H), 1.49 (h, *J* = 7.4 Hz, 2H), 0.94 (t, *J* = 7.4 Hz, 3H)
^{13}C NMR (126 MHz, $CDCl_3$) δ 190.9, 170.0, 160.7, 149.5, 140.5, 136.4, 135.9, 135.6, 132.9, 132.7, 131.4, 129.6, 129.4, 129.0, 128.0, 127.4, 126.9, 125.3, 121.5, 121.4, 73.7, 40.4, 35.2, 22.6, 13.8
DART-MS m/z calcd for $C_{27}H_{25}O_4^+$ $[M + H]^+$ = 413.1747, found 413.1758

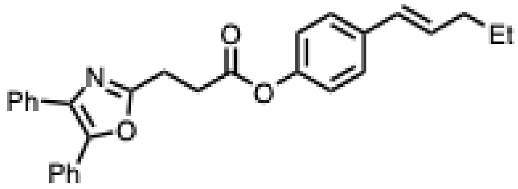

Compound (40): (*E*)-4-(pent-1-en-1-yl)phenyl 3-(4,5-diphenyloxazol-2-yl)propanoate

Prepared according to Procedure B from 4-(hydroxymethyl)phenyl 2-(11-oxo-6,11-dihydrodibenzo[*b,e*]oxepin-2-yl)acetate (0.2 mmol, 1.0 equiv), *cis*-4-octene (0.6 mmol, 3.0 equiv), trifluoroacetic acid (2.0 equiv), 4Å molecular sieves (MS) in dry $CHCl_3$ (0.5 M) over 36 h to afford the title compound as a colorless oil (70 mg, 80% yield). FCC solvent: 10% EtOAc/hexanes

^{1}H NMR (500 MHz, $CDCl_3$) δ 7.69 – 7.63 (m, 2H), 7.62 – 7.56 (m, 2H), 7.42 – 7.29 (m, 8H), 7.07 – 7.00 (m, 2H), 6.36 (dt, *J* = 15.8, 1.5 Hz, 1H), 6.18 (dt, *J* = 15.8, 6.9 Hz, 1H), 3.31 (t, *J* = 7.6 Hz, 2H), 3.16 (t, *J* = 7.3 Hz, 2H), 2.18 (qd, *J* = 7.1, 1.4 Hz, 2H), 1.50 (h, *J* = 7.4 Hz, 2H), 0.95 (t, *J* = 7.4 Hz, 3H)
^{13}C NMR (126 MHz, $CDCl_3$) δ 170.8, 161.6, 149.5, 145.7, 136.0, 135.3, 132.6, 131.4, 129.1, 129.0, 128.8, 128.7, 128.6, 128.2, 128.0, 126.9, 126.7, 121.6, 35.2, 31.4, 23.7, 22.6, 13.9
DART-MS m/z calcd for $C_{29}H_{28}NO_3^+$ $[M + H]^+$ = 438.2064, found 438.2066

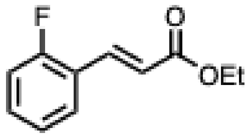

Compound (41): ethyl (*E*)-3-(2-fluorophenyl)acrylate

Prepared according to Procedure B from 2-fluorobenzyl alcohol (0.2 mmol, 1.0 equiv), diethyl maleate (0.6 mmol, 3.0 equiv), trifluoroacetic acid (2.0 equiv), 4Å molecular sieves (MS) in dry $CHCl_3$ (0.5 M) over 24 h to afford the title compound as a colorless oil (30 mg, 78% yield). FCC solvent: 5% EtOAc/hexanes

^{1}H NMR (500 MHz, $CDCl_3$) δ 7.81 (d, *J* = 16.2 Hz, 1H), 7.53 (td, *J* = 7.6, 1.6 Hz, 1H), 7.34 (dddd, *J* = 8.3, 6.9, 4.2, 1.6 Hz, 1H), 7.15 (td, *J* = 7.5, 1.4 Hz, 1H), 7.09 (ddt, *J* = 10.8, 8.3, 1.3 Hz, 1H), 6.53 (dd, *J* = 16.2, 1.0 Hz, 1H), 4.27 (qd, *J* = 7.1, 0.9 Hz, 2H), 1.34 (td, *J* = 7.1, 1.0 Hz, 3H)
^{13}C NMR (126 MHz, $CDCl_3$) δ 137.3 (d, *J* = 2.8 Hz), 131.7 (d, *J* = 8.7 Hz), 129.2 (d, *J* = 2.9 Hz), 124.5 (d, *J* = 3.7 Hz), 122.7 (d, *J* = 11.7 Hz), 121.0 (d, *J* = 6.6 Hz), 116.3 (d, *J* = 21.9 Hz)
^{19}F NMR (470 MHz, $CDCl_3$) δ -114.40 (dt, *J* = 12.0, 6.3 Hz)
DART-MS m/z calcd for $C_{11}H_{12}FO_2^+$ $[M + H]^+$ = 195.0816, found 195.0818

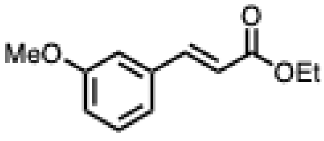

Compound (42): ethyl (*E*)-3-(3-methoxyphenyl)acrylate

Prepared according to Procedure B from (3-methoxyphenyl)methanol (0.2 mmol, 1.0 equiv), diethyl maleate (0.6 mmol, 3.0 equiv), trifluoroacetic acid (2.0 equiv), 4Å molecular sieves (MS) in dry $CHCl_3$ (0.5 M) over 36 h to afford the title compound as a colorless oil (31 mg, 75% yield). FCC solvent: 10% EtOAc/hexanes

^{1}H NMR (500 MHz, $CDCl_3$) δ 7.65 (d, *J* = 15.9 Hz, 1H), 7.29 (t, *J* = 7.9 Hz, 1H), 7.12 (dt, *J* = 7.5, 1.2 Hz, 1H), 7.04 (t, *J* = 2.2 Hz, 1H), 6.93 (ddd, *J* = 8.2, 2.7, 0.9 Hz, 1H), 6.42 (d, *J* = 16.0 Hz, 1H), 4.26 (q, *J* = 7.2 Hz, 2H), 3.83 (s, 3H), 1.34 (t, *J* = 7.1 Hz, 3H)
^{13}C NMR (126 MHz, $CDCl_3$) δ 167.1, 160.0, 144.6, 136.0, 130.0, 120.9, 118.7, 116.2, 113.0, 60.7, 55.4, 14.5
DART-MS m/z calcd for $C_{12}H_{15}O_3^+$ $[M + H]^+$ = 207.1016, found 207.1026

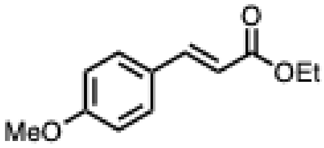

Compound (43): ethyl (*E*)-3-(4-methoxyphenyl)acrylate

Prepared according to Procedure B from (4-methoxyphenyl)methanol (0.2 mmol, 1.0 equiv), diethyl maleate (0.6 mmol, 3.0 equiv), trifluoroacetic acid (2.0 equiv), 4Å molecular sieves (MS) in dry $CHCl_3$ (0.5 M) over 36 h to afford the title compound as a colorless oil (35 mg, 84% yield). FCC solvent: 10% EtOAc/hexanes

^{1}H NMR (500 MHz, $CDCl_3$) δ 7.64 (d, *J* = 16.0 Hz, 1H), 7.50 – 7.44 (m, 2H), 6.93 – 6.87 (m, 2H), 6.30 (d, *J* = 16.0 Hz, 1H), 4.25 (q, *J* = 7.1 Hz, 2H), 3.83 (s, 3H), 1.33 (t, *J* = 7.1 Hz, 3H)
^{13}C NMR (126 MHz, $CDCl_3$) δ 167.5, 161.5, 144.4, 129.8, 127.3, 115.9, 114.4, 60.5, 55.5, 14.5
DART-MS m/z calcd for $C_{12}H_{15}O_3^+$ $[M + H]^+$ = 207.1016, found 207.1027

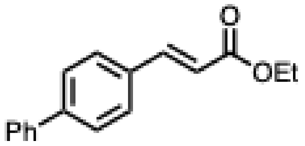

Compound (44): ethyl (*E*)-3-([1,1'-biphenyl]-4-yl)acrylate

Prepared according to Procedure B from [1,1'-biphenyl]-4-ylmethanol (0.2 mmol, 1.0 equiv), diethyl maleate (0.6 mmol, 3.0 equiv), trifluoroacetic acid (2.0 equiv), 4Å molecular sieves (MS) in dry $CHCl_3$ (0.5 M) over 24 h to afford the title compound as a white solid (34 mg, 68% yield). FCC solvent: 5% EtOAc/hexanes

^{1}H NMR (500 MHz, $CDCl_3$) δ 7.73 (d, *J* = 16.0 Hz, 1H), 7.66 – 7.58 (m, 6H), 7.46 (dd, *J* = 8.4, 7.0 Hz, 2H), 7.41 – 7.34 (m, 1H), 6.48 (d, *J* = 16.0 Hz, 1H), 4.28 (q, *J* = 7.1 Hz, 2H), 1.36 (t, *J* = 7.1 Hz, 3H)
^{13}C NMR (126 MHz, $CDCl_3$) δ 167.2, 144.3, 143.1, 140.3, 133.6, 129.0, 128.7, 128.0, 127.7, 127.2, 118.3, 60.7, 14.5
DART-MS m/z calcd for $C_{17}H_{17}O_2^+$ $[M + H]^+$ = 253.1223, found 253.1225

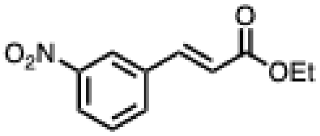

Compound (45): ethyl (*E*)-3-(3-nitrophenyl)acrylate

Prepared according to Procedure B from (3-nitrophenyl)methanol (0.2 mmol, 1.0 equiv), diethyl maleate (0.6 mmol, 3.0 equiv), trifluoroacetic acid (2.0 equiv), 4Å molecular sieves (MS) in dry $CHCl_3$ (0.5 M) over 24 h to afford the title compound as an oil (32 mg, 72% yield). FCC solvent: 10% EtOAc/hexanes

^{1}H NMR (500 MHz, $CDCl_3$) δ 8.38 (t, *J* = 2.0 Hz, 1H), 8.23 (ddd, *J* = 8.1, 2.2, 1.0 Hz, 1H), 7.82 (dt, *J* = 7.8, 1.4 Hz, 1H), 7.72 (d, *J* = 16.0 Hz, 1H), 7.58 (t, *J* = 8.0 Hz, 1H), 6.56 (d, *J* = 16.0 Hz, 1H), 4.29 (q, *J* = 7.1 Hz, 2H), 1.35 (t, *J* = 7.1 Hz, 3H)
^{13}C NMR (126 MHz, $CDCl_3$) δ 166.3, 148.9, 141.8, 136.4, 133.8, 130.1, 124.6, 122.6, 121.6, 61.1, 14.4
DART-MS m/z calcd for $C_{11}H_{12}NO_4^+$ $[M + H]^+$ = 222.0761, found 222.0781

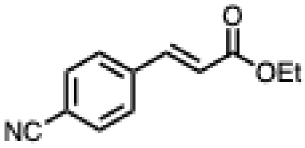

Compound (46): ethyl (*E*)-3-(4-cyanophenyl)acrylate

Prepared according to Procedure B from 4-(hydroxymethyl)benzonitrile (0.2 mmol, 1.0 equiv), diethyl maleate (0.6 mmol, 3.0 equiv), trifluoroacetic acid (2.0 equiv), 4Å molecular sieves (MS) in dry $CHCl_3$ (0.5 M) over 24 h to afford the title compound as a colorless oil (29 mg, 73% yield). FCC solvent: 10% EtOAc/hexanes

^{1}H NMR (500 MHz, $CDCl_3$) δ 7.70 – 7.63 (m, 3H), 7.61 (d, *J* = 8.3 Hz, 2H), 6.51 (d, *J* = 16.0 Hz, 1H), 4.28 (q, *J* = 7.2 Hz, 2H), 1.35 (t, *J* = 7.1 Hz, 3H)
^{13}C NMR (126 MHz, $CDCl_3$) δ 166.3, 142.3, 138.9, 132.8, 128.5, 122.0, 118.5, 113.5, 61.1, 14.4
DART-MS m/z calcd for $C_{12}H_{12}NO_2^+$ $[M + H]^+$ = 202.0863, found 202.0865

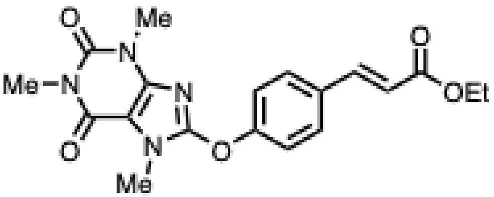

Compound (47): ethyl (*E*)-3-(4-((1,3,7-trimethyl-2,6-dioxo-2,3,6,7-tetrahydro-1*H*-purin-8-yl)oxy)phenyl)acrylate

Prepared according to Procedure B from 8-(4-(hydroxymethyl)phenoxy)-1,3,7-trimethyl-3,7-dihydro-1*H*-purine-2,6-dione (0.2 mmol, 1.0 equiv), diethyl maleate (0.6 mmol, 3.0 equiv), trifluoroacetic acid (2.0 equiv), 4Å molecular sieves (MS) in dry $CHCl_3$ (0.5 M) over 24 h to afford the title compound as a white solid (46 mg, 60% yield). FCC solvent: 60% EtOAc/hexanes

^{1}H NMR (500 MHz, $CDCl_3$) δ 7.67 (d, *J* = 16.0 Hz, 1H), 7.60 – 7.53 (m, 2H), 7.36 – 7.30 (m, 2H), 6.40 (d, *J* = 16.0 Hz, 1H), 4.26 (q, *J* = 7.1 Hz, 2H), 3.87 (s, 3H), 3.45 (s, 3H), 3.39 (s, 3H), 1.33 (t, *J* = 7.1 Hz, 3H)
^{13}C NMR (126 MHz, $CDCl_3$) δ 166.9, 155.1, 154.7, 152.9, 151.7, 145.8, 143.2, 143.2, 132.1, 129.6, 129.6, 119.8, 118.8, 104.0, 60.7, 30.6, 30.0, 28.0, 14.4
DART-MS m/z calcd for $C_{19}H_{21}N_4O_5^+$ $[M + H]^+$ = 385.1506, found 385.1516

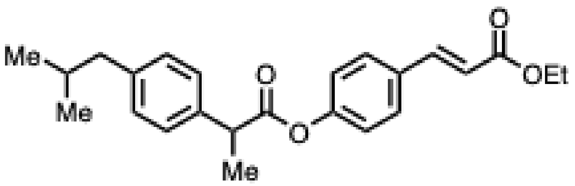

Compound (48): ethyl (*E*)-3-(4-((2-(4-isobutylphenyl)propanoyl)oxy)phenyl)acrylate
Prepared according to Procedure B from 4-(hydroxymethyl)phenyl 2-(4-isobutylphenyl)propanoate (0.2 mmol, 1.0 equiv), diethyl maleate (0.6 mmol, 3.0 equiv), trifluoroacetic acid (2.0 equiv), 4Å molecular sieves (MS) in dry $CHCl_3$ (0.5 M) over 24 h to afford the title compound as a colorless oil (51 mg, 67% yield). FCC solvent: 20% EtOAc/hexanes

^{1}H NMR (500 MHz, $CDCl_3$) δ 7.64 (d, J = 16.0 Hz, 1H), 7.52 – 7.47 (m, 2H), 7.32 – 7.27 (m, 2H), 7.17 – 7.13 (m, 2H), 7.05 – 7.01 (m, 2H), 6.37 (d, J = 16.0 Hz, 1H), 4.26 (q, J = 7.1 Hz, 2H), 3.94 (q, J = 7.1 Hz, 1H), 2.48 (d, J = 7.2 Hz, 2H), 1.87 (dp, J = 13.6, 6.8 Hz, 1H), 1.61 (d, J = 7.1 Hz, 3H), 1.33 (t, J = 7.1 Hz, 3H), 0.92 (d, J = 6.6 Hz, 6H)
^{13}C NMR (126 MHz, $CDCl_3$) δ 173.1, 167.0, 152.4, 143.6, 141.1, 137.1, 132.2, 129.7, 129.2, 127.3, 122.1, 118.5, 60.7, 45.4, 45.2, 30.3, 22.5, 18.6, 14.4
DART-MS m/z calcd for $C_{24}H_{29}O_4^+$ $[M + H]^+$ = 381.2060, found 381.2061

7. NMR SPECTRA

^{1}H (500 MHz, $CDCl_3$) NMR Spectrum of 17

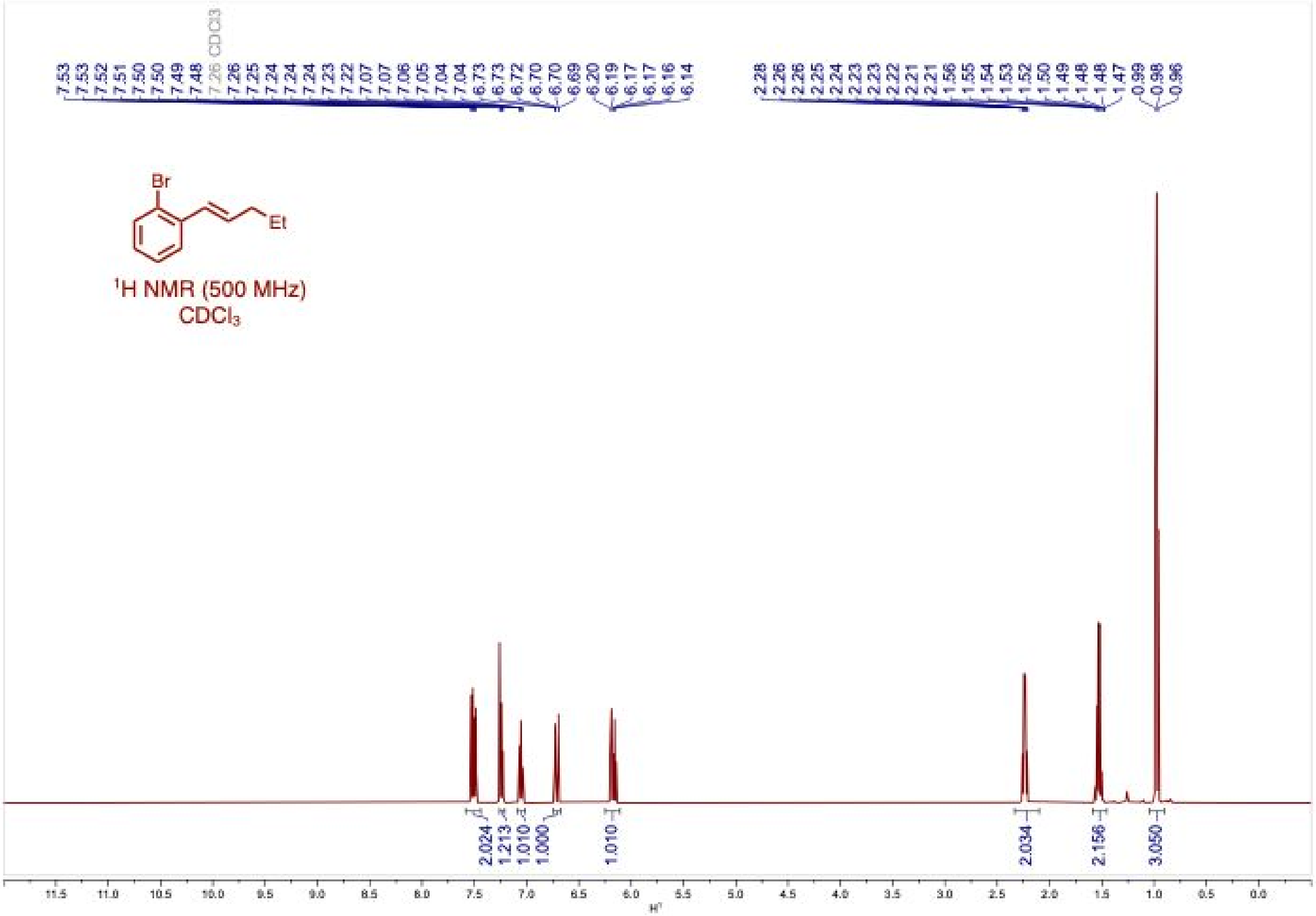

^{13}C (126 MHz, $CDCl_3$) NMR Spectrum of 17

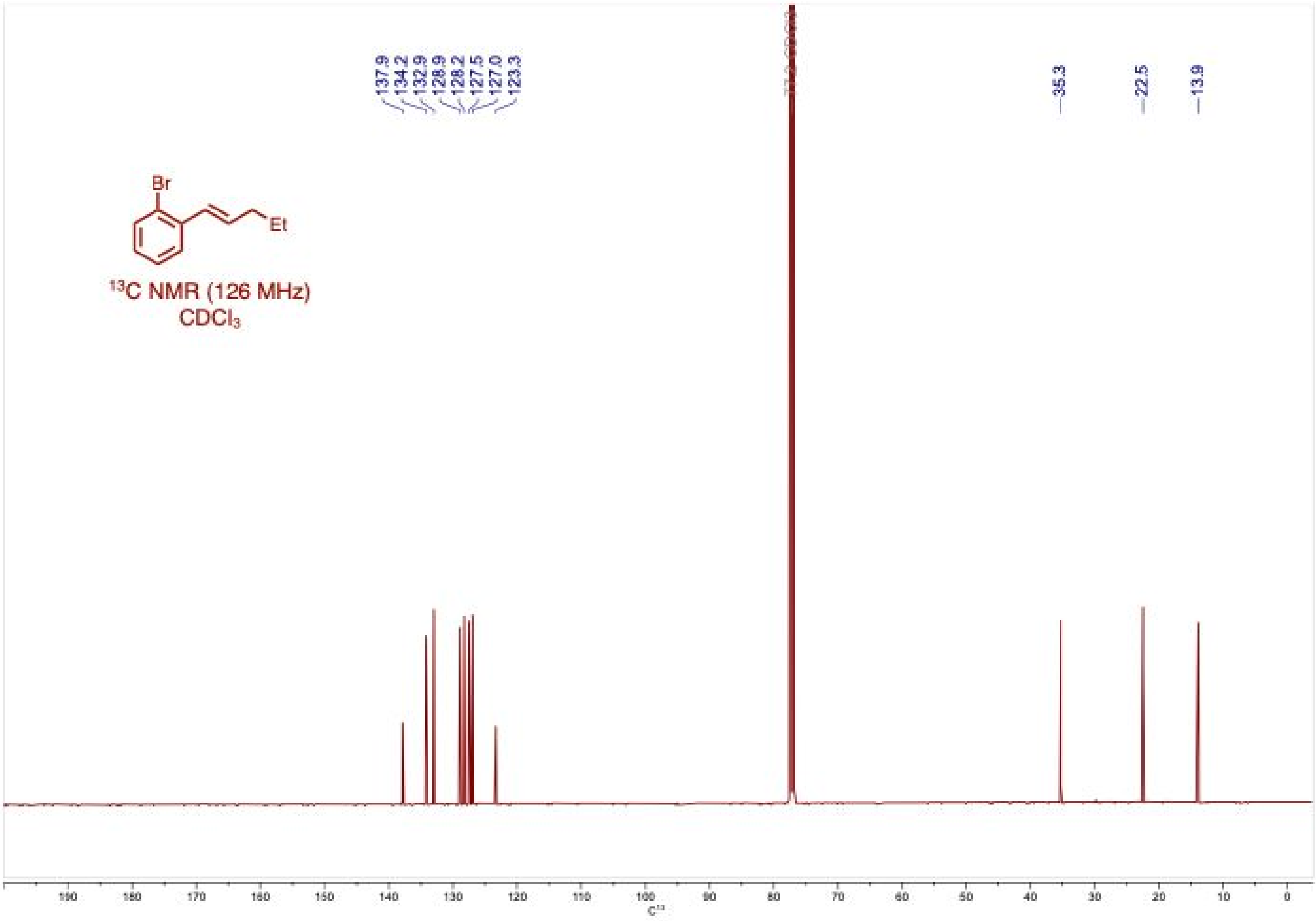

^{1}H (500 MHz, $CDCl_3$) NMR Spectrum of 19

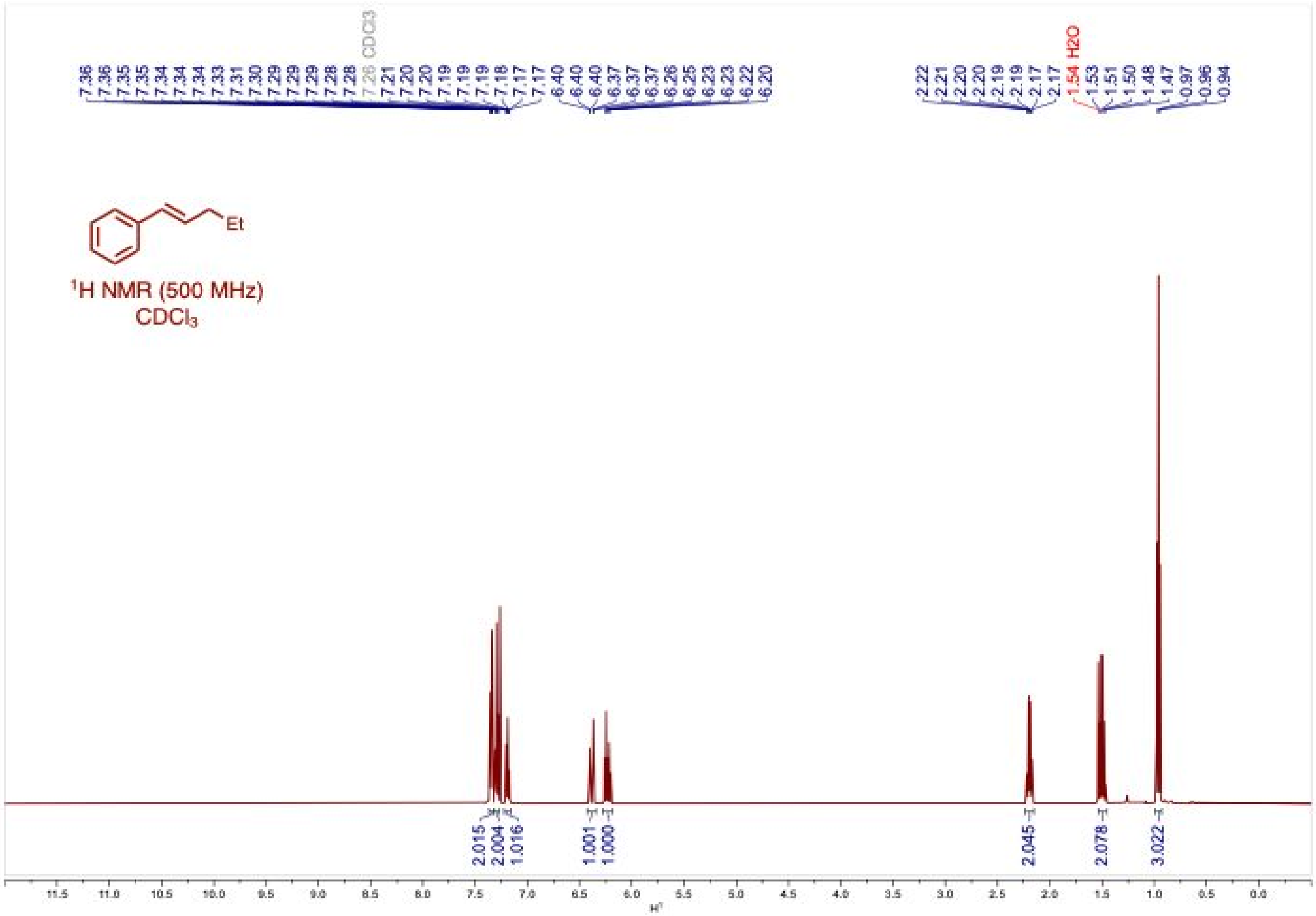

^{13}C (126 MHz, $CDCl_3$) NMR Spectrum of 19

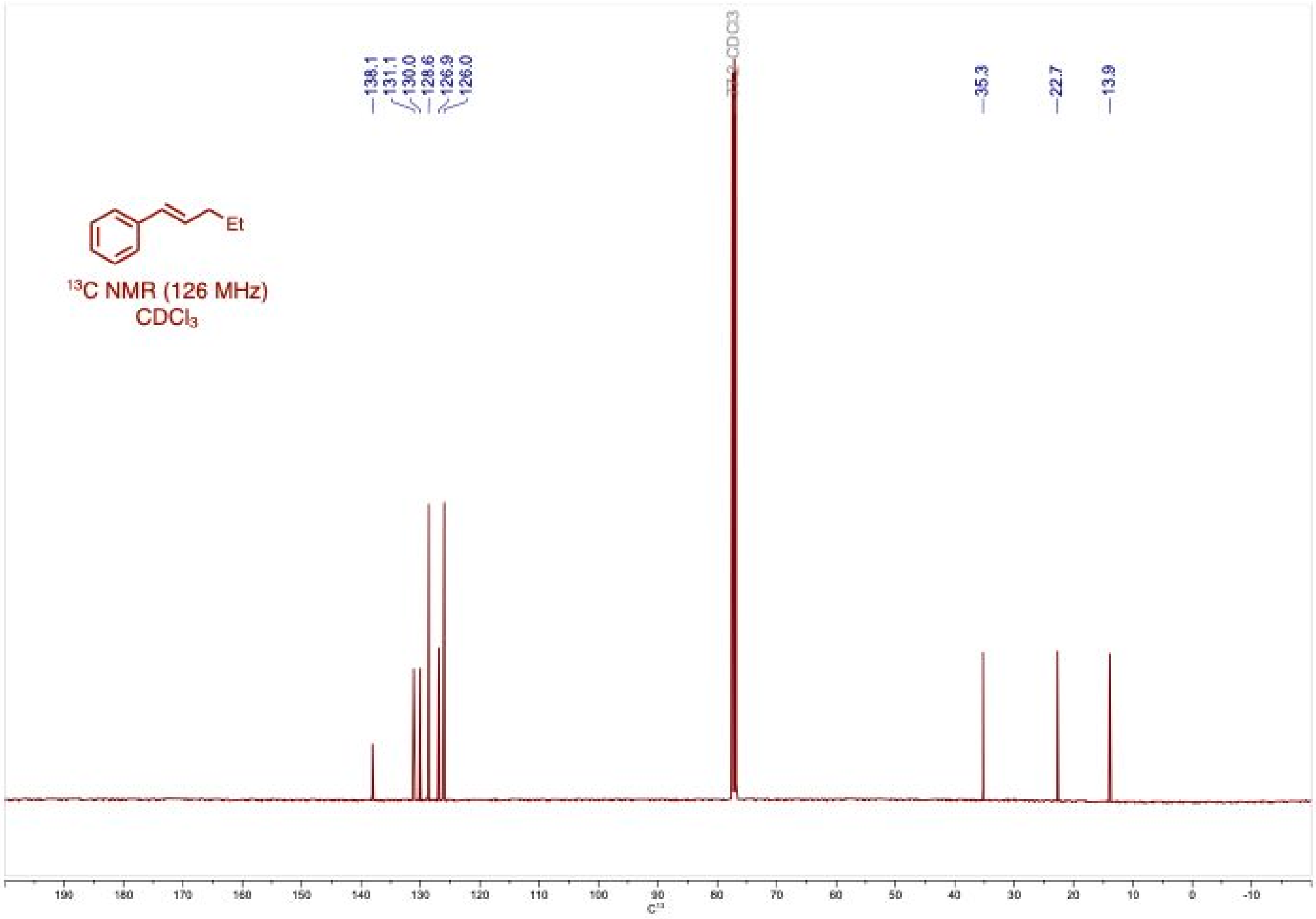

^{1}H (500 MHz, $CDCl_3$) NMR Spectrum of 20

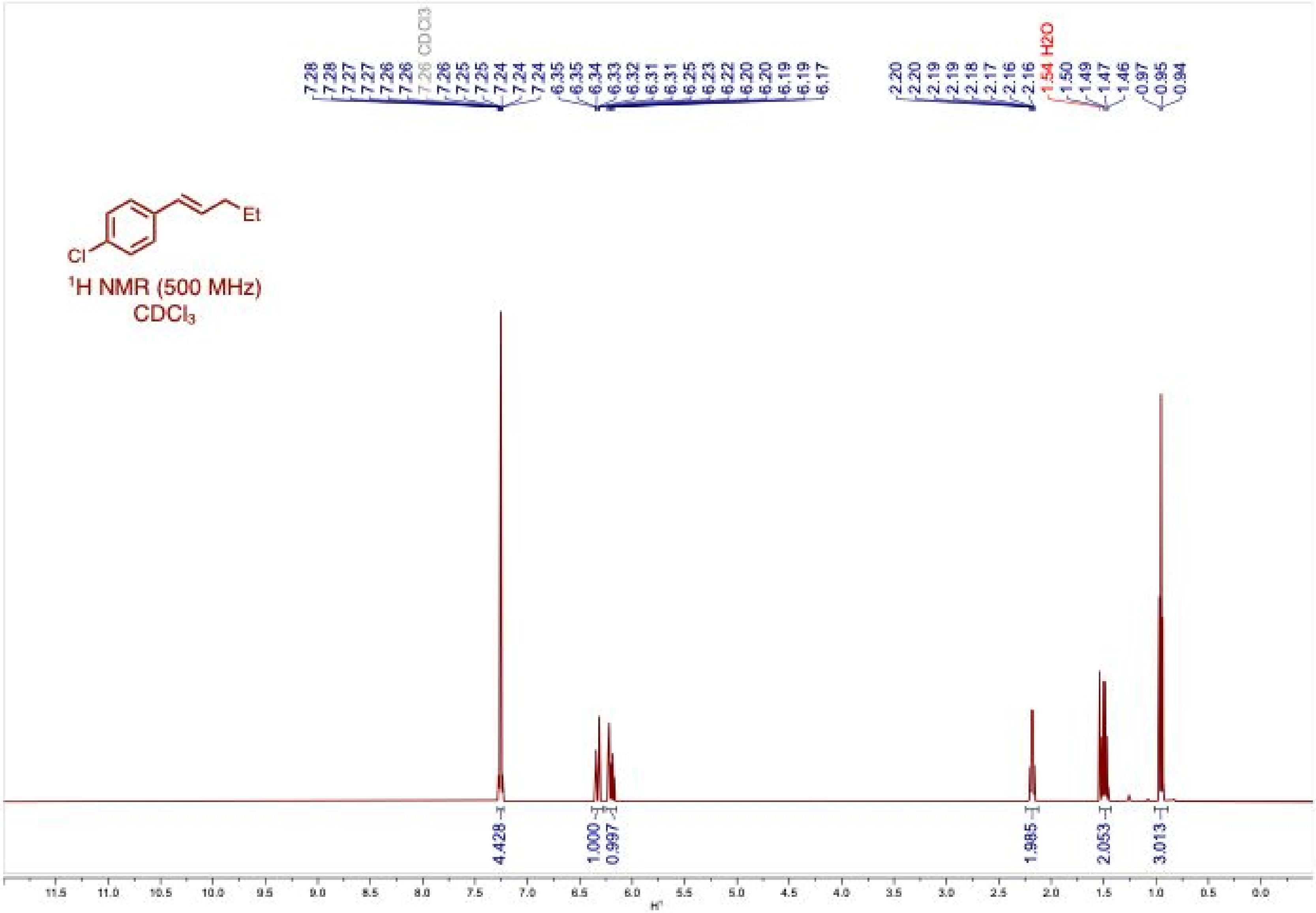

^{13}C (126 MHz, $CDCl_3$) NMR Spectrum of 20

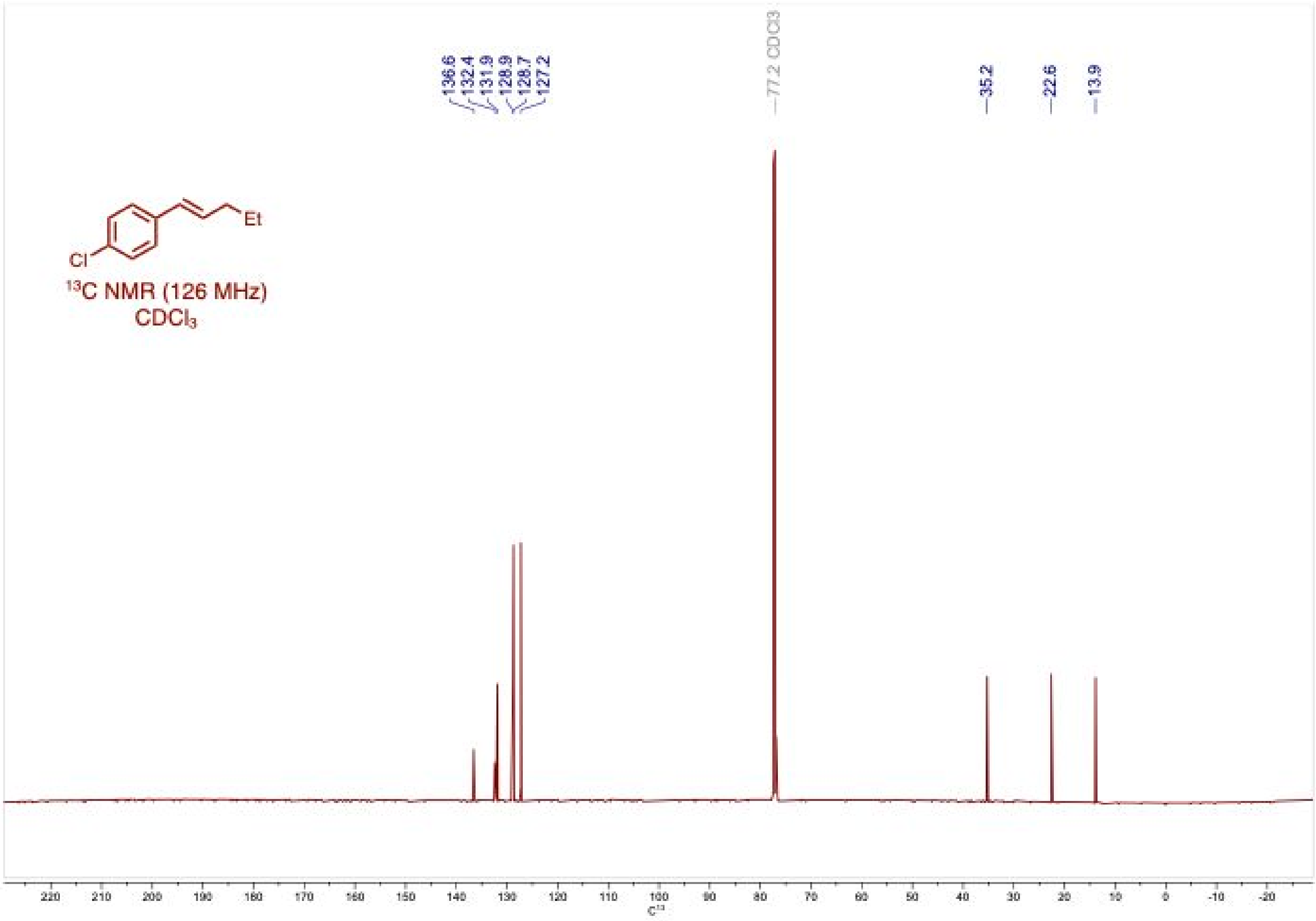

^{1}H (500 MHz, CDCl$_3$) NMR Spectrum of 21

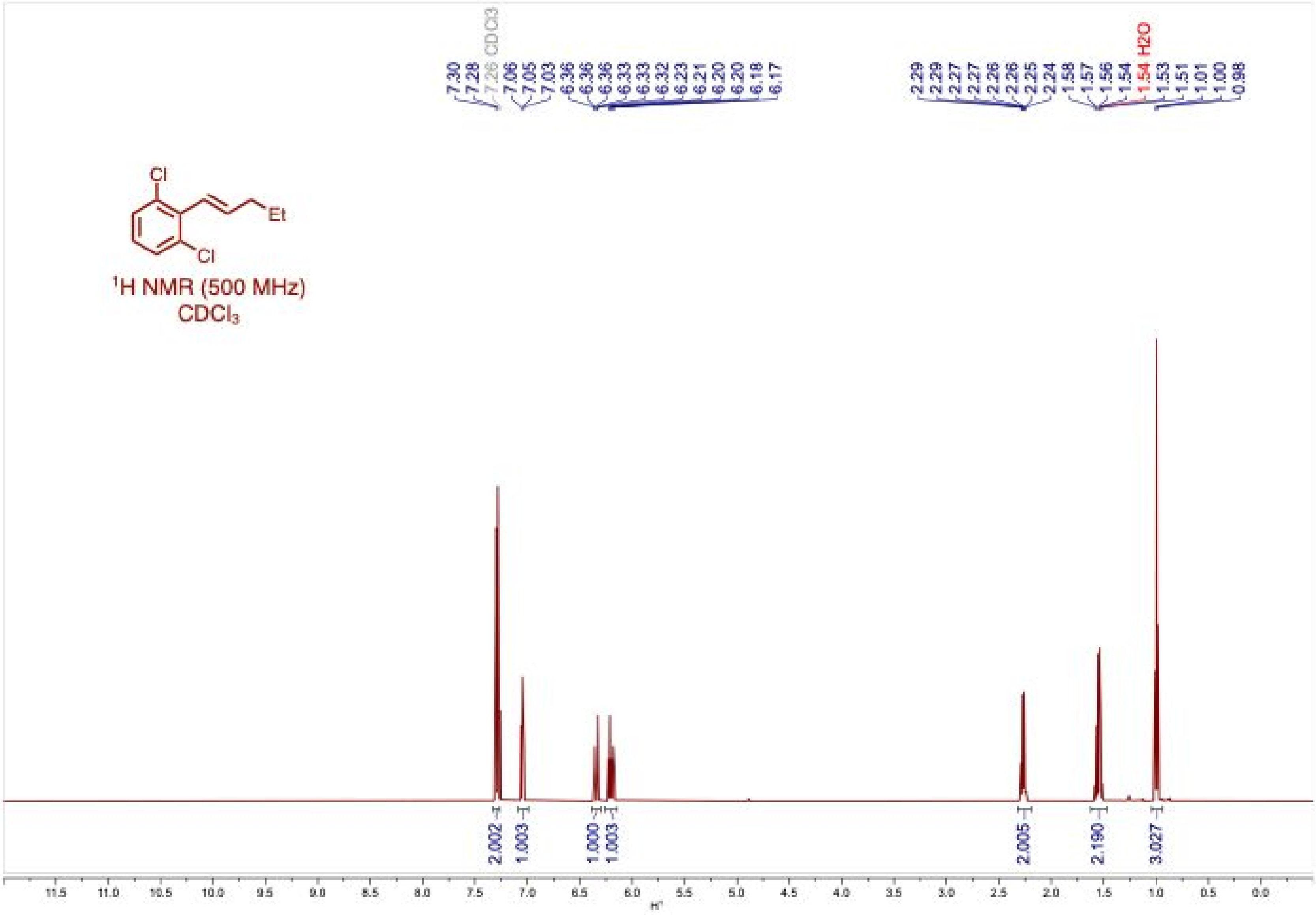

^{13}C (126 MHz, $CDCl_3$) NMR Spectrum of 21

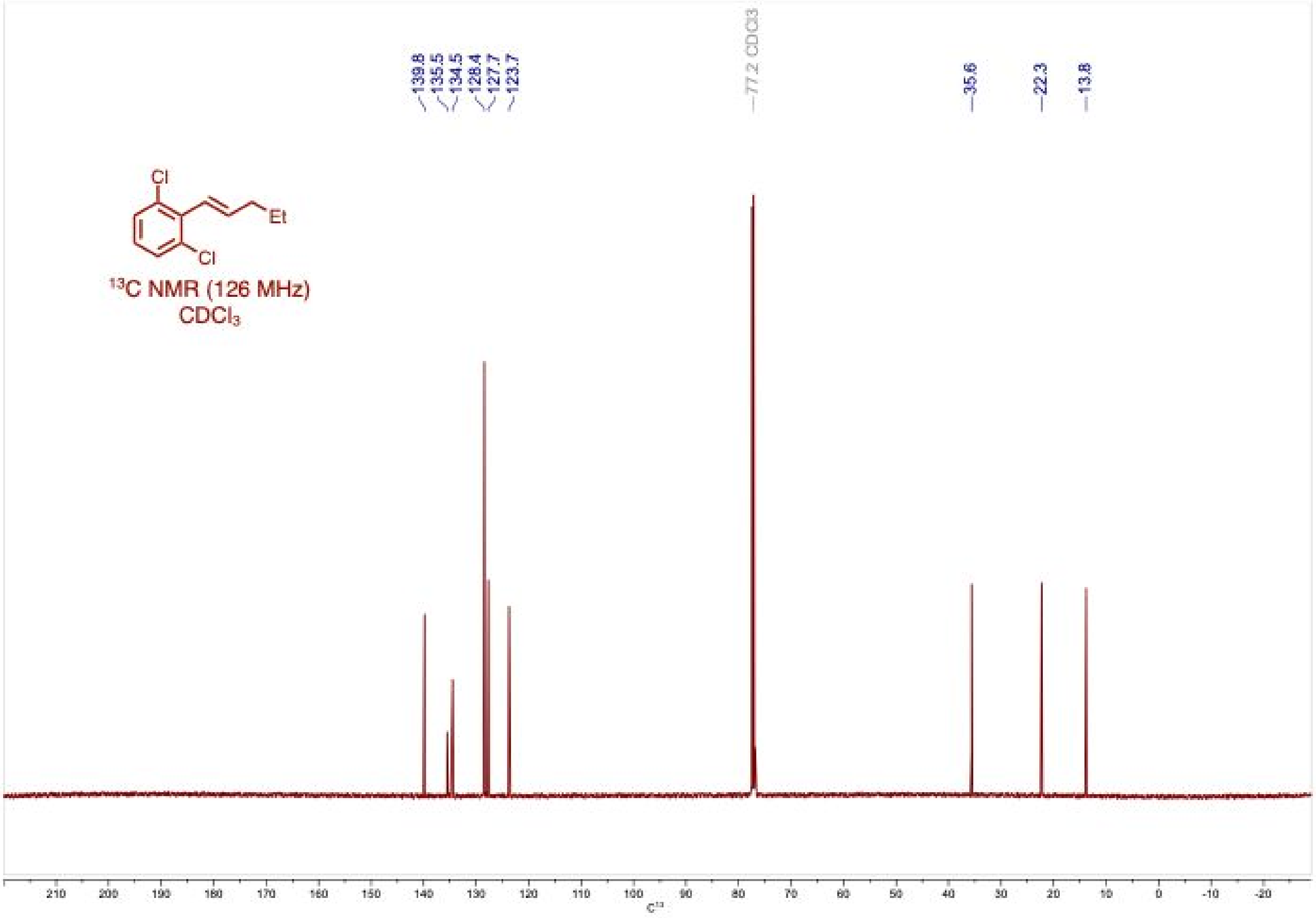

^1H (500 MHz, $CDCl_3$) NMR Spectrum of 22

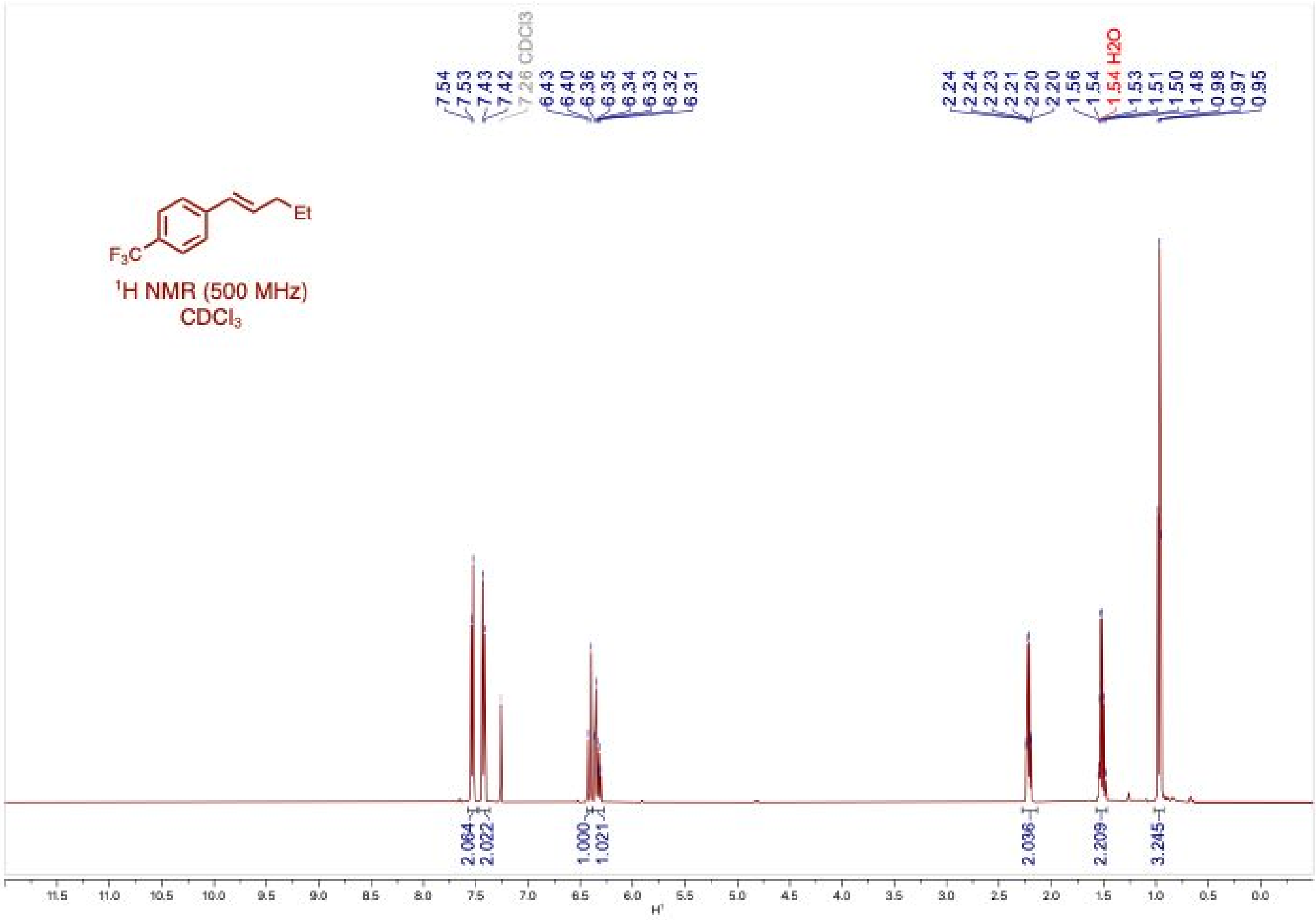

^{13}C (126 MHz, $CDCl_3$) NMR Spectrum of 22

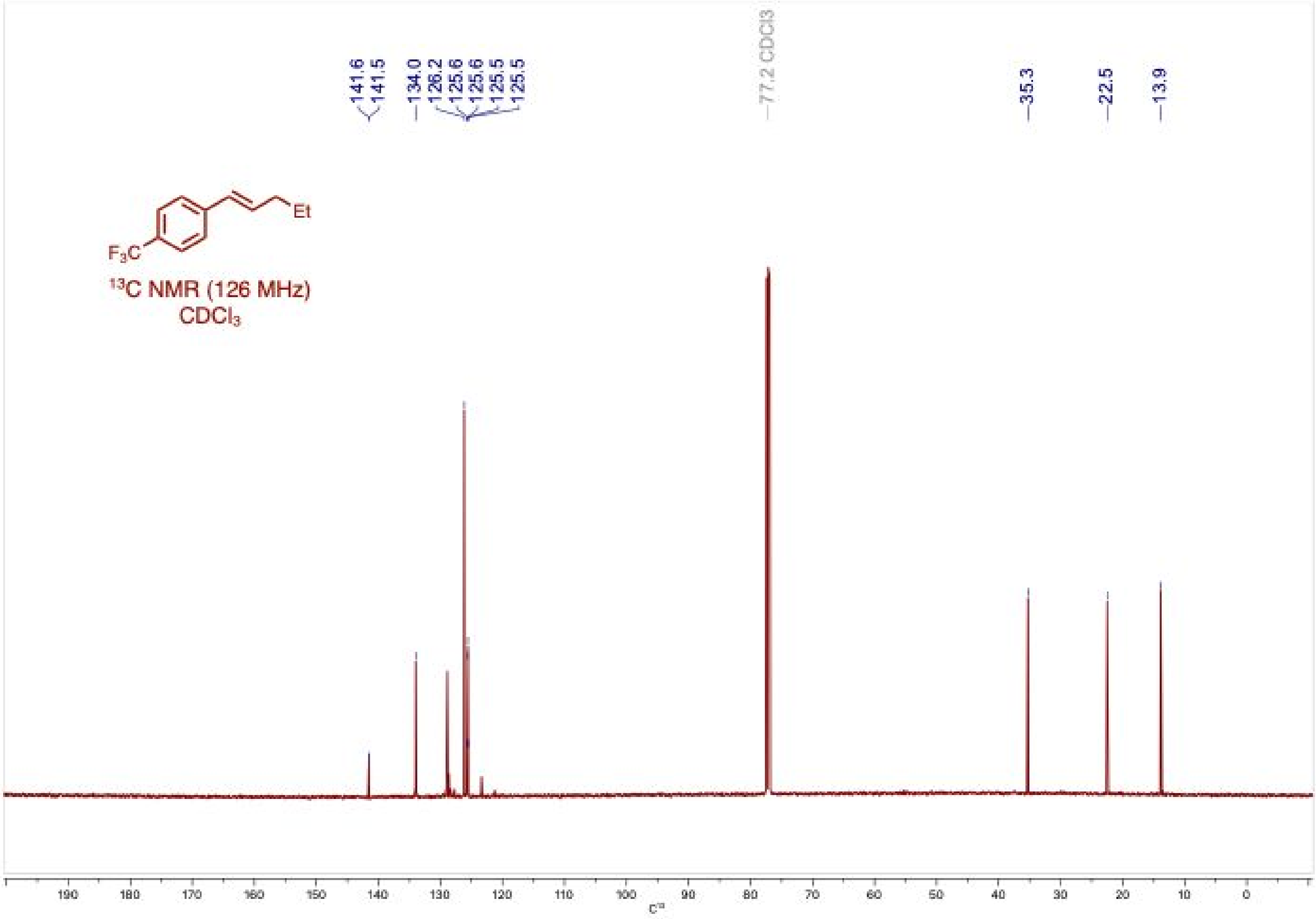

^{19}F (470 MHz, $CDCl_3$) NMR Spectrum of 22

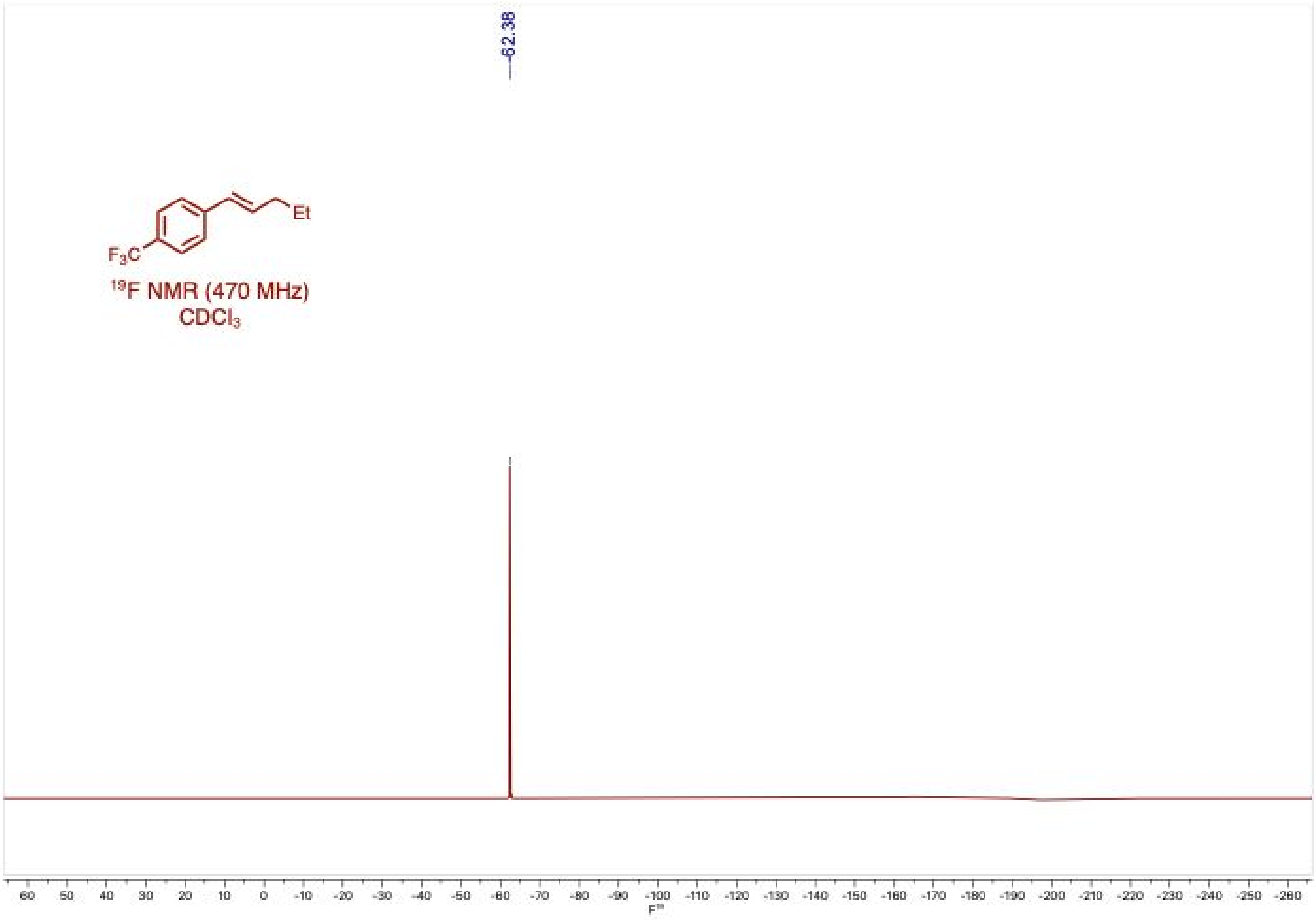

^{1}H (500 MHz, $CDCl_3$) NMR Spectrum of 23

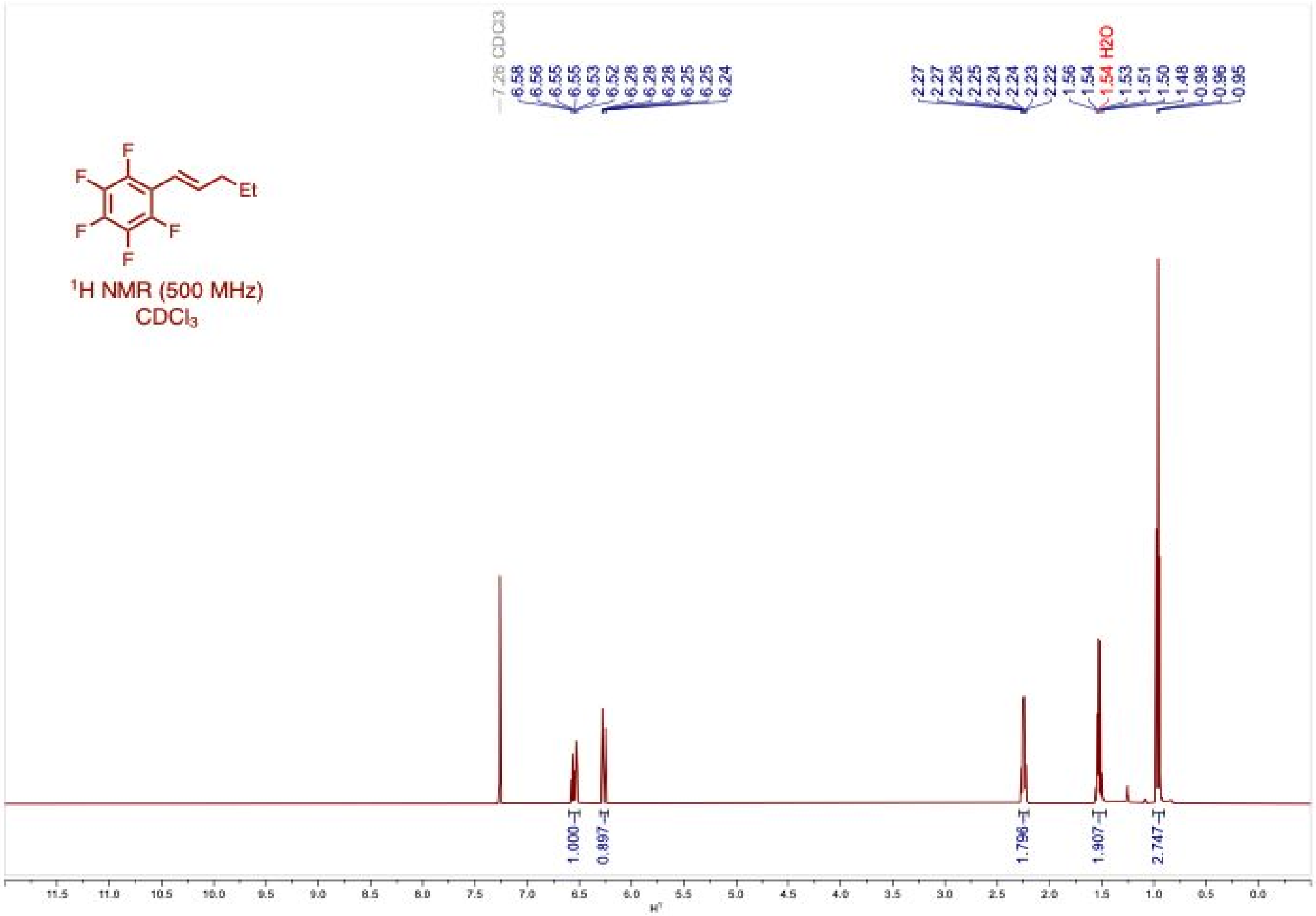

^{13}C (126 MHz, CDCl$_3$) NMR Spectrum of 23

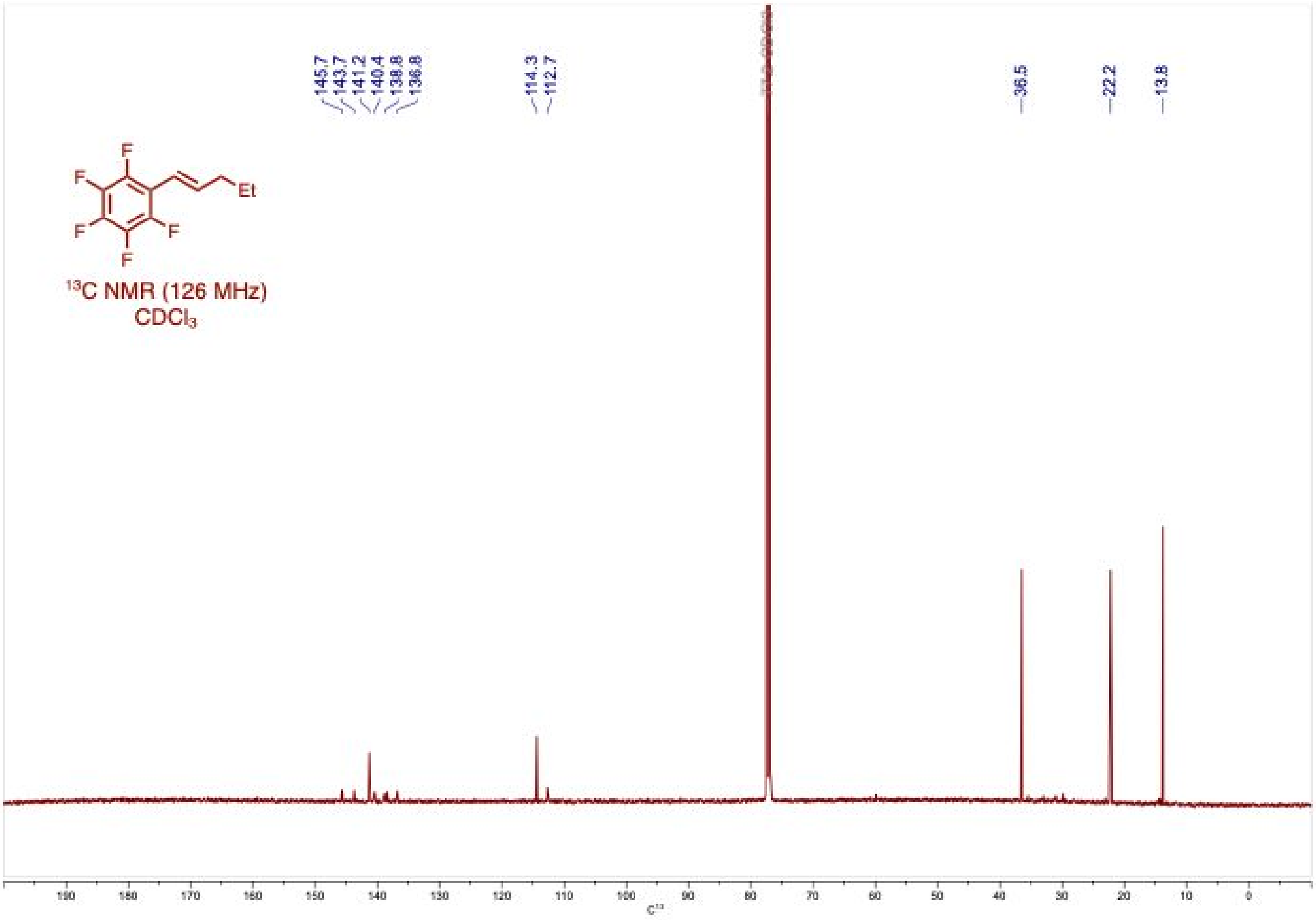

^{19}F (470 MHz, $CDCl_3$) NMR Spectrum of 23

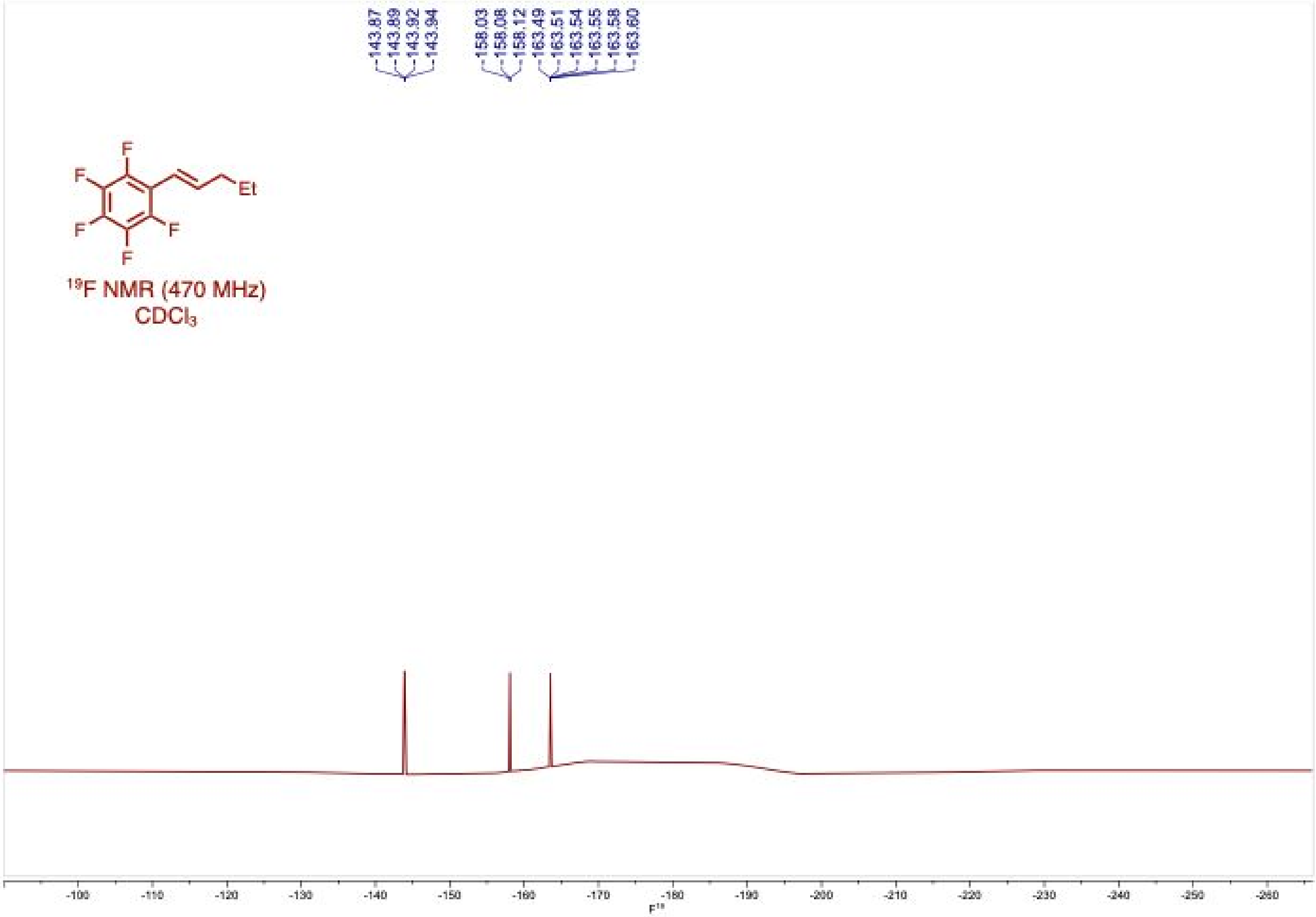

^{1}H (500 MHz, $CDCl_3$) NMR Spectrum of 25

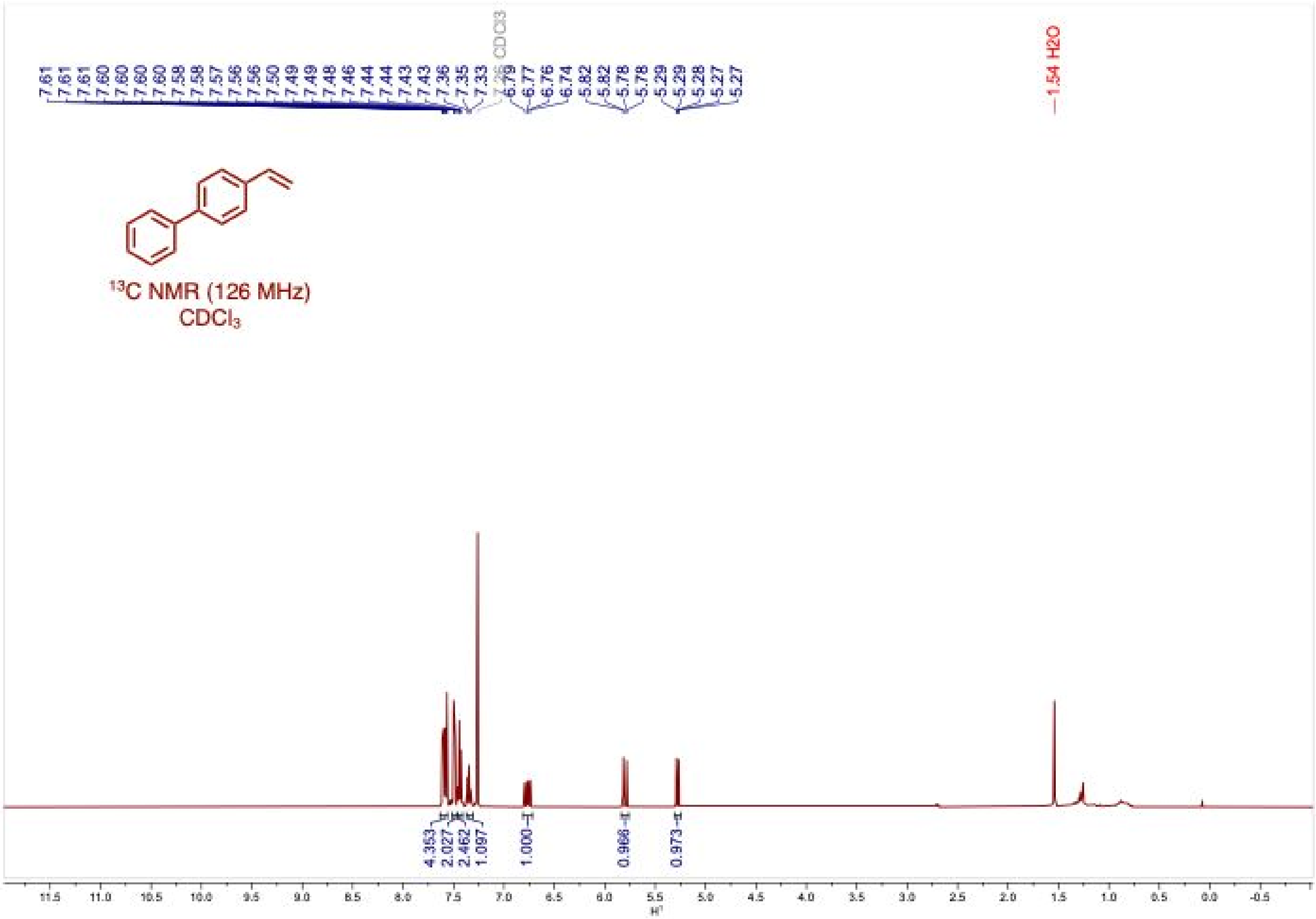

^{13}C (126 MHz, $CDCl_3$) NMR Spectrum of 25

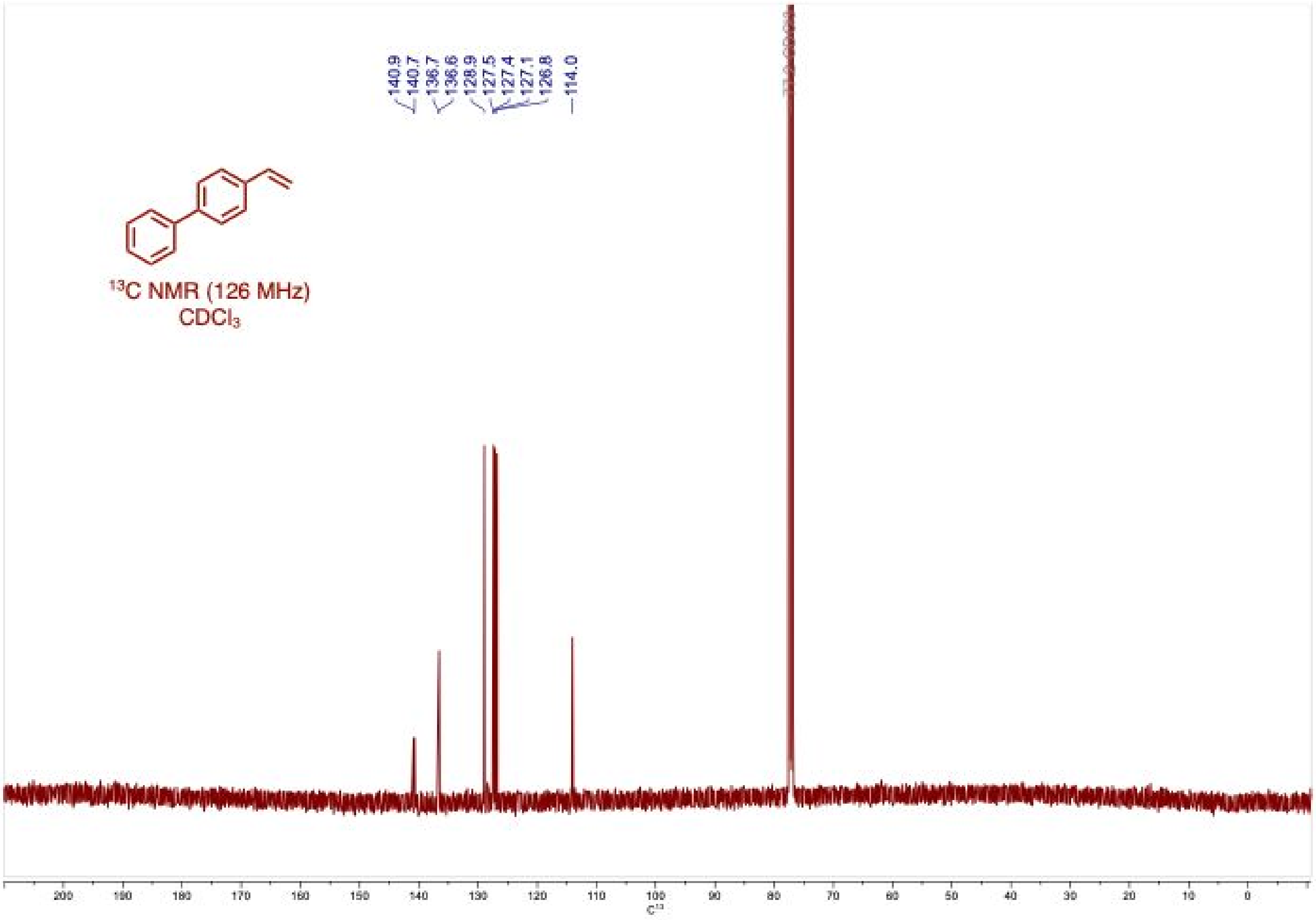

^{1}H (500 MHz, $CDCl_3$) NMR Spectrum of 27

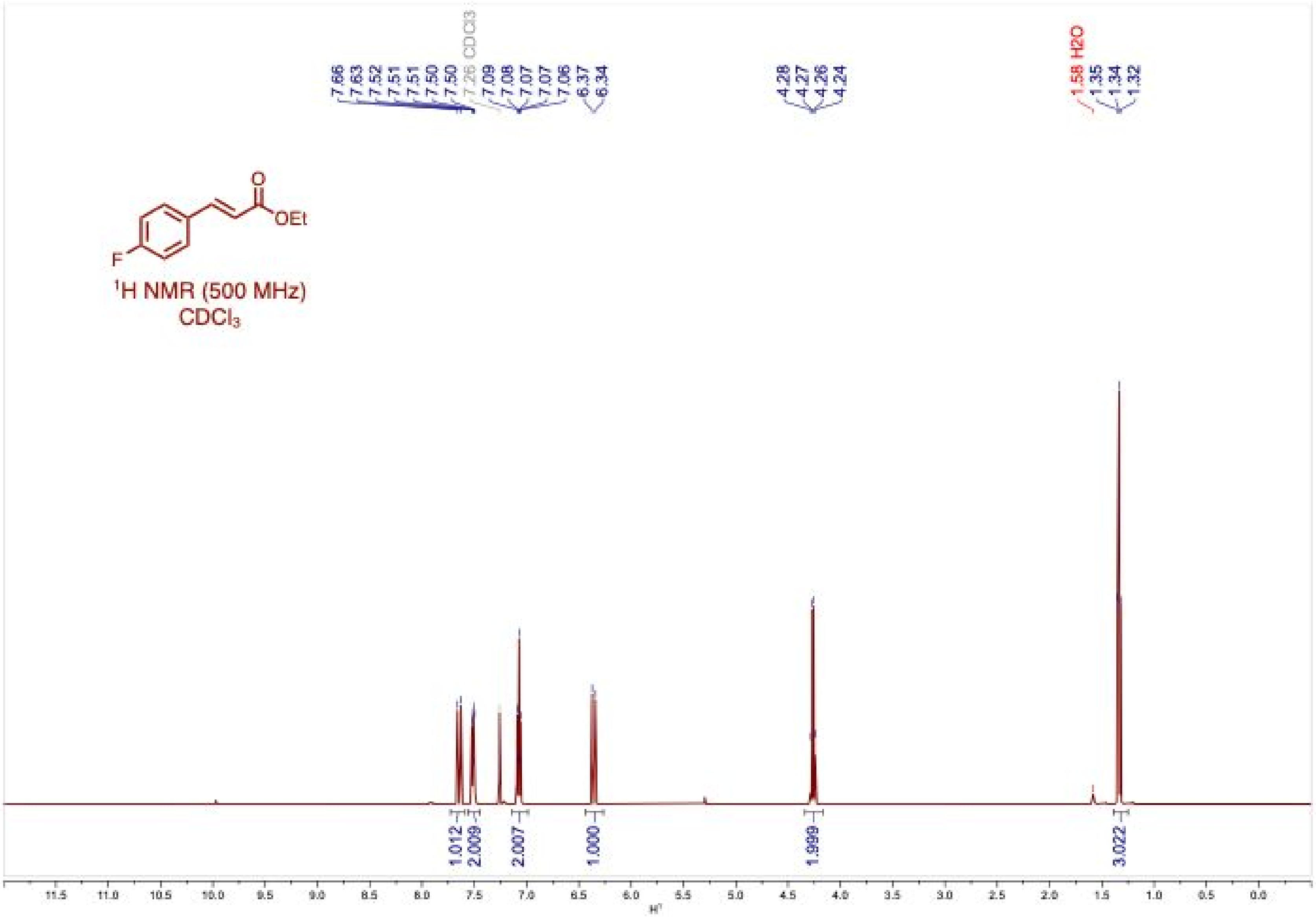

^{13}C (126 MHz, $CDCl_3$) NMR Spectrum of 27

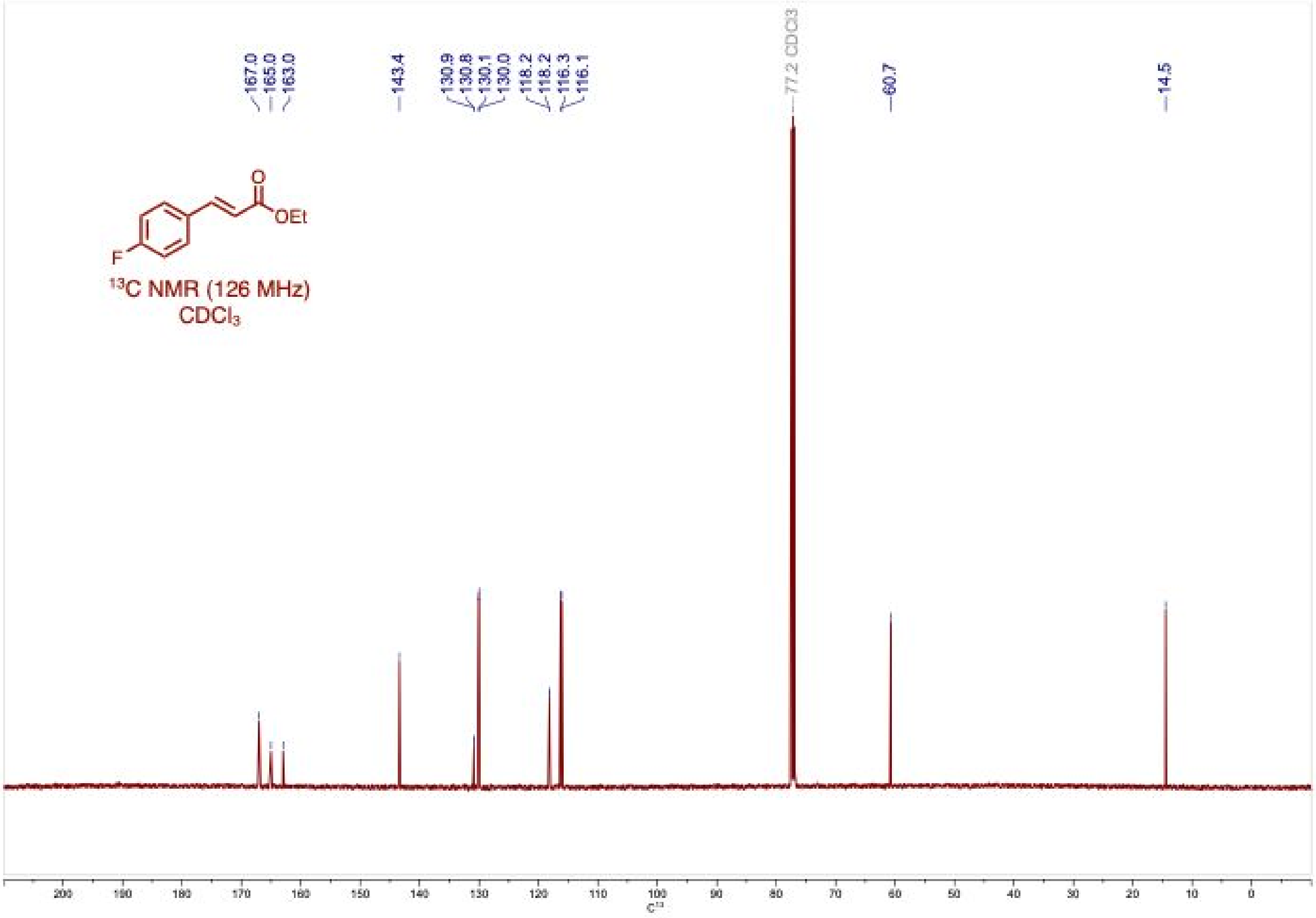

^{19}F (470 MHz, $CDCl_3$) NMR Spectrum of 27

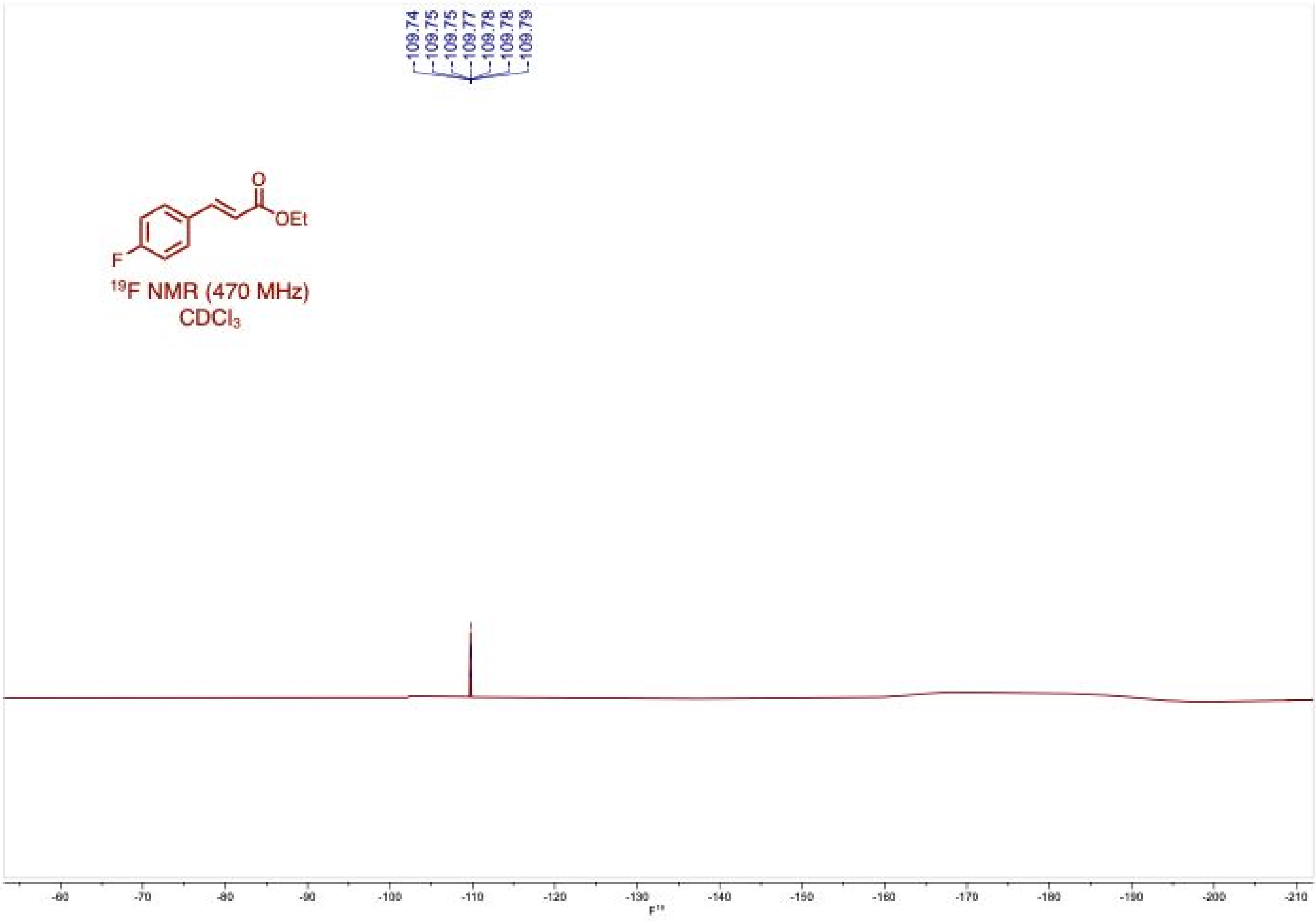

^{1}H (500 MHz, $CDCl_3$) NMR Spectrum of 28

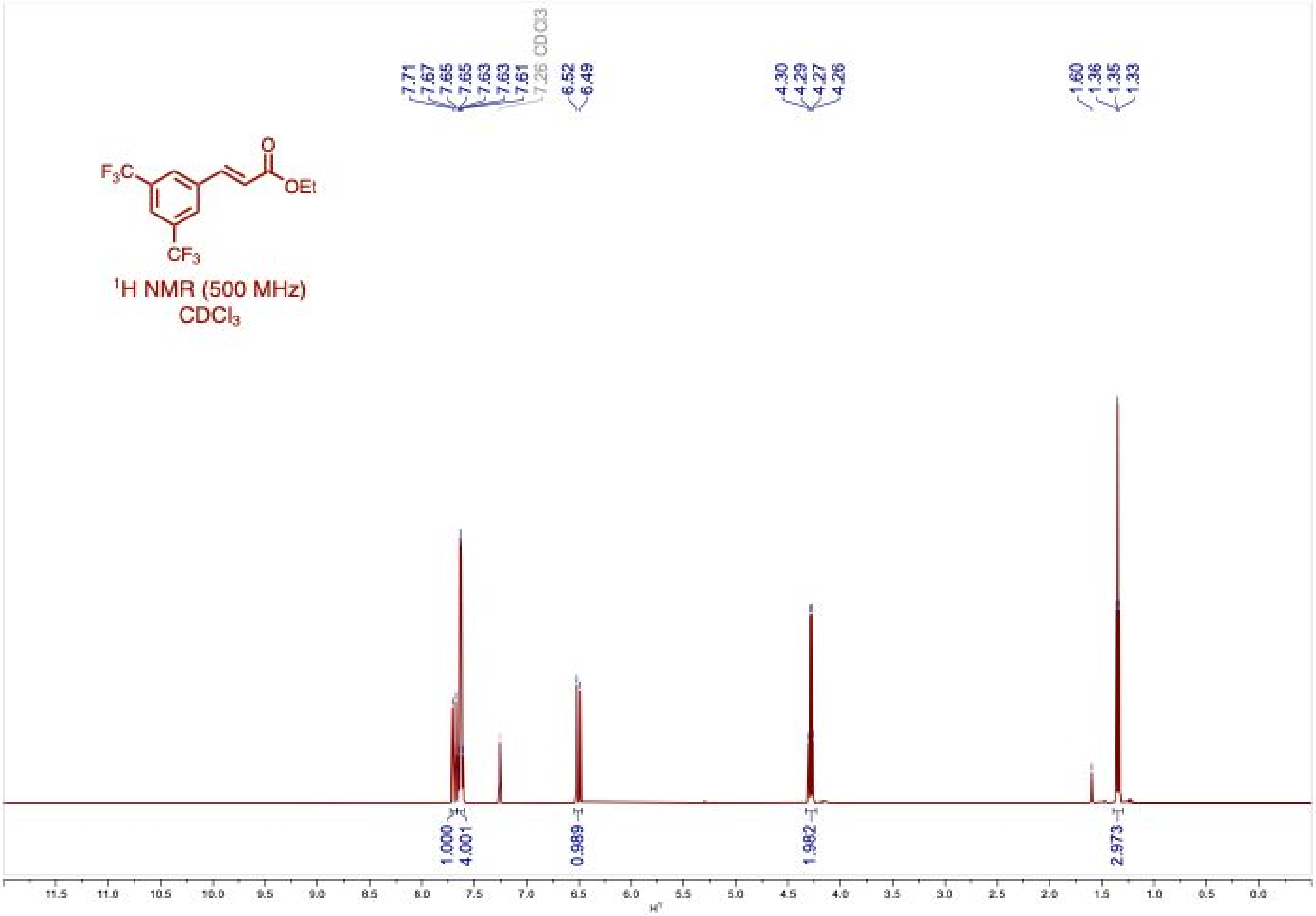

^{13}C (126 MHz, $CDCl_3$) NMR Spectrum of 28

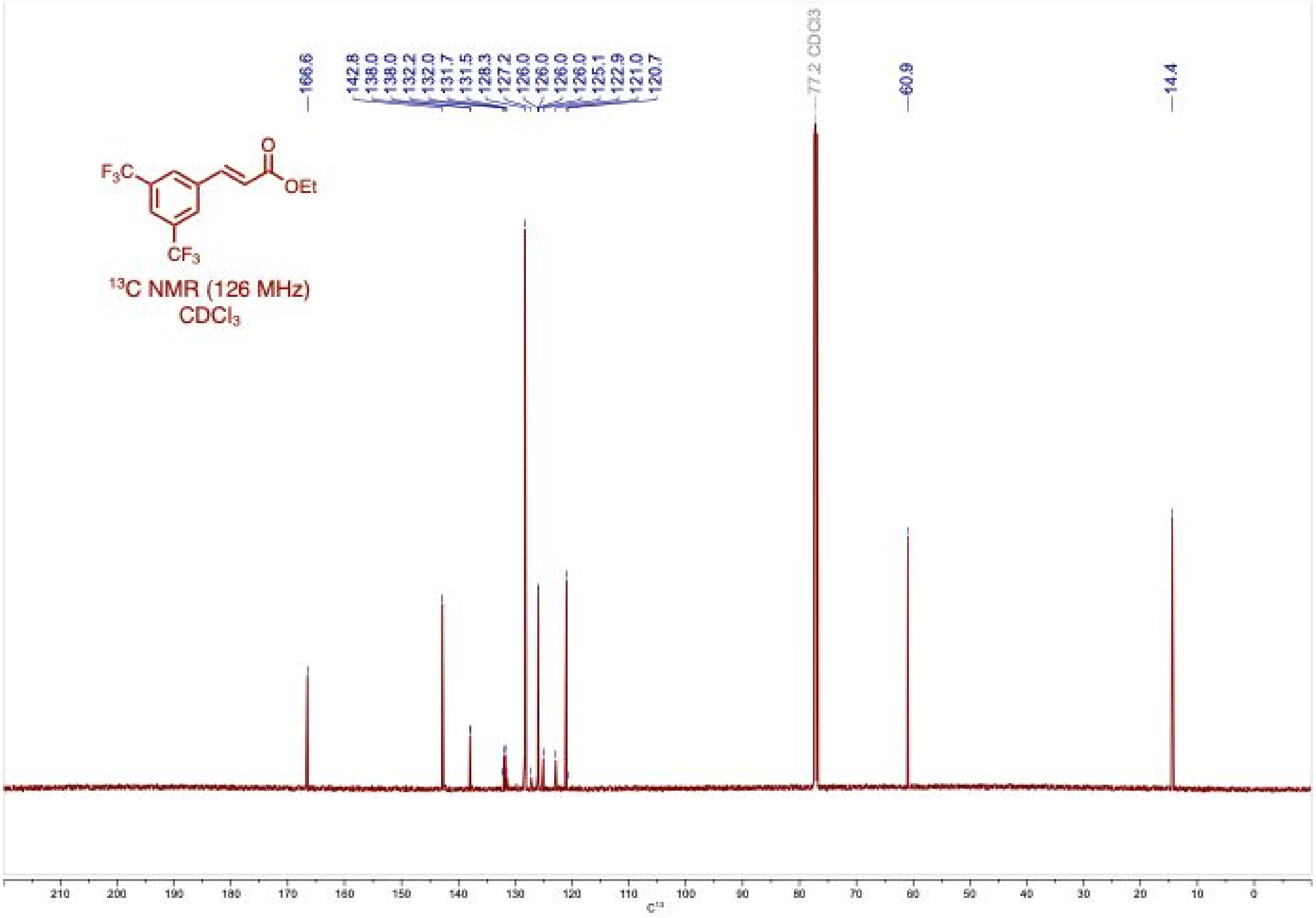

^{19}F (470 MHz, $CDCl_3$) NMR Spectrum of 28

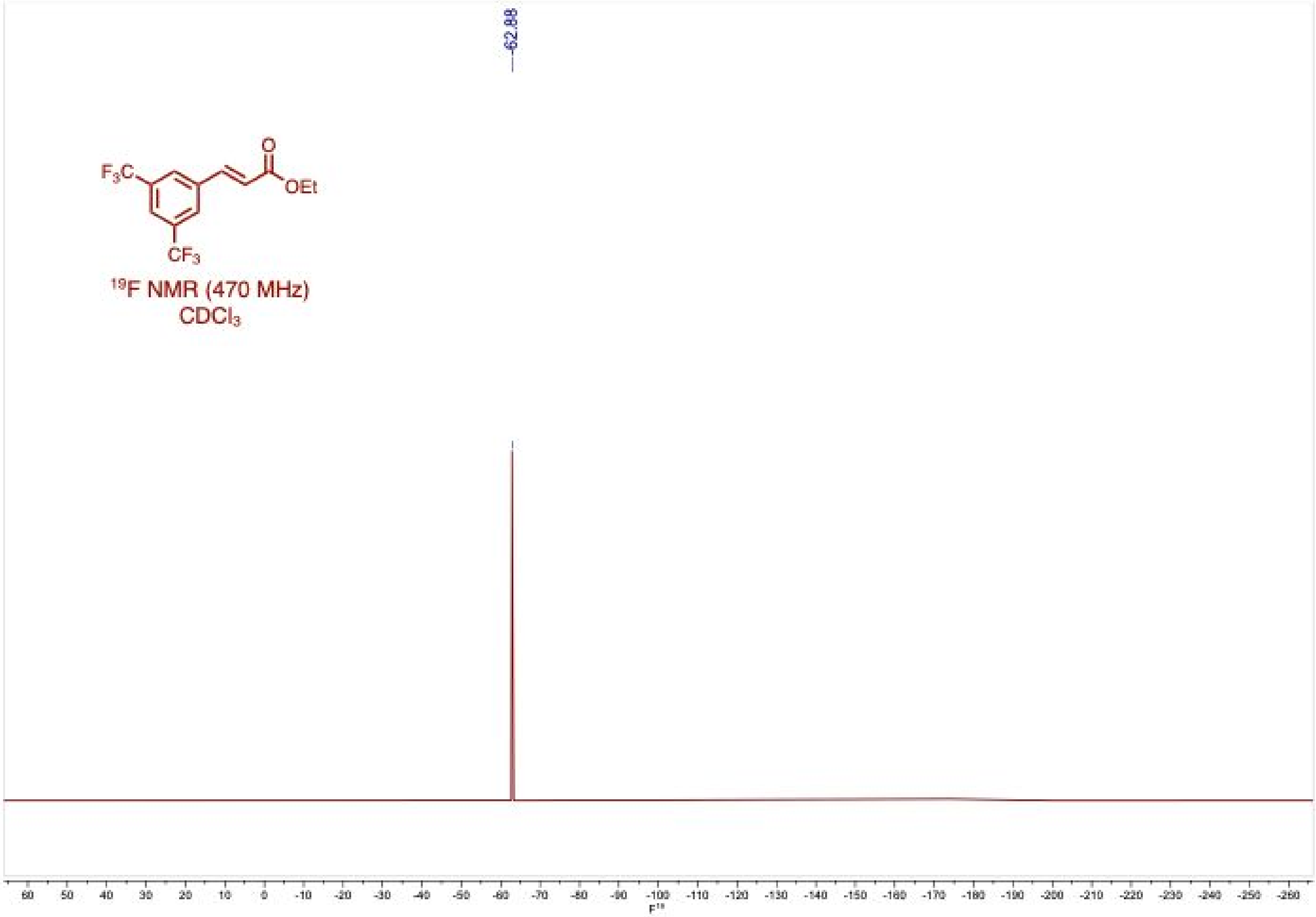

^{1}H (500 MHz, $CDCl_3$) NMR Spectrum of 29

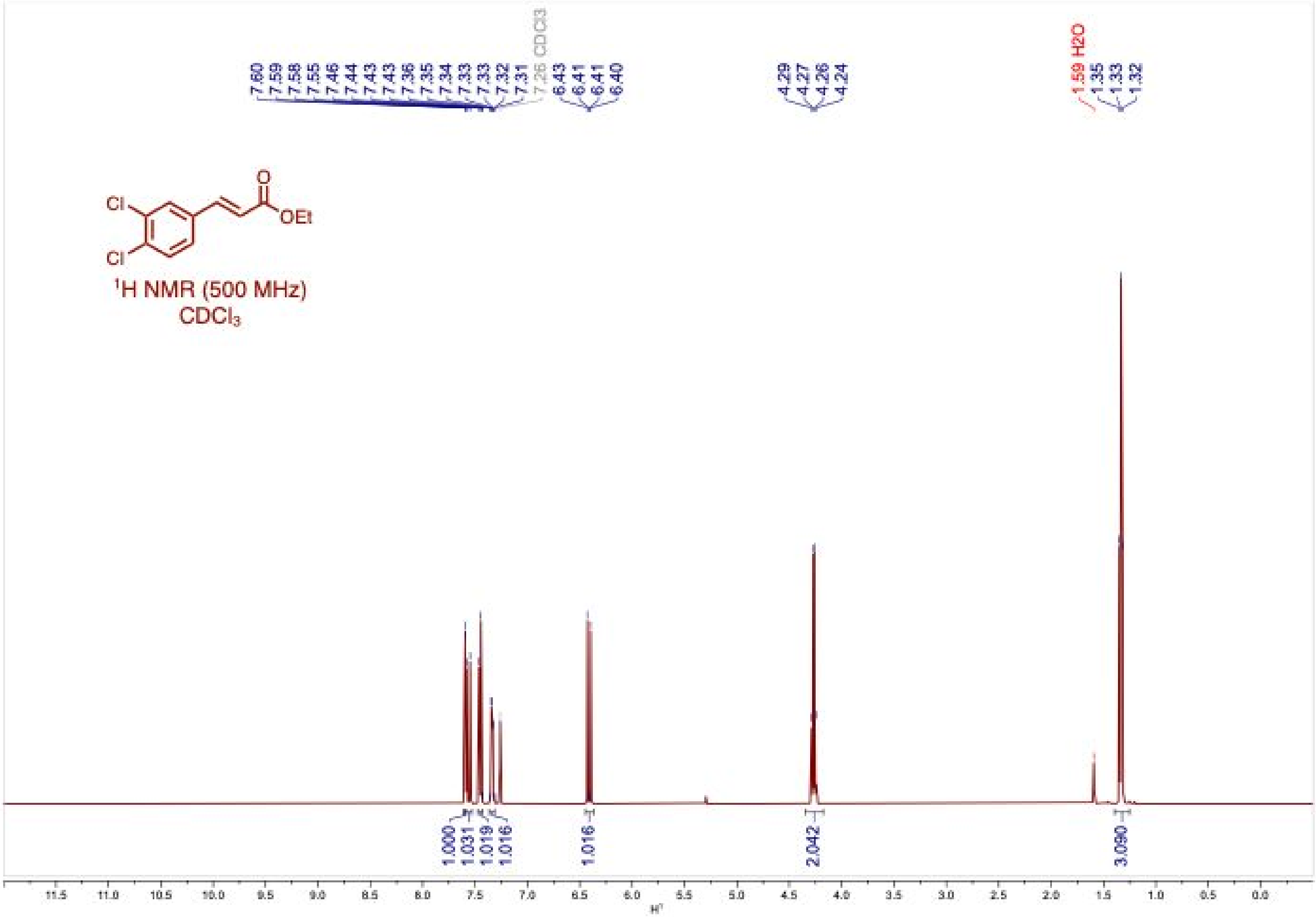

^{13}C (126 MHz, $CDCl_3$) NMR Spectrum of 29

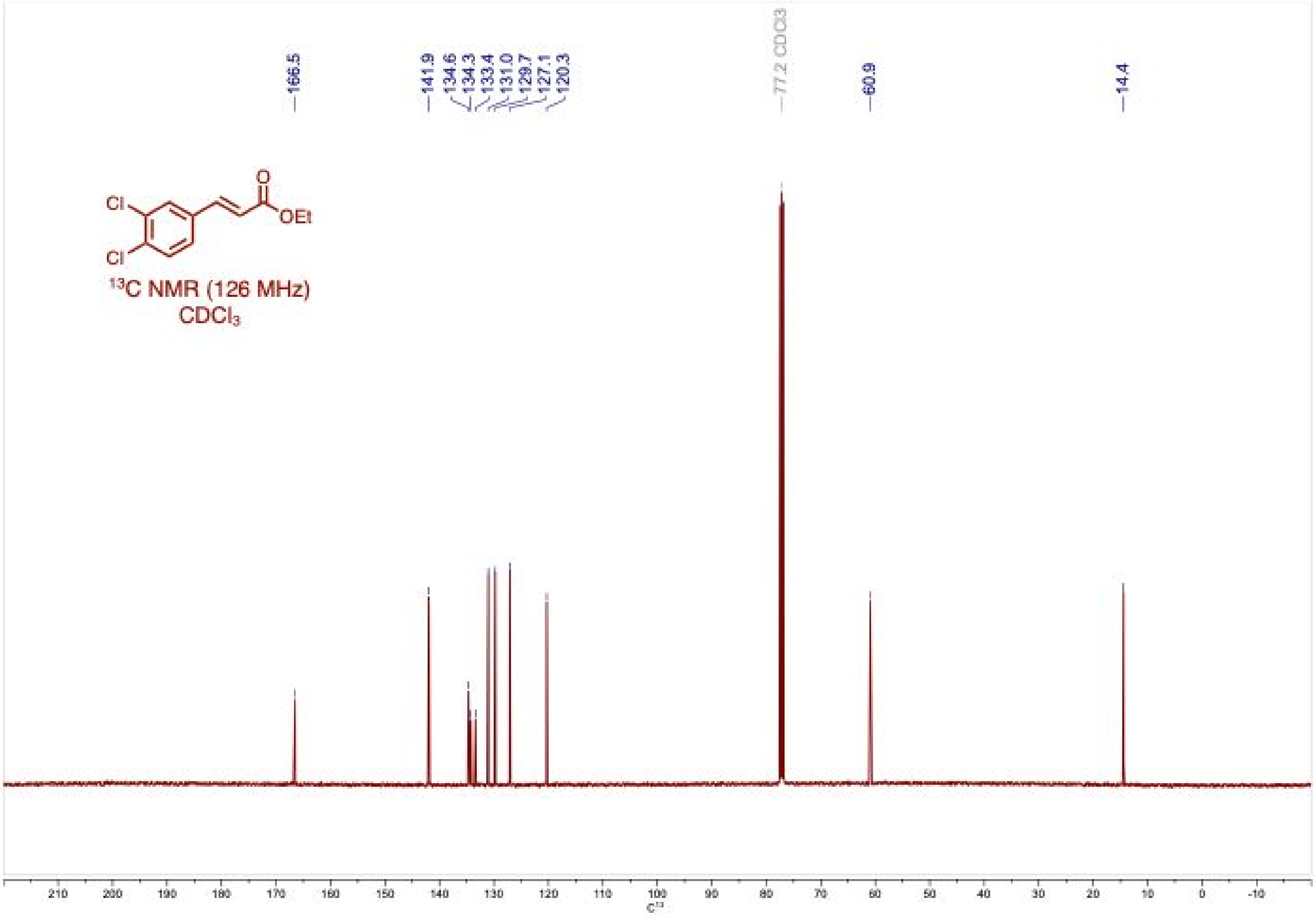

^{1}H (500 MHz, $CDCl_3$) NMR Spectrum of 30

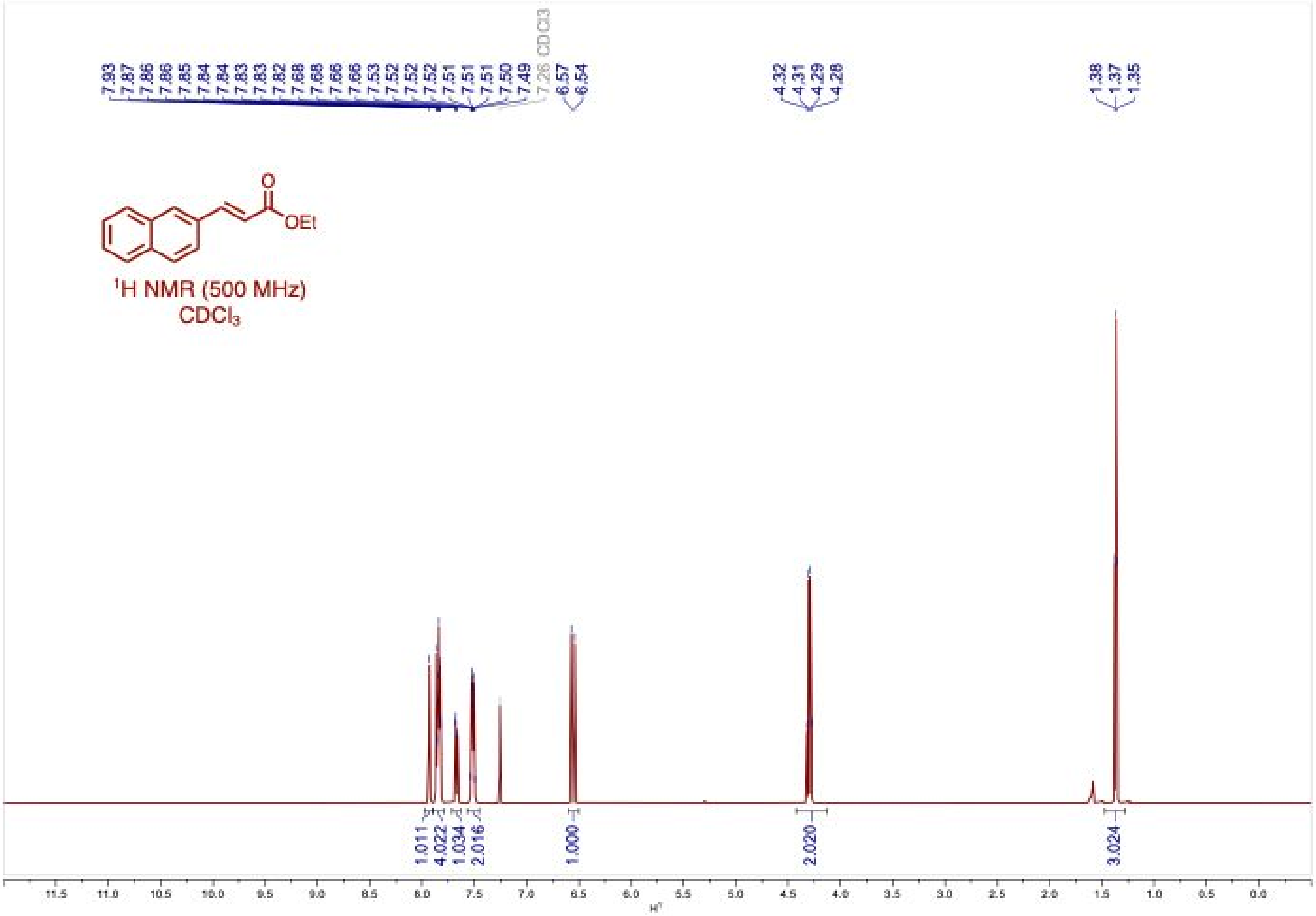

^{13}C (126 MHz, $CDCl_3$) NMR Spectrum of 30

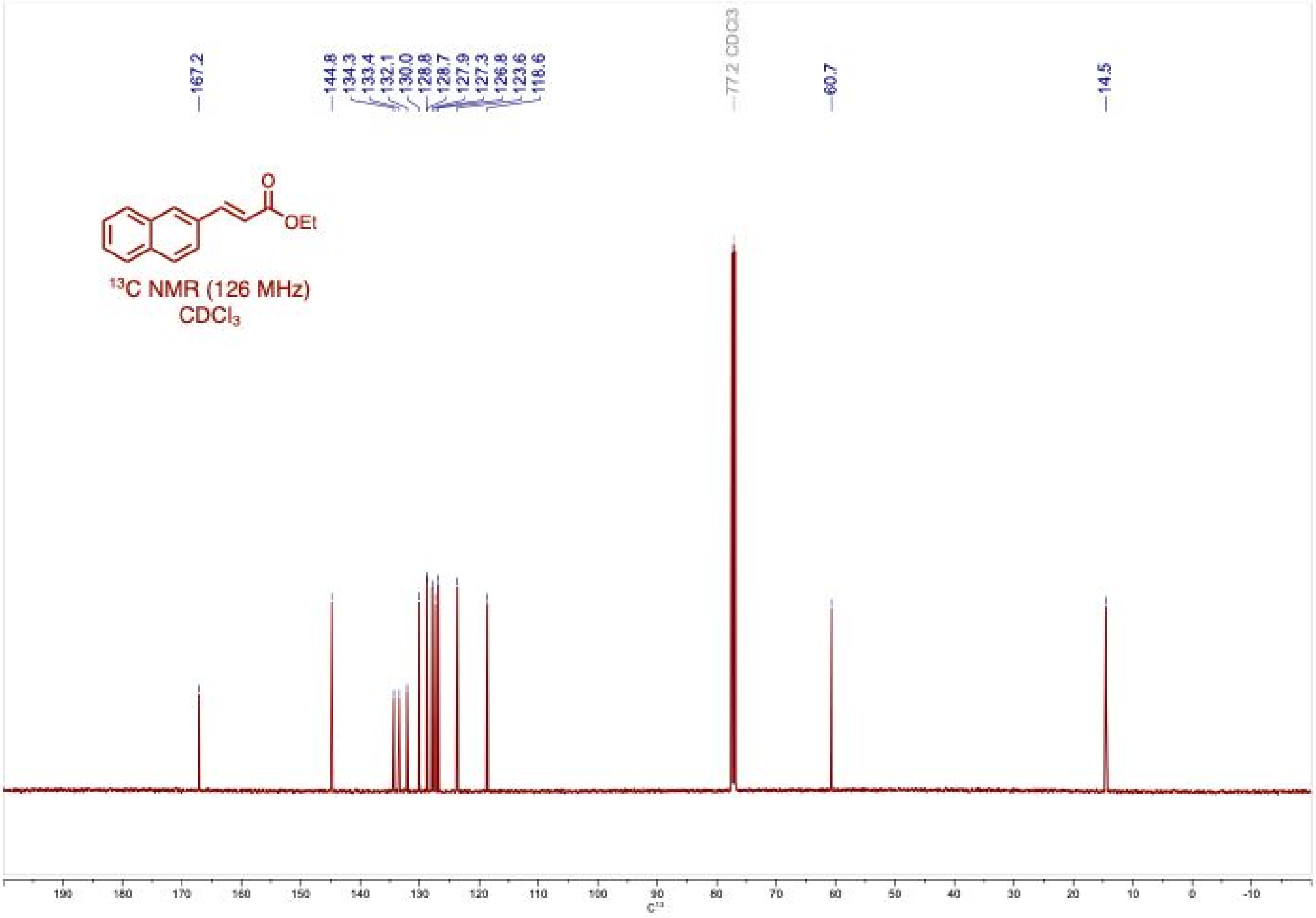

^{1}H (500 MHz, $CDCl_3$) NMR Spectrum of 31

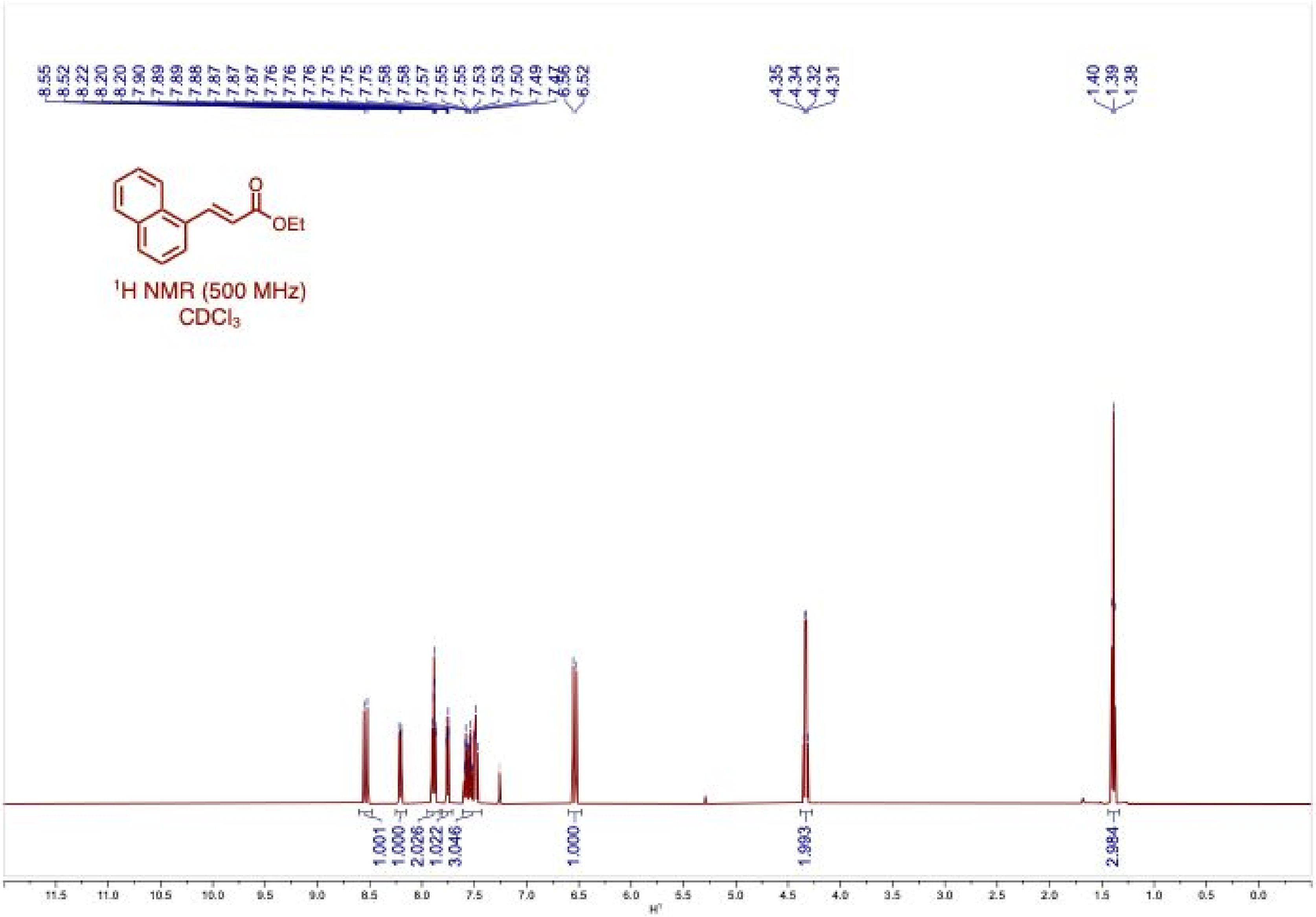

^{13}C (126 MHz, $CDCl_3$) NMR Spectrum of 31

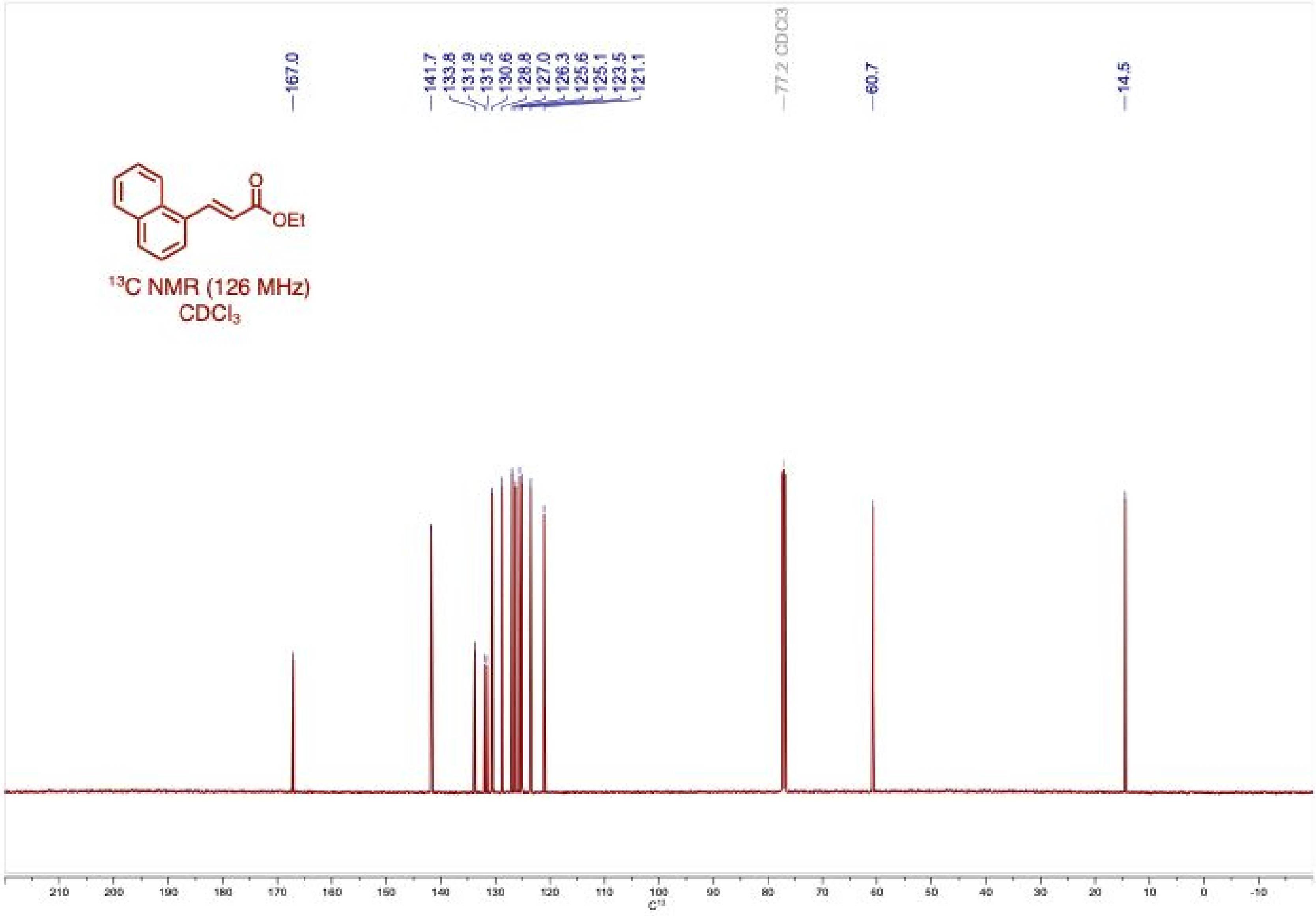

^{1}H (500 MHz, $CDCl_3$) NMR Spectrum of 32

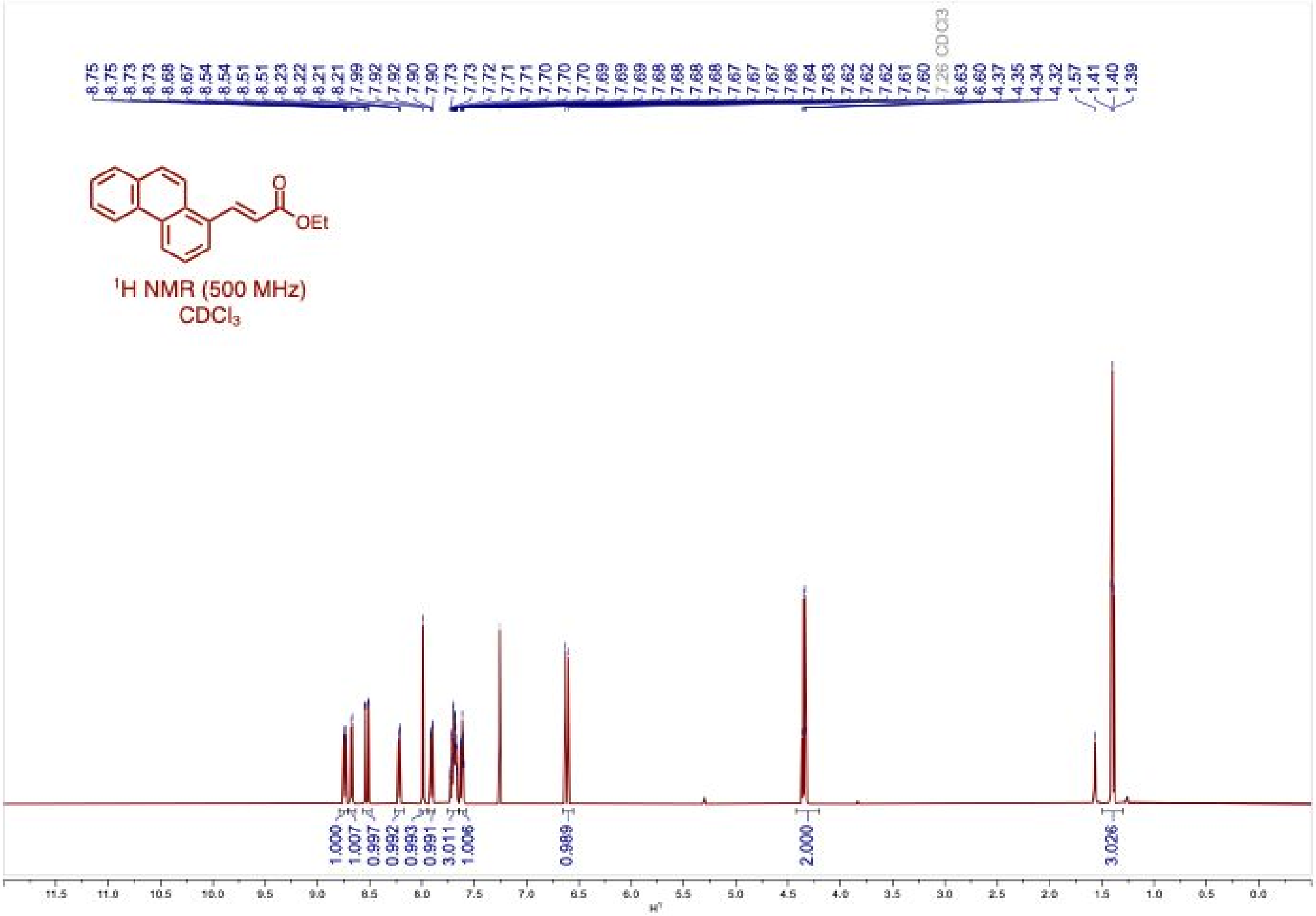

^{13}C (126 MHz, $CDCl_3$) NMR Spectrum of 32

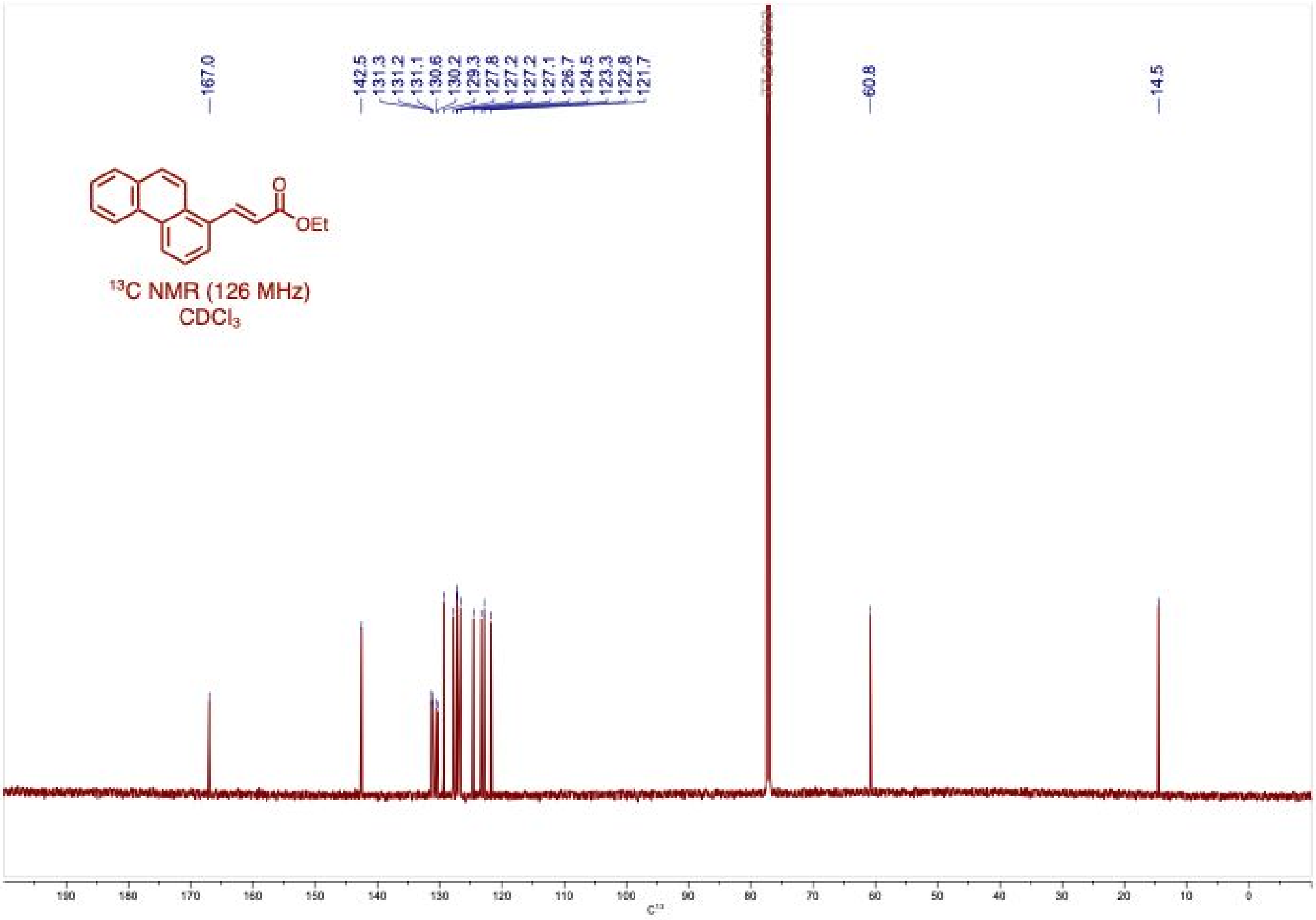

^{1}H (500 MHz, $CDCl_3$) NMR Spectrum of 33

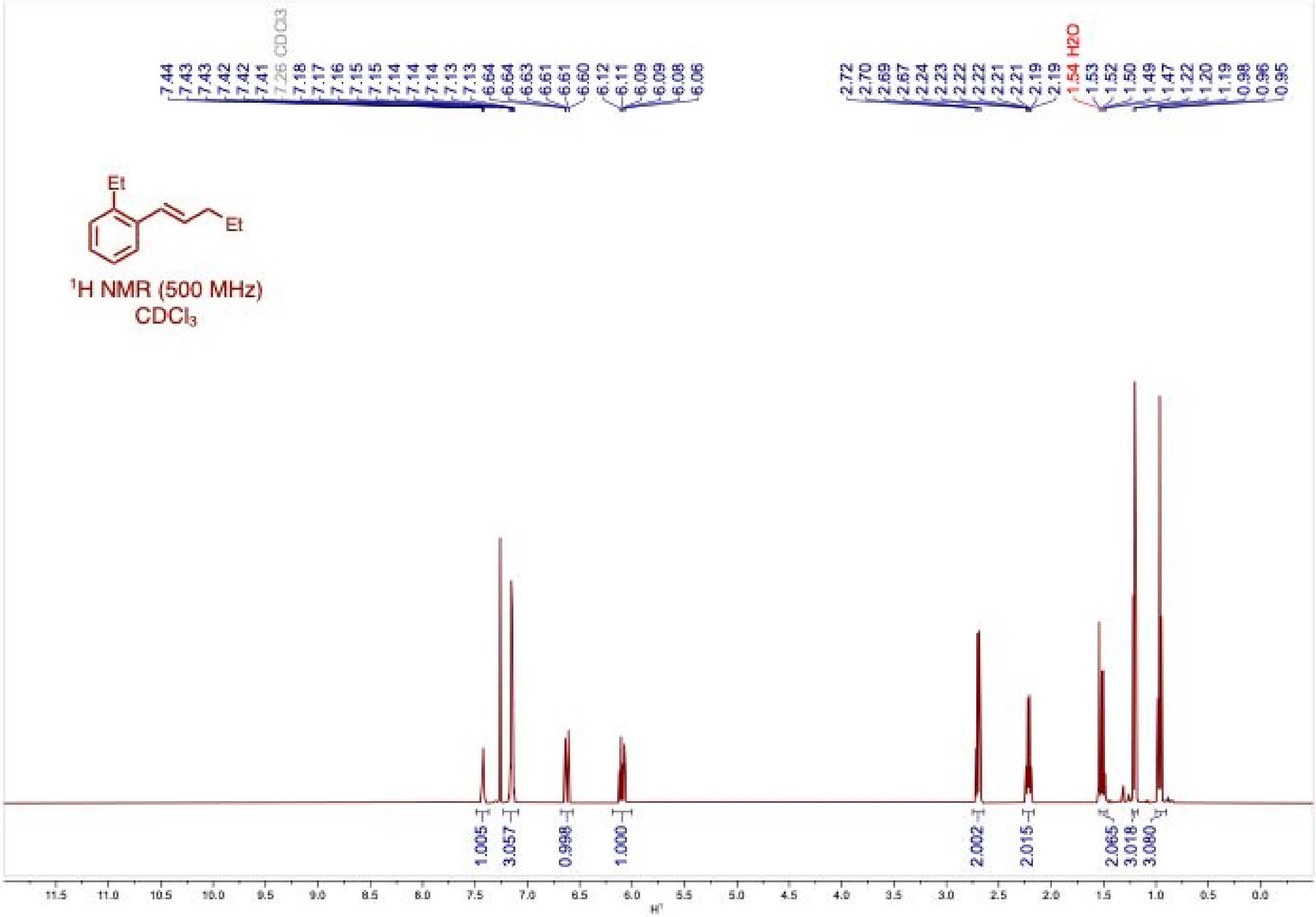

^{13}C (126 MHz, $CDCl_3$) NMR Spectrum of 33

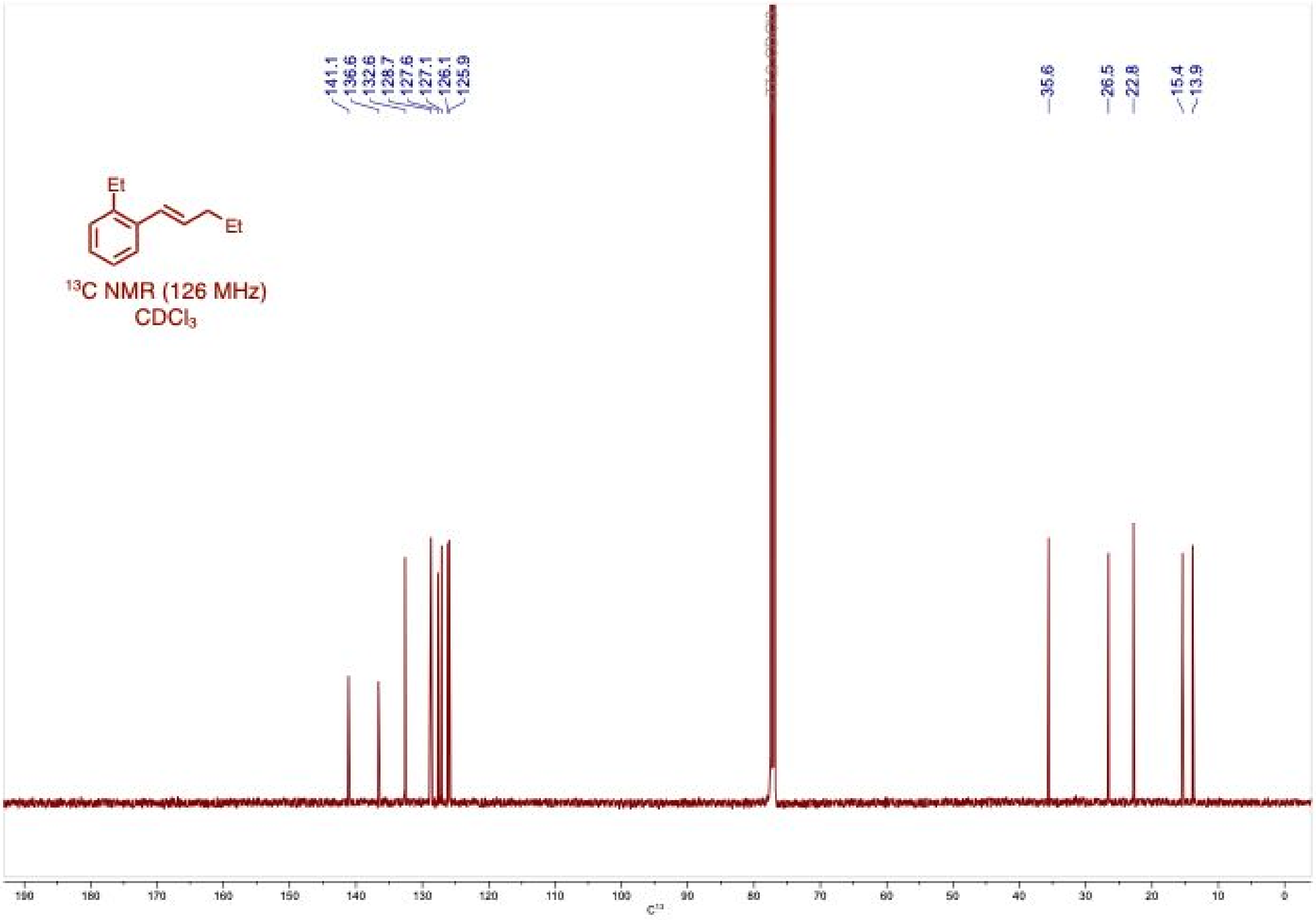

^{1}H (500 MHz, $CDCl_3$) NMR Spectrum of 34

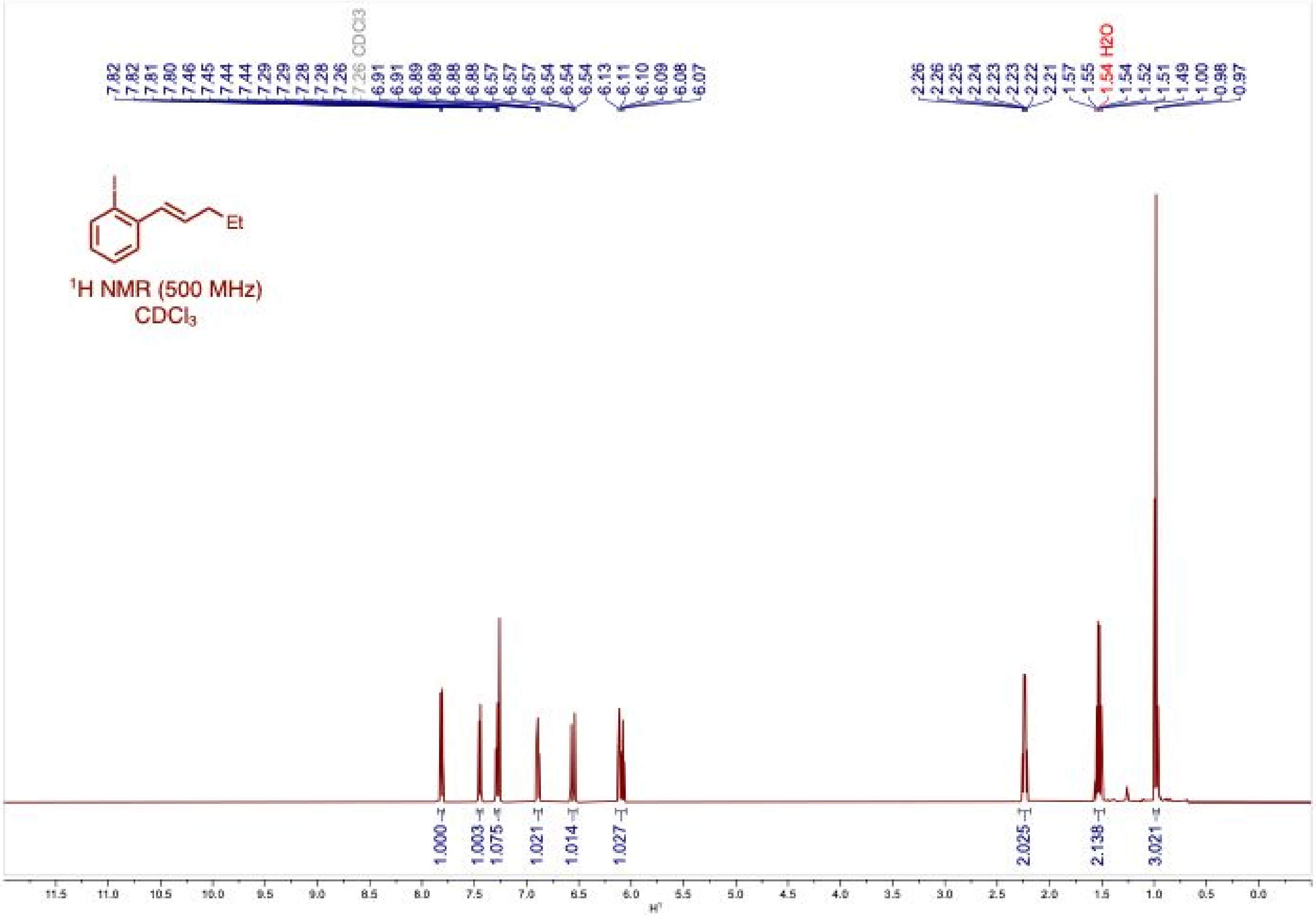

^{13}C (126 MHz, $CDCl_3$) NMR Spectrum of 34

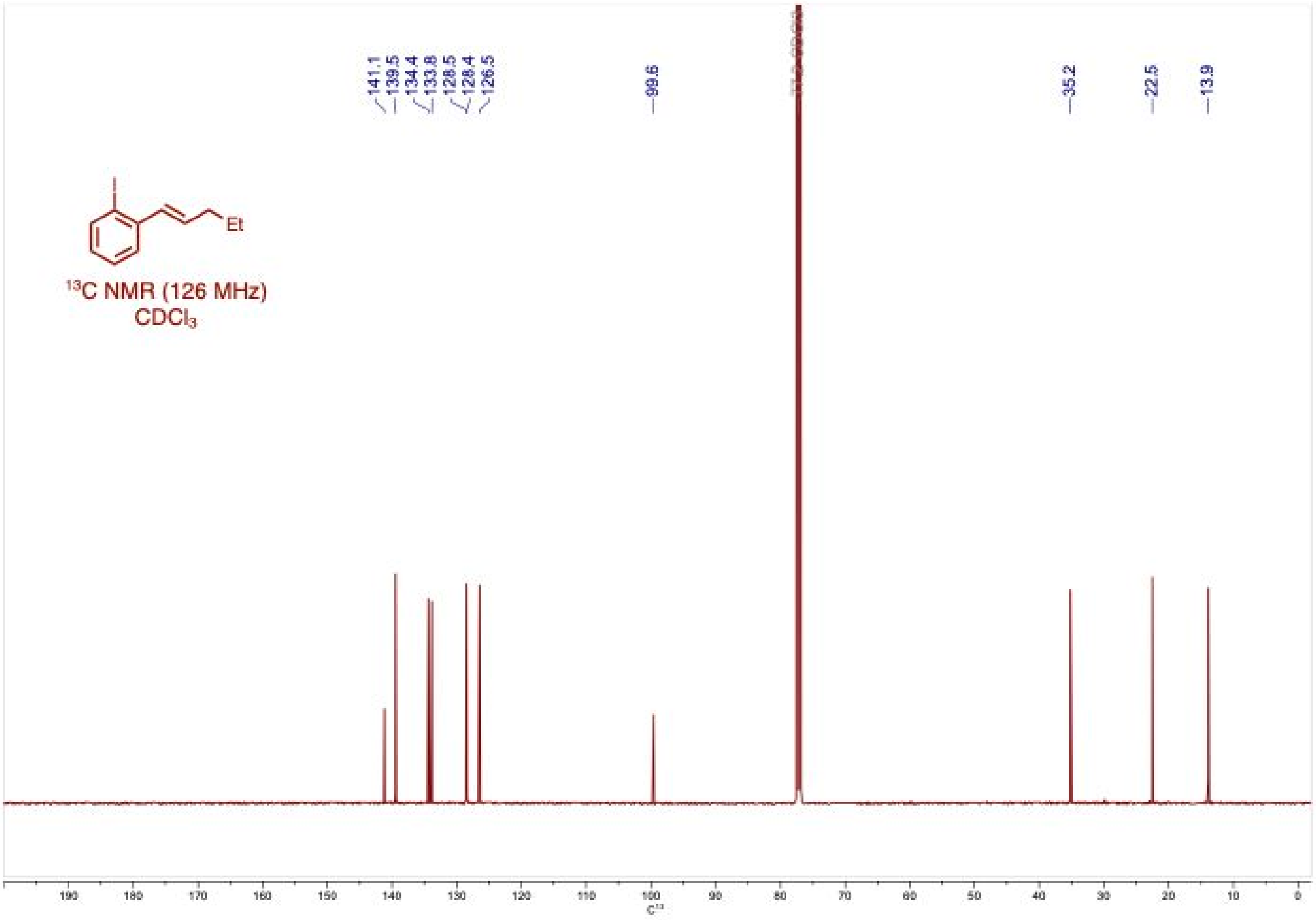

^1H (500 MHz, $CDCl_3$) NMR Spectrum of 35

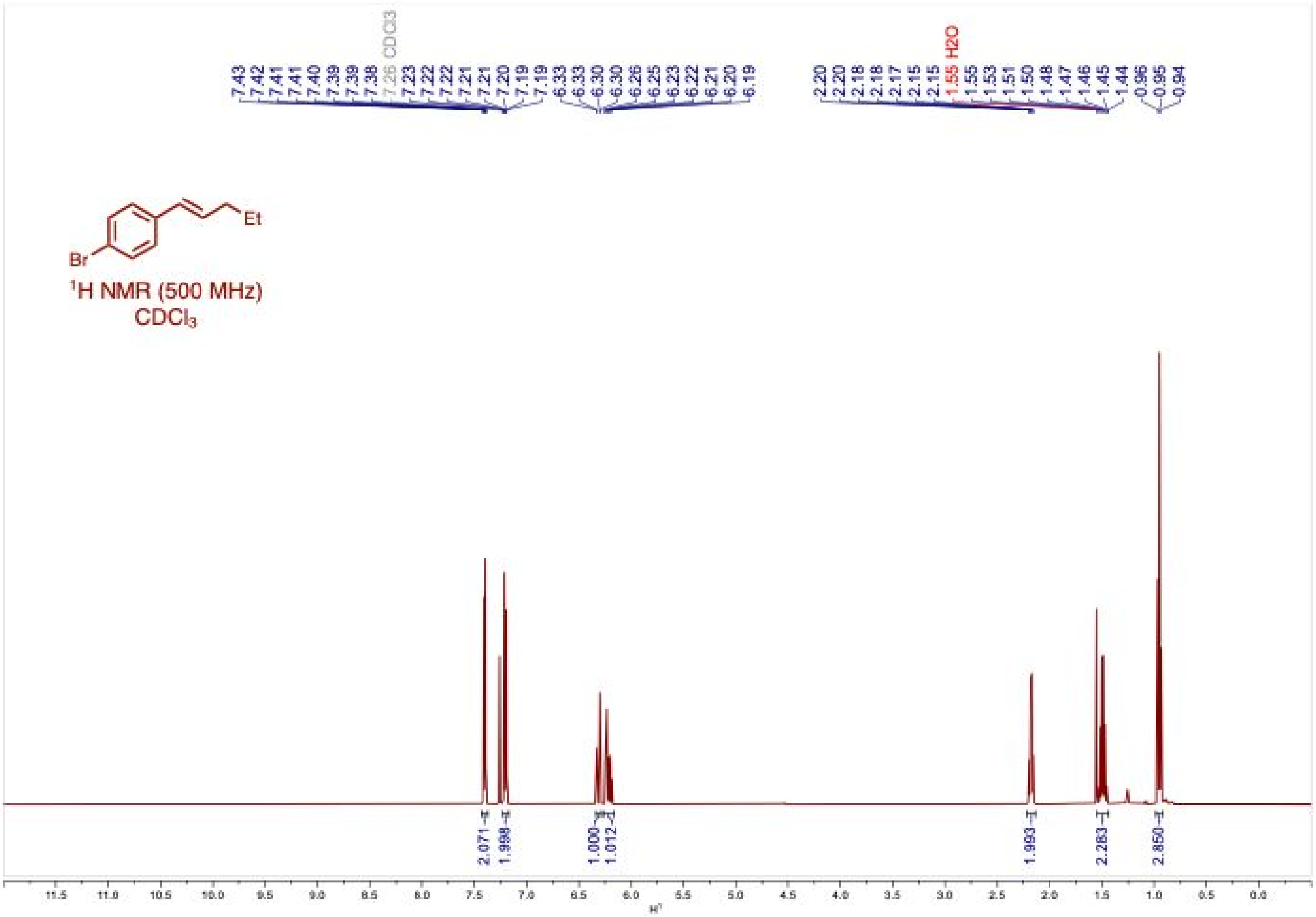

^{13}C (126 MHz, $CDCl_3$) NMR Spectrum of 35

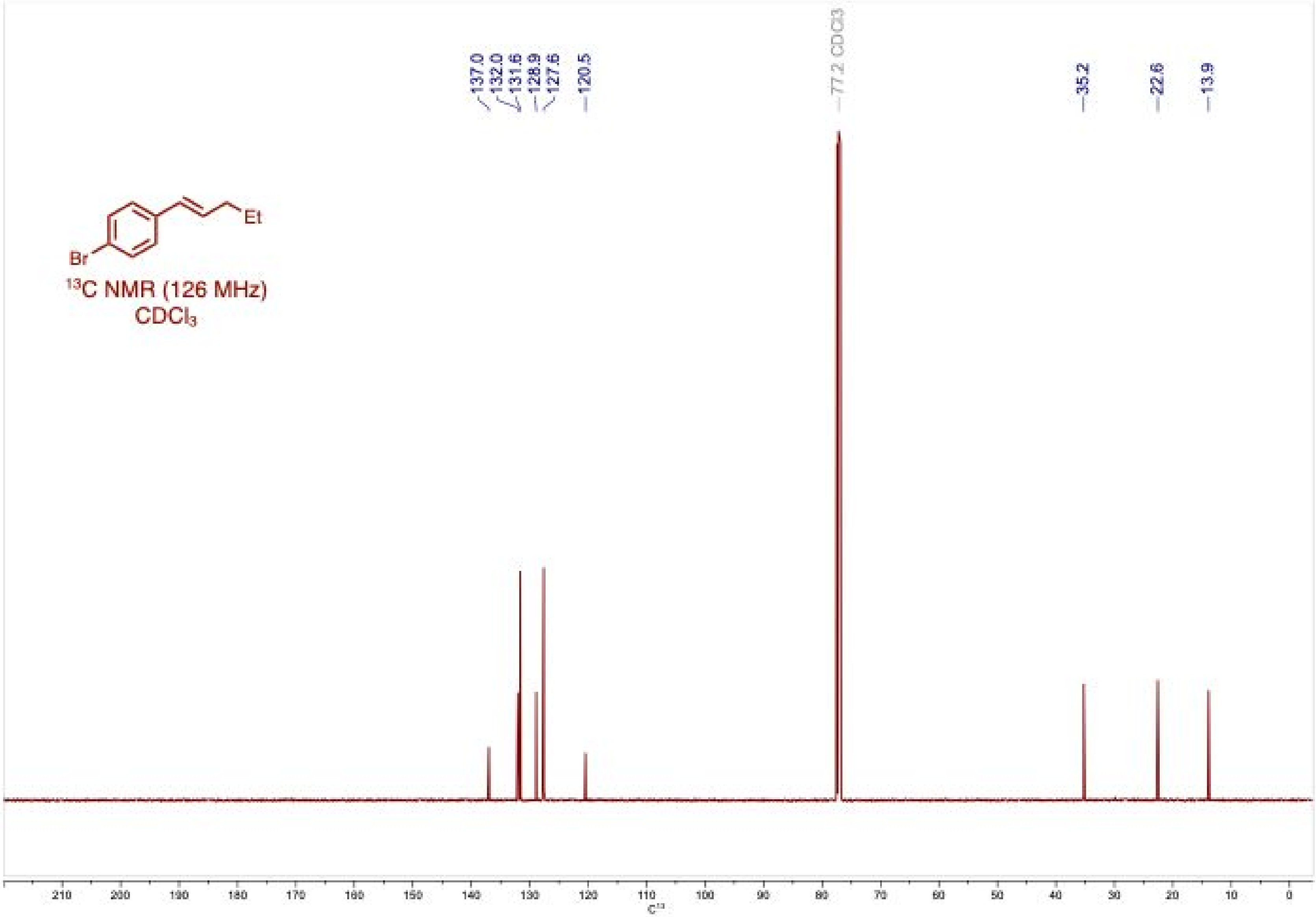

^{1}H (500 MHz, CDCl$_3$) NMR Spectrum of 36

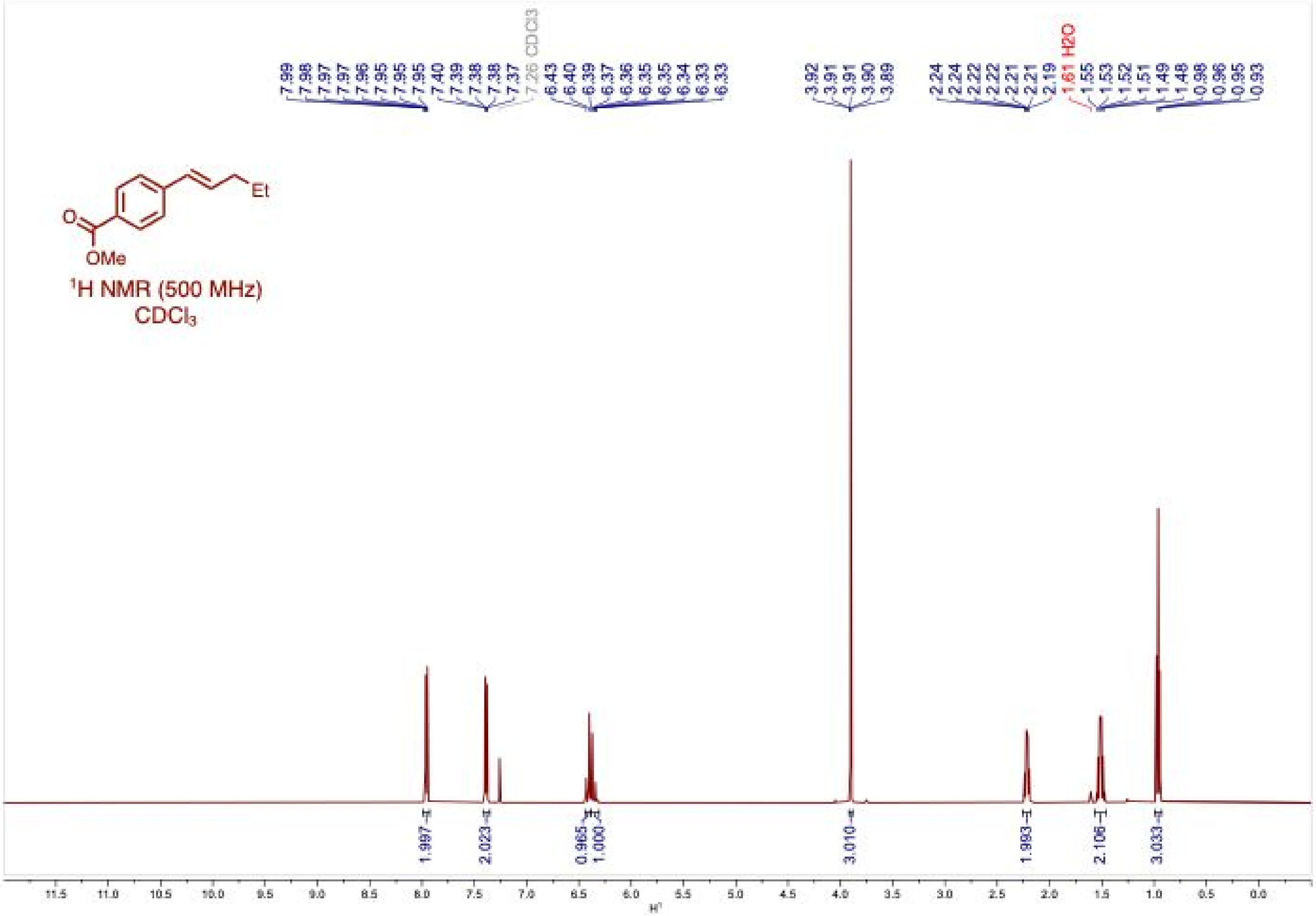

^{13}C (126 MHz, $CDCl_3$) NMR Spectrum of 36

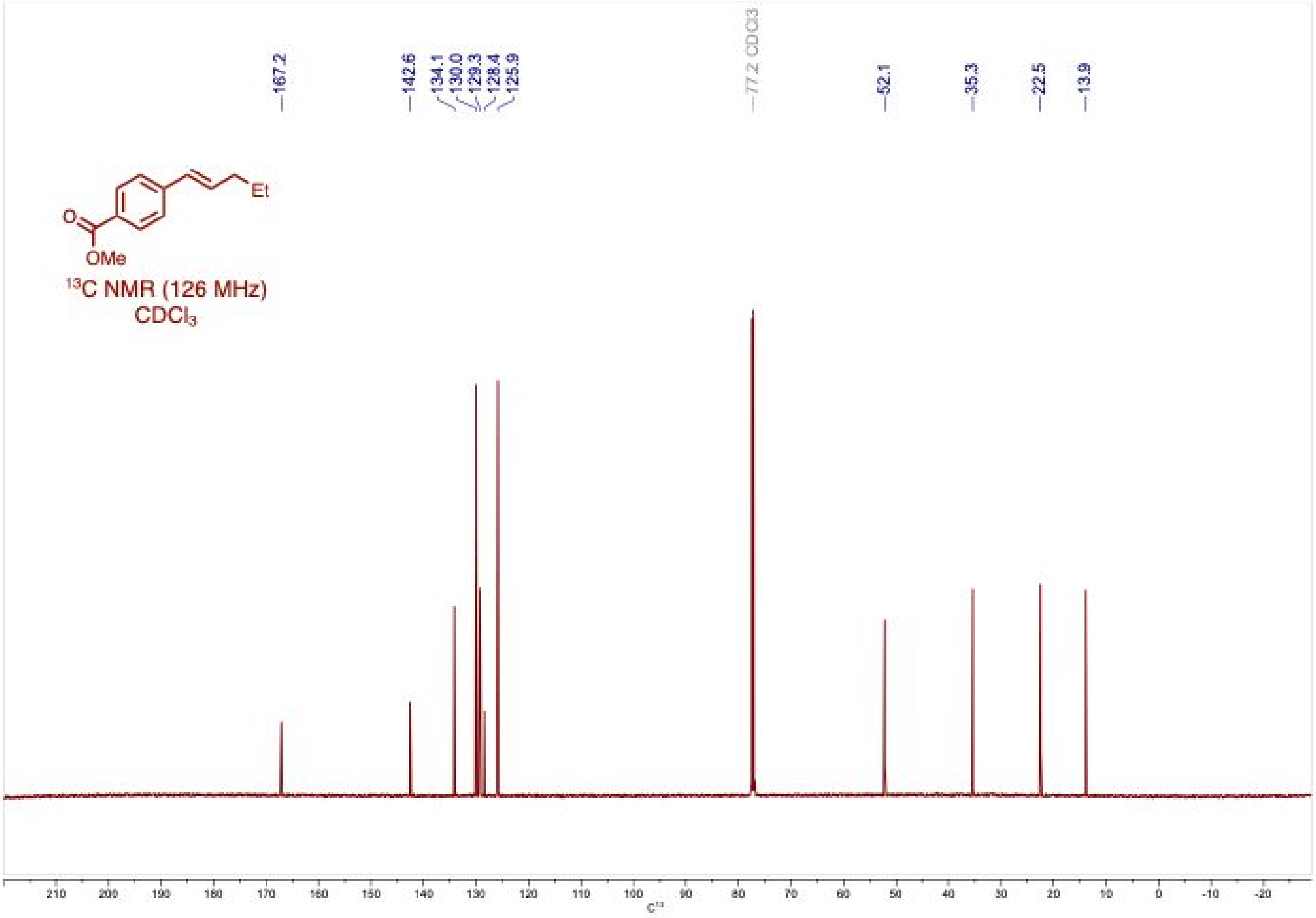

^{1}H (500 MHz, $CDCl_3$) NMR Spectrum of 37

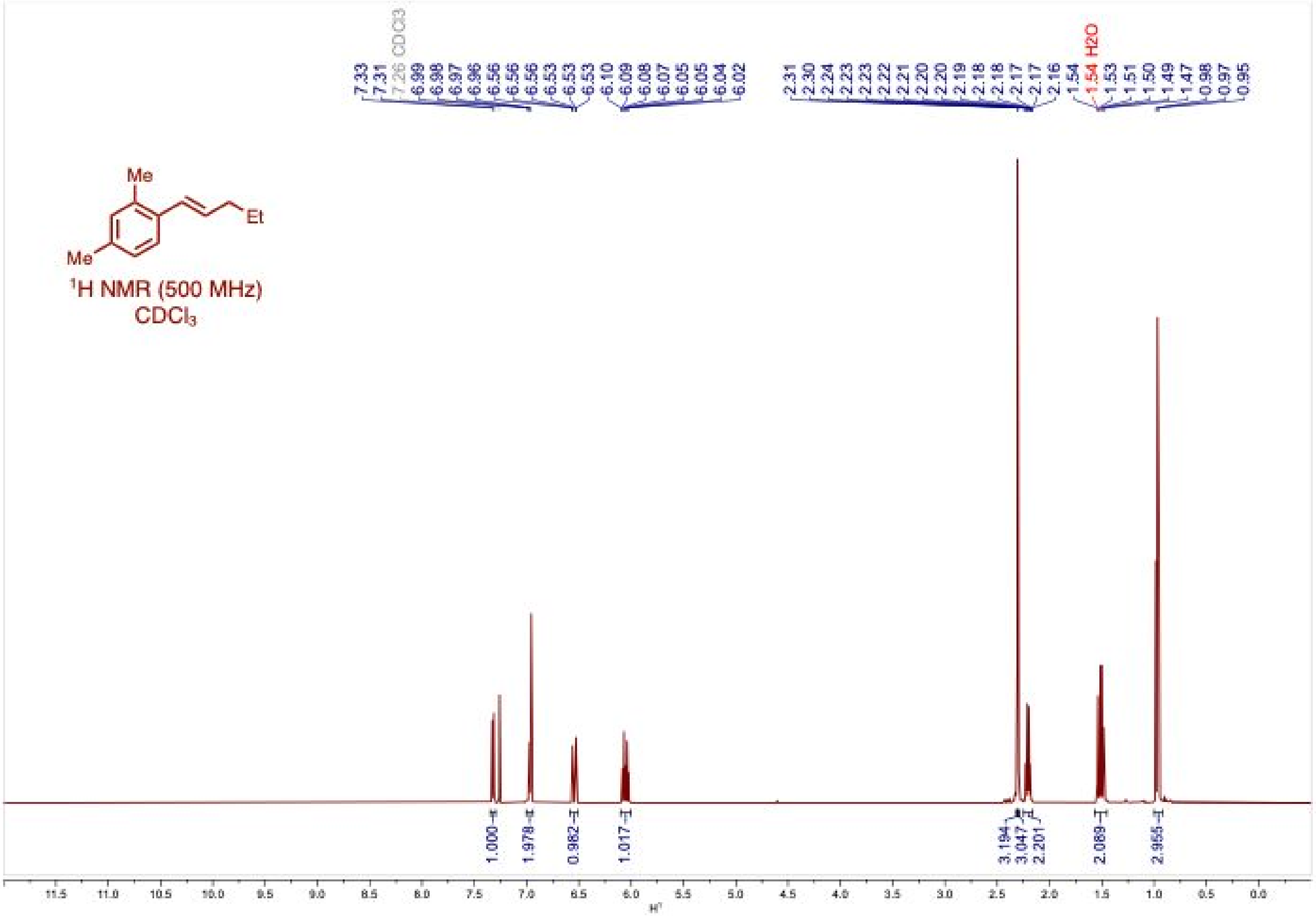

^{13}C (126 MHz, $CDCl_3$) NMR Spectrum of 37

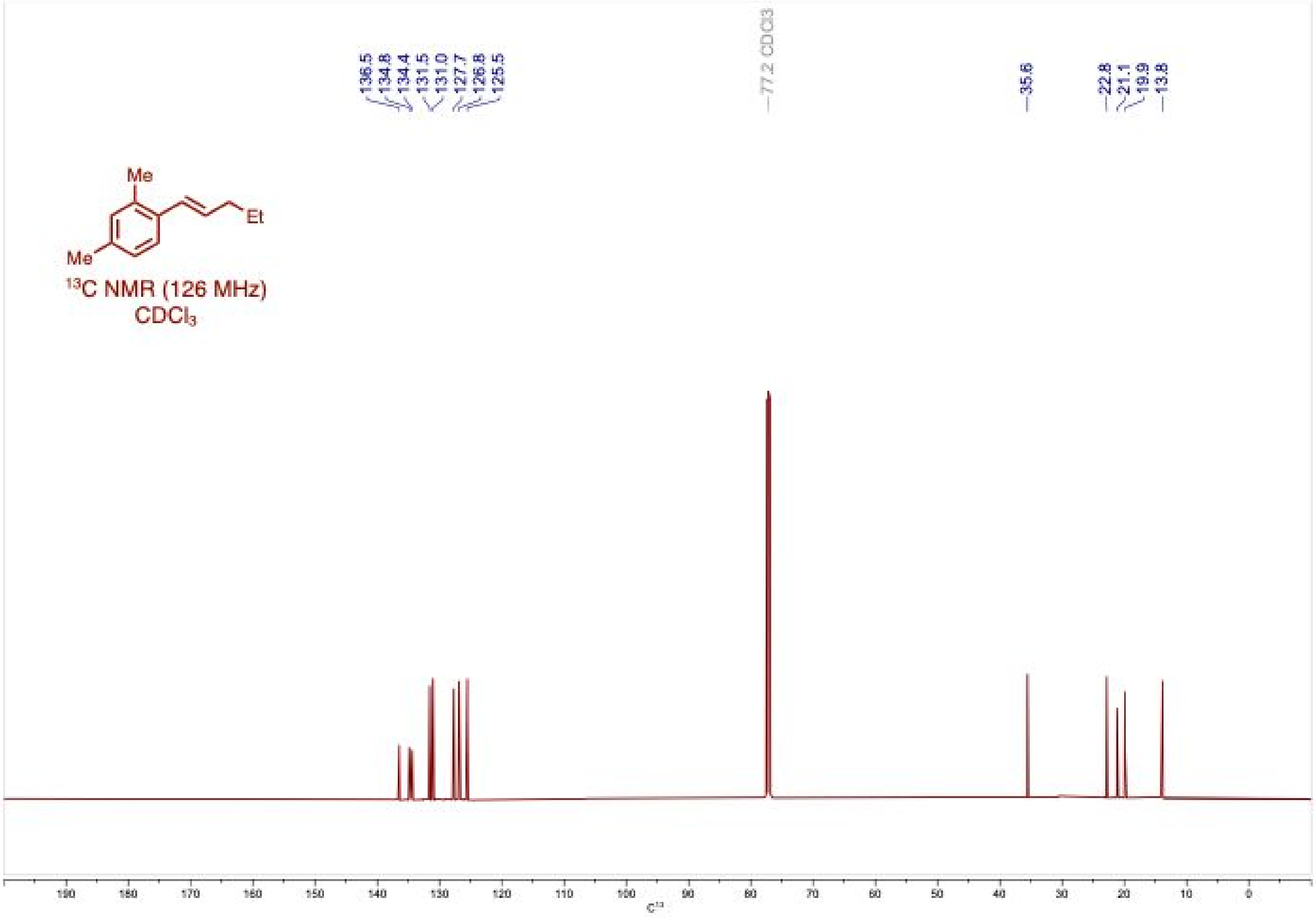

^{1}H (500 MHz, CDCl$_3$) NMR Spectrum of 38

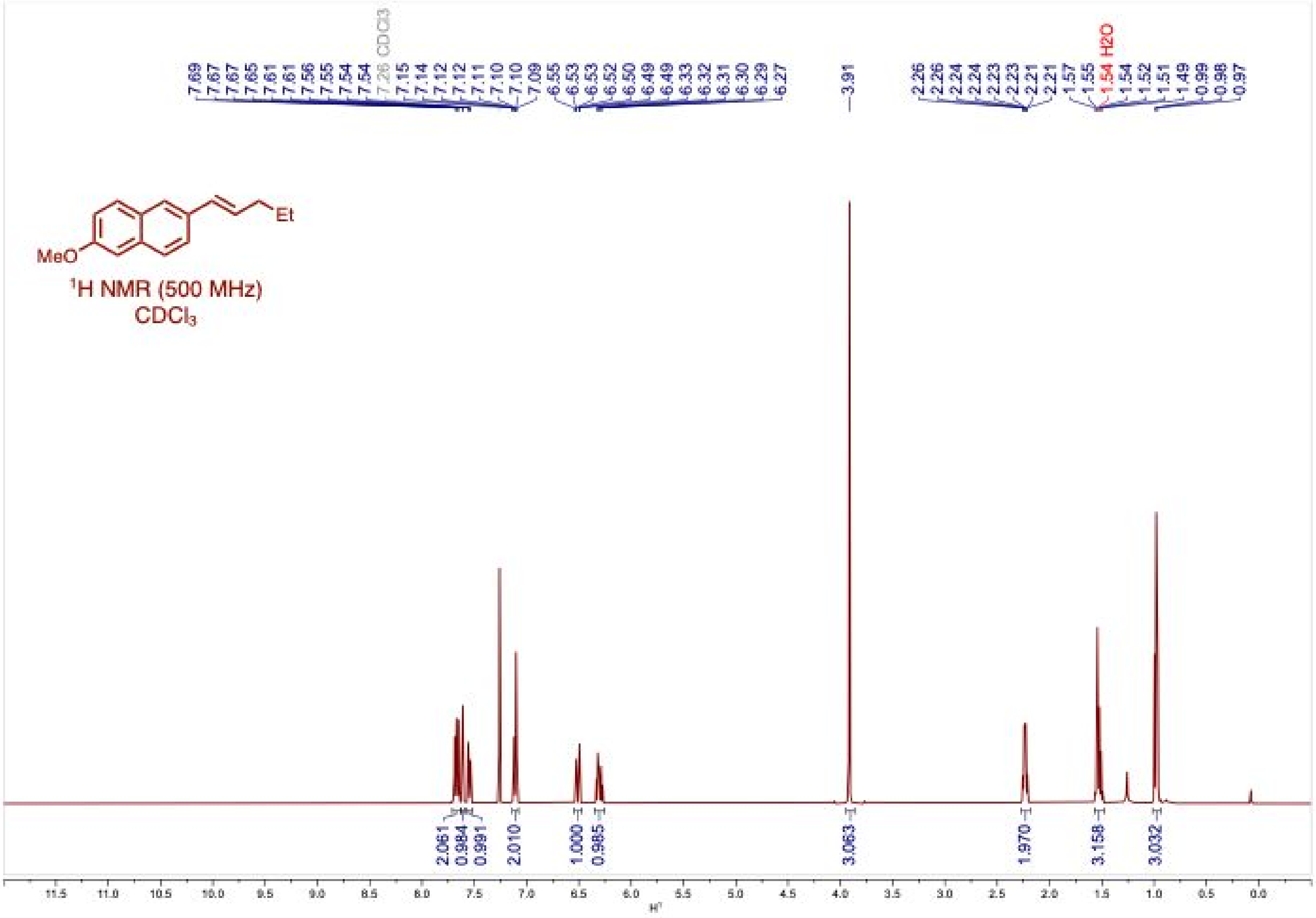

^{13}C (126 MHz, $CDCl_3$) NMR Spectrum of 38

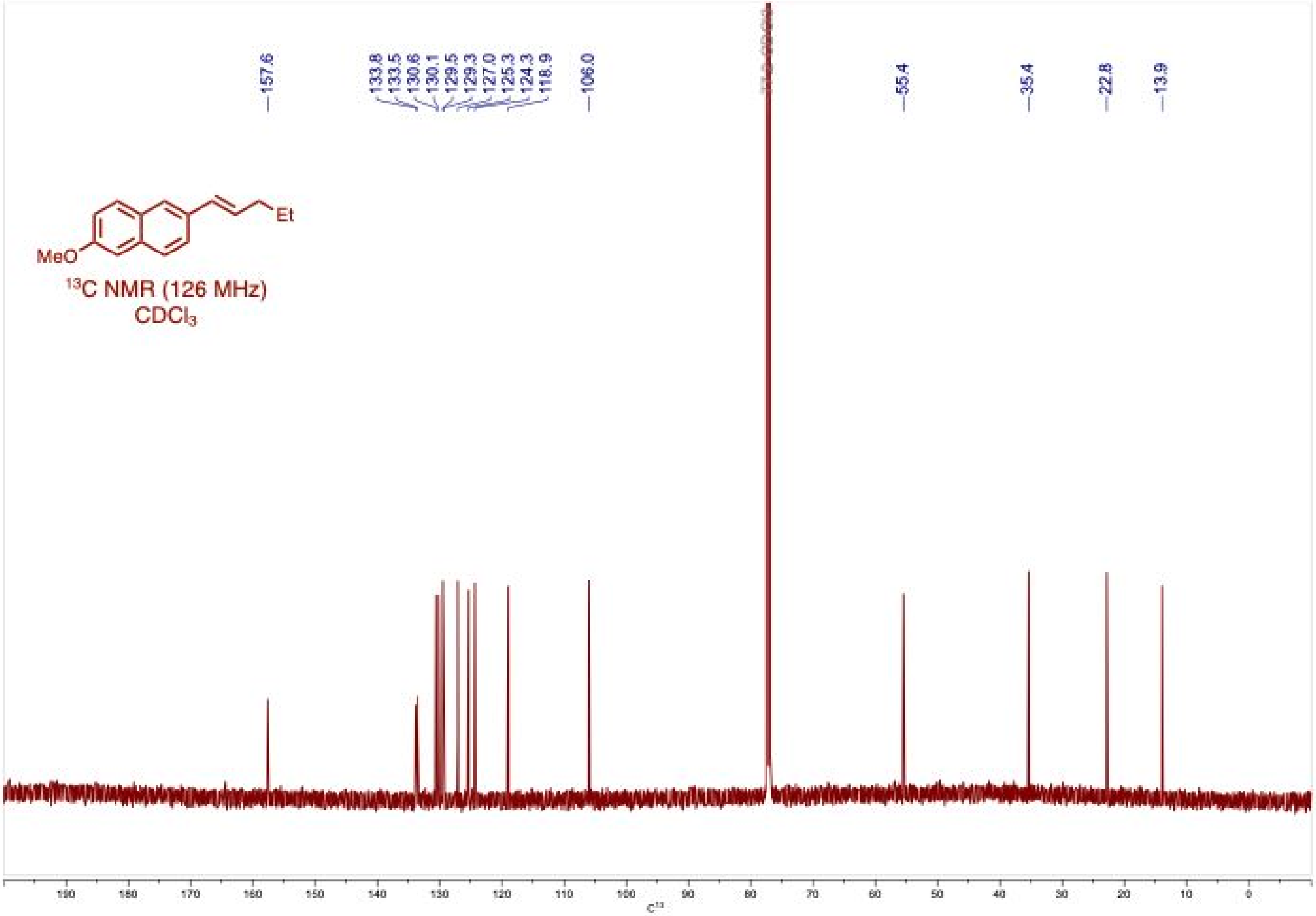

^{1}H (500 MHz, $CDCl_3$) NMR Spectrum of 39

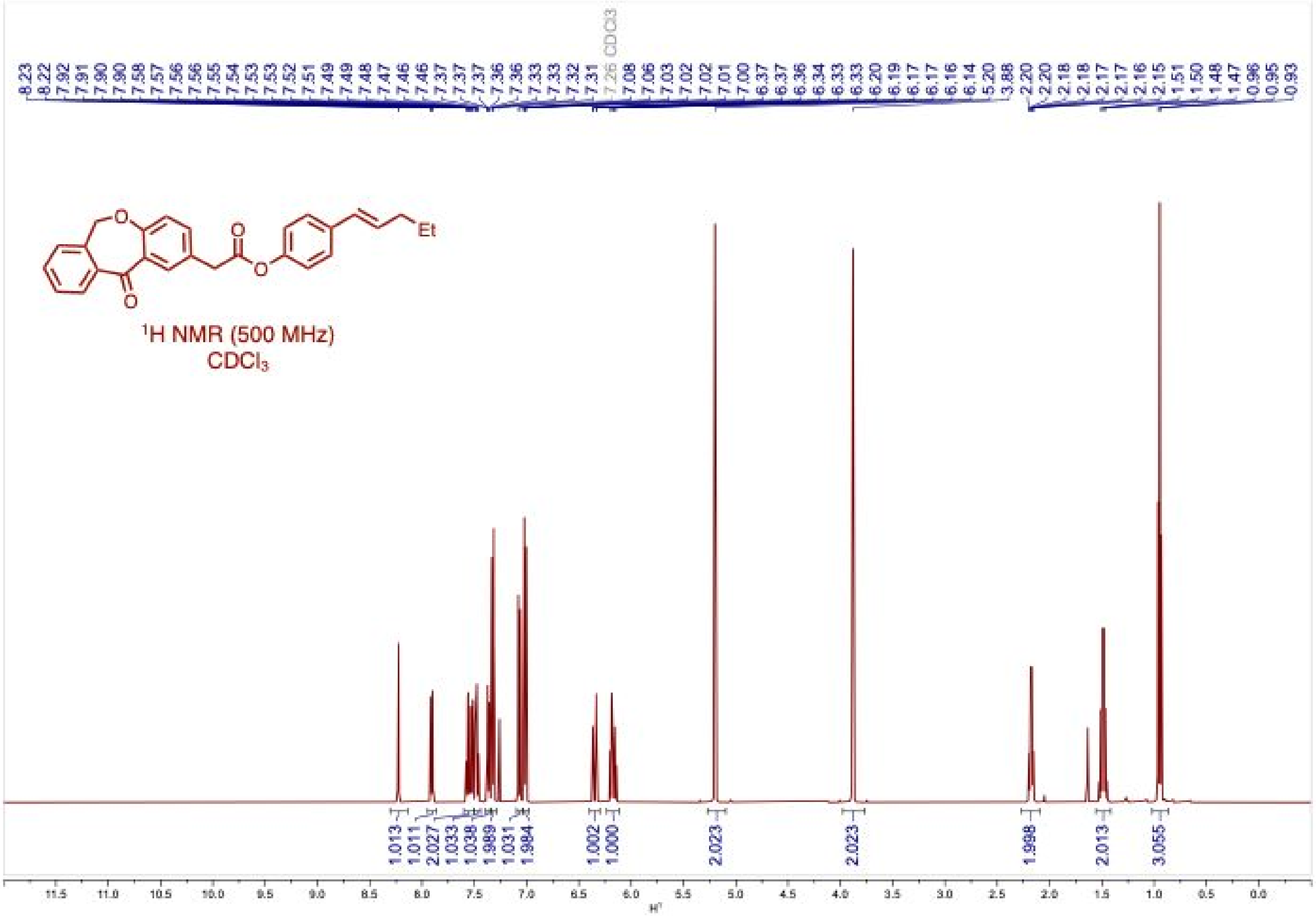

^{13}C (126 MHz, $CDCl_3$) NMR Spectrum of 39

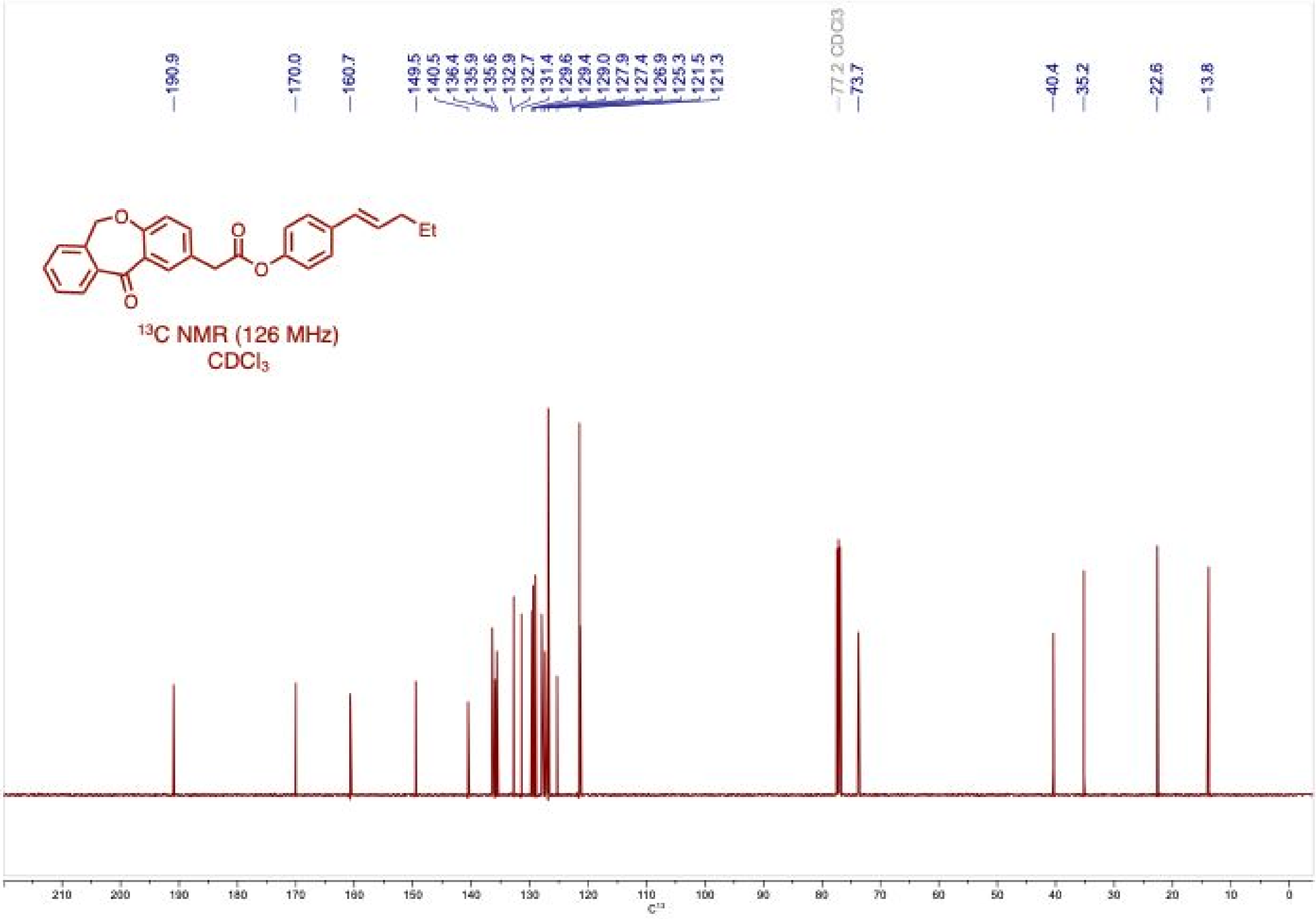

^{1}H (500 MHz, $CDCl_3$) NMR Spectrum of 40

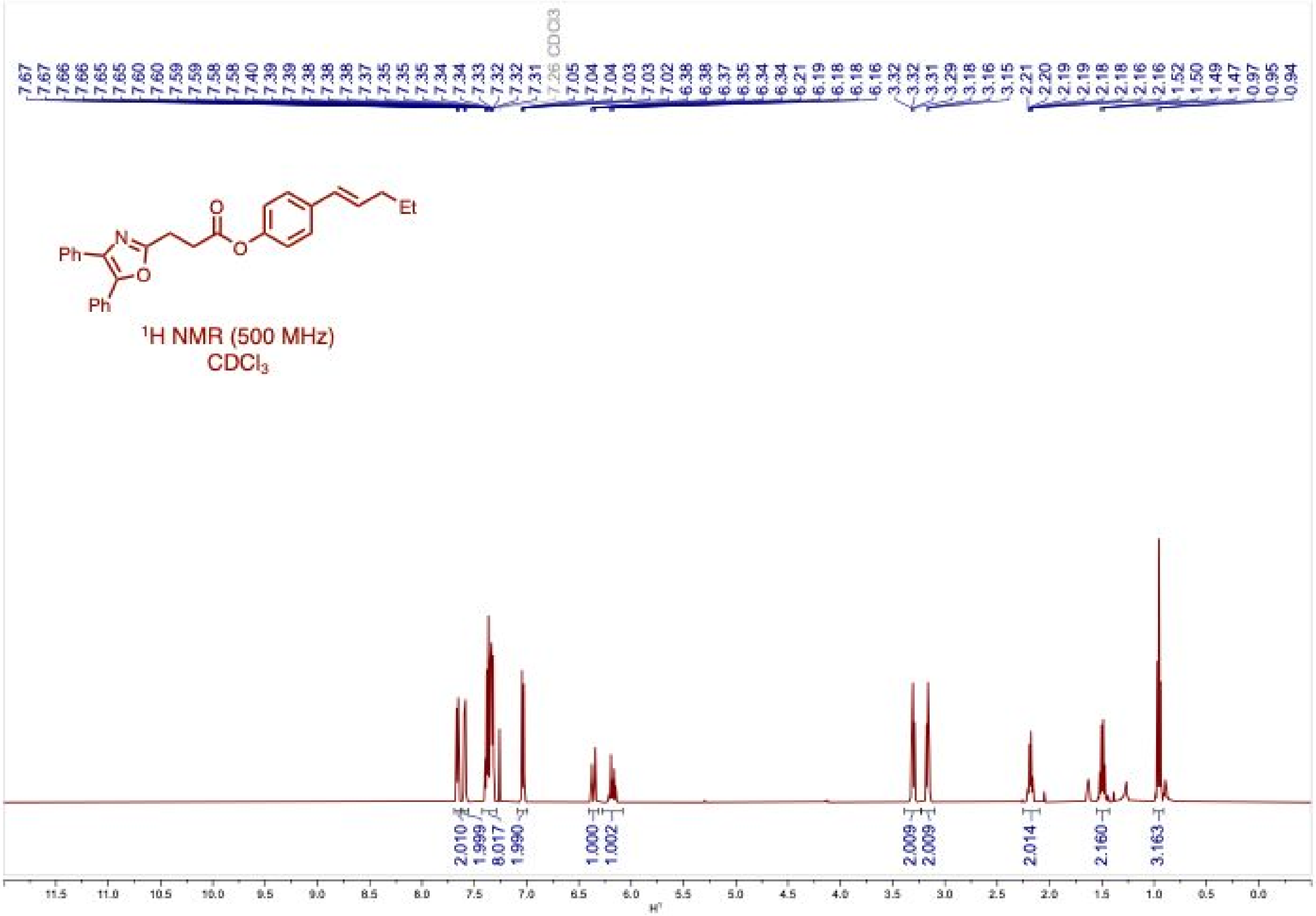

^{13}C (126 MHz, $CDCl_3$) NMR Spectrum of 40

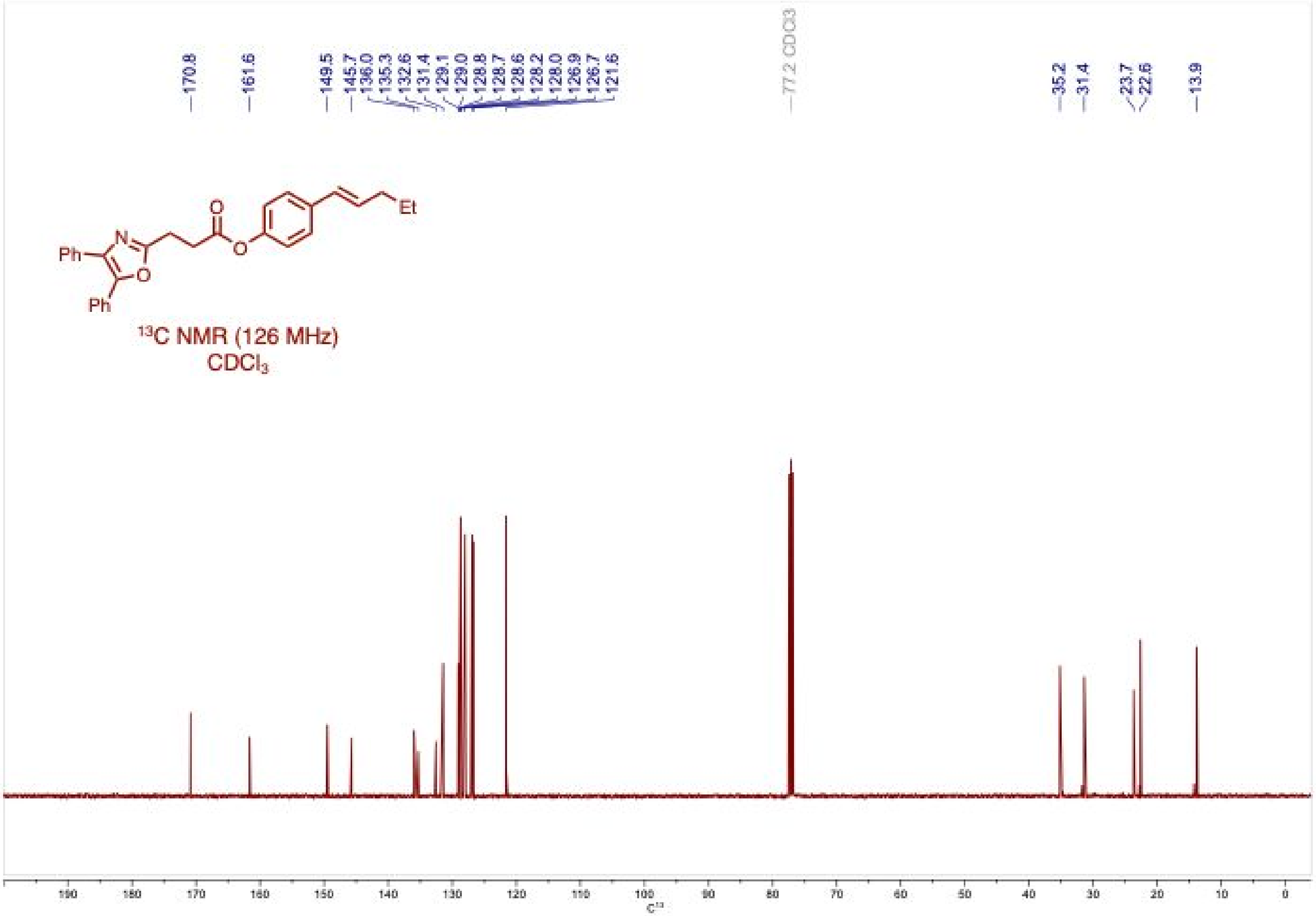

^{1}H (500 MHz, $CDCl_3$) NMR Spectrum of 41

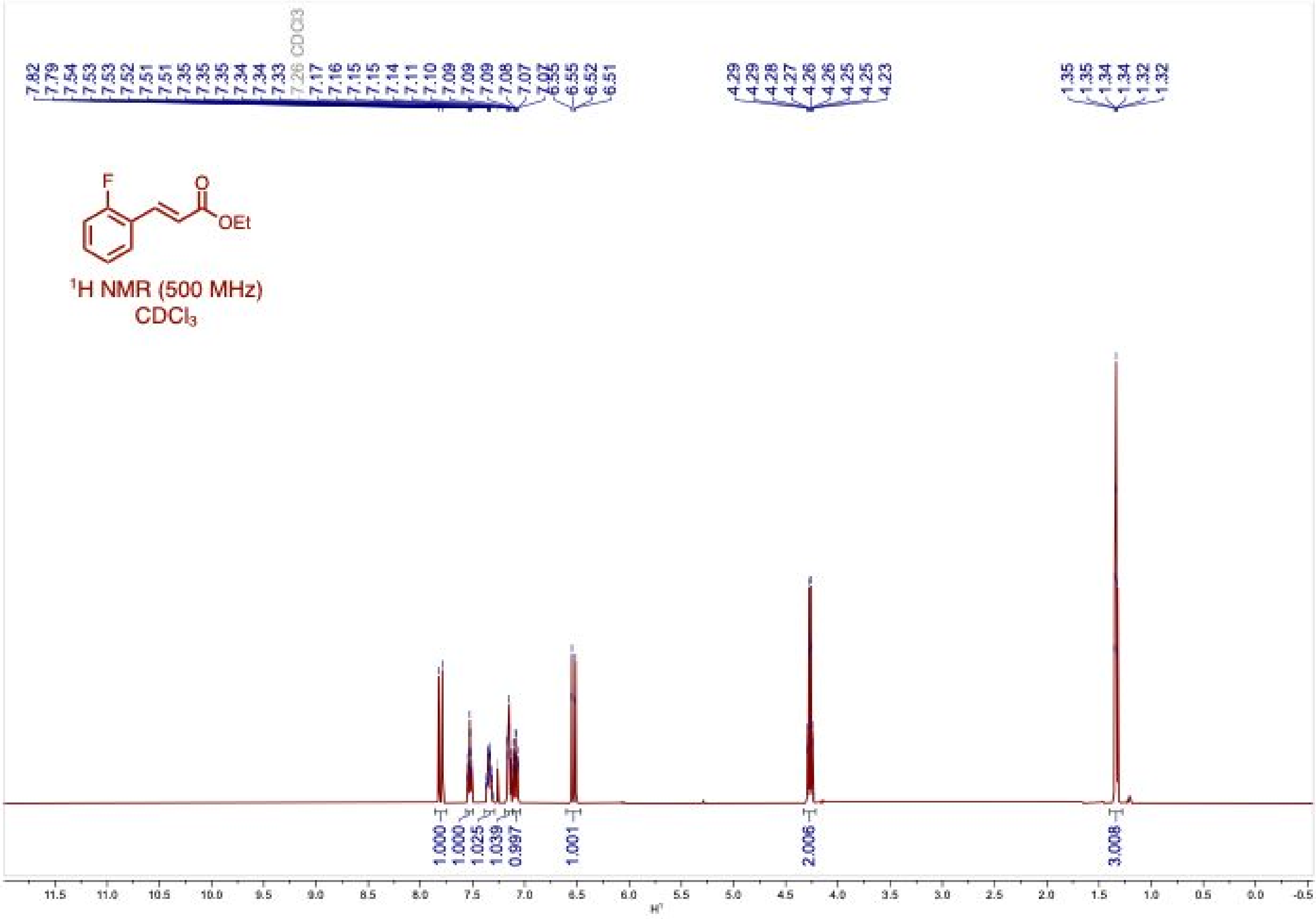

^{13}C (126 MHz, $CDCl_3$) NMR Spectrum of 41

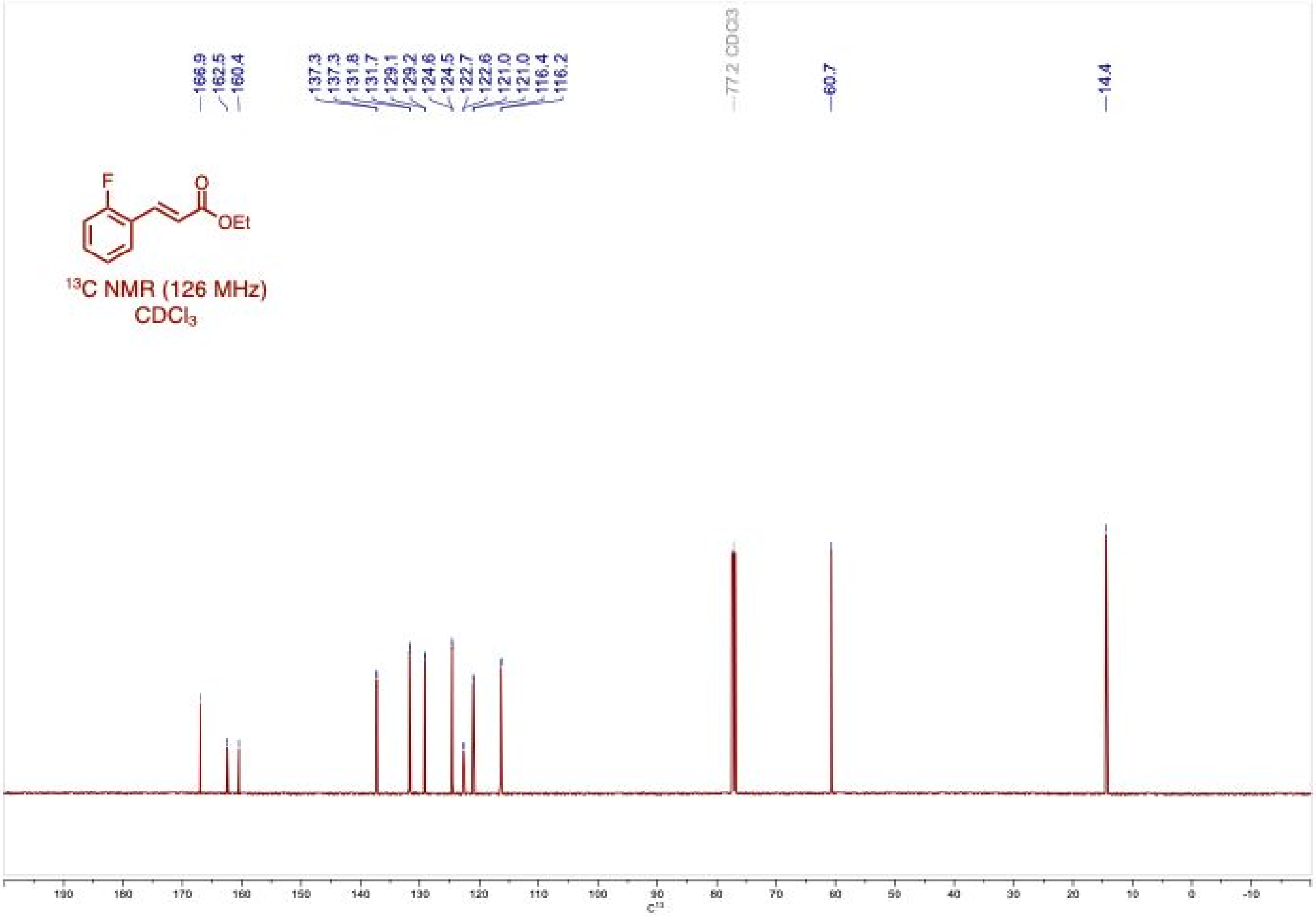

^{19}F (470 MHz, $CDCl_3$) NMR Spectrum of 41

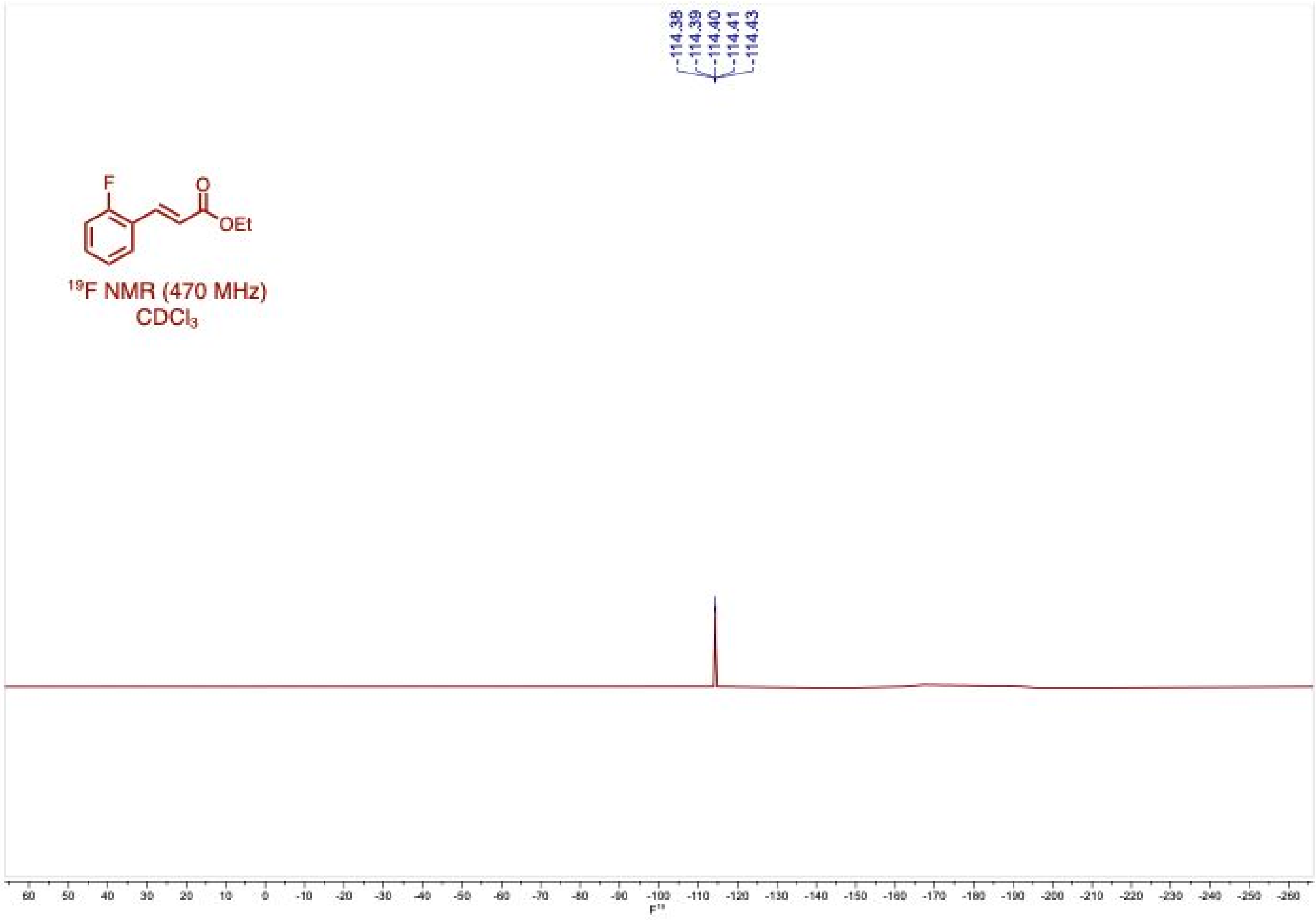

^{1}H (500 MHz, $CDCl_3$) NMR Spectrum of 42

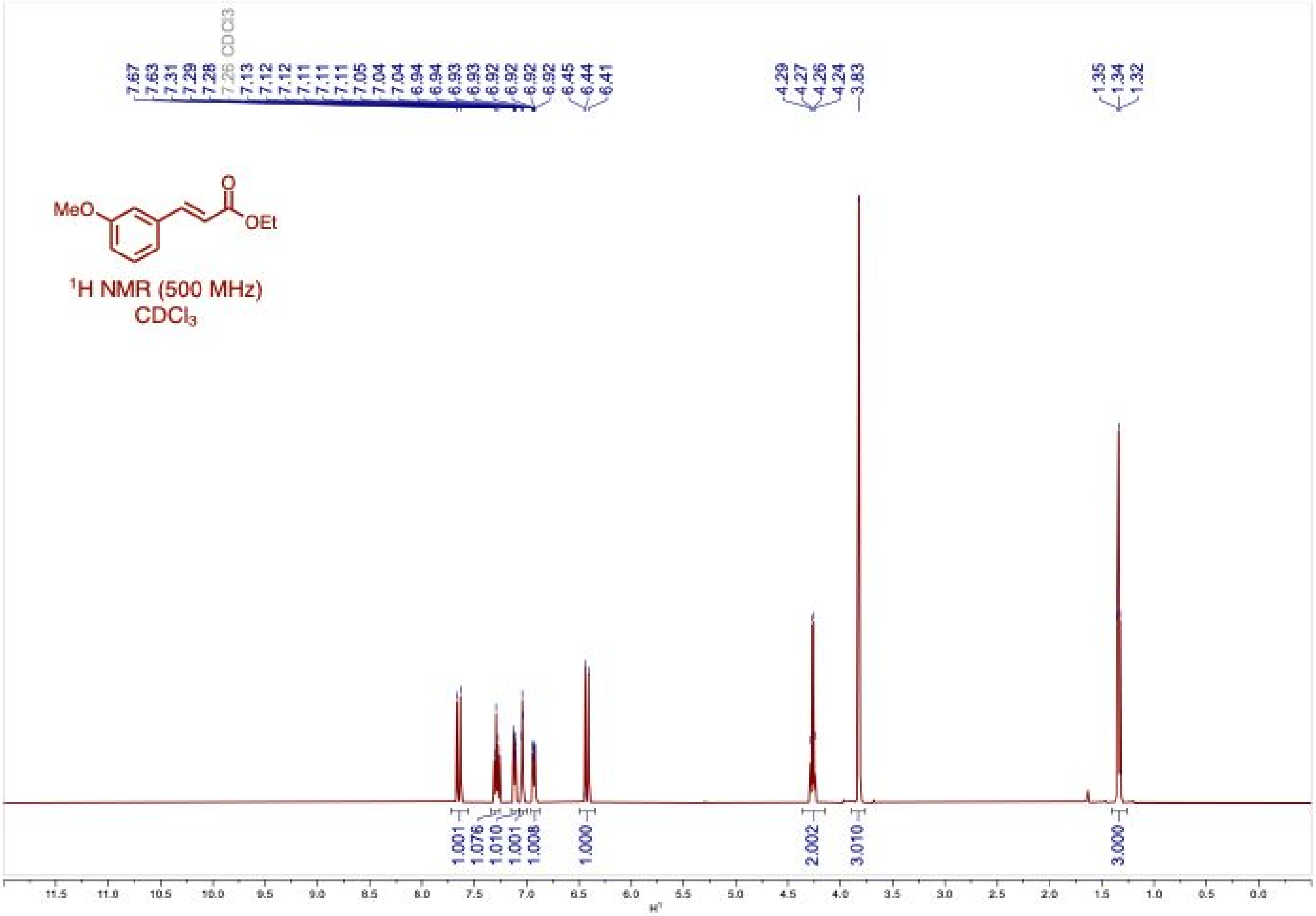

^{13}C (126 MHz, CDCl$_3$) NMR Spectrum of 42

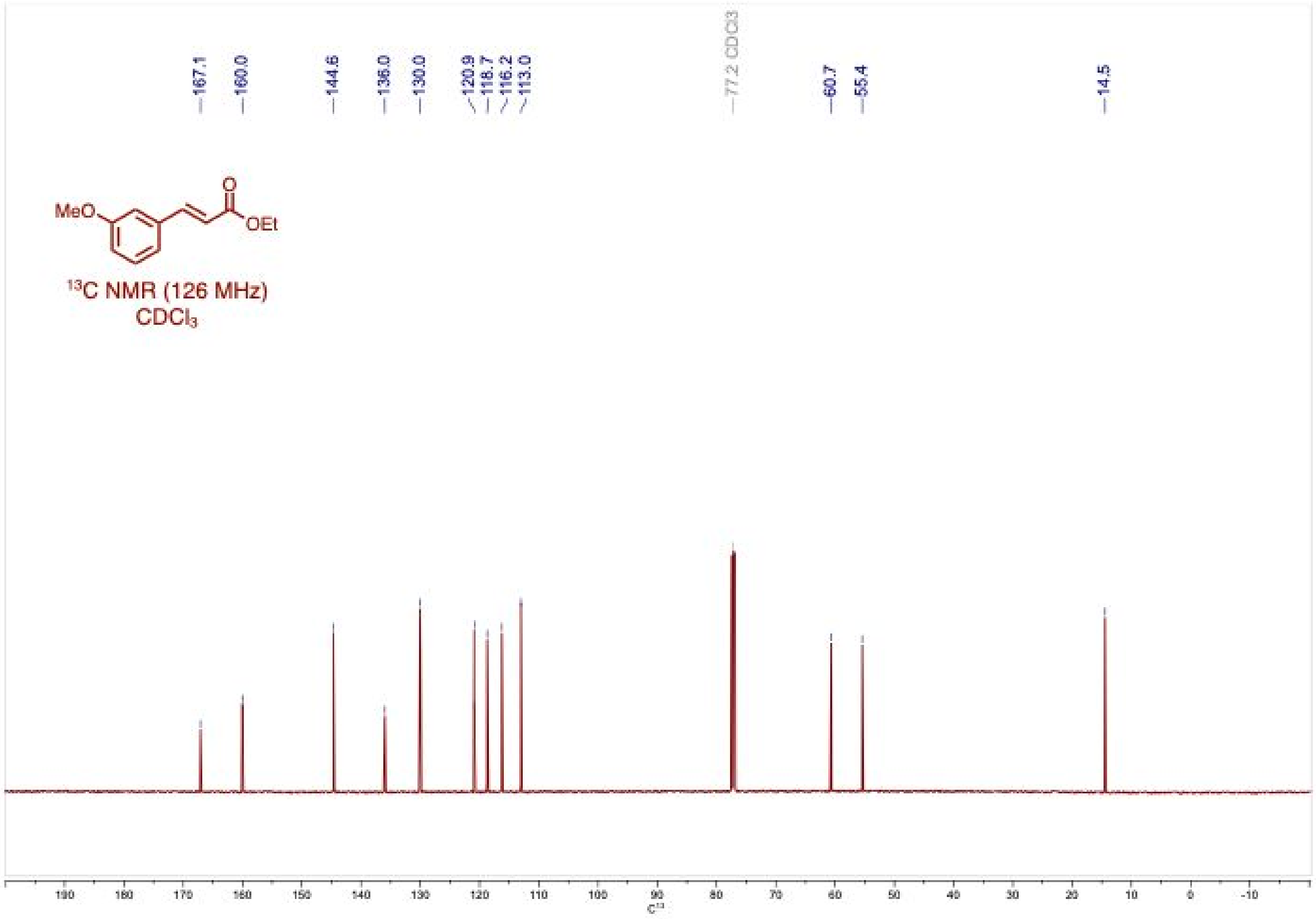

^1H (500 MHz, $CDCl_3$) NMR Spectrum of 43

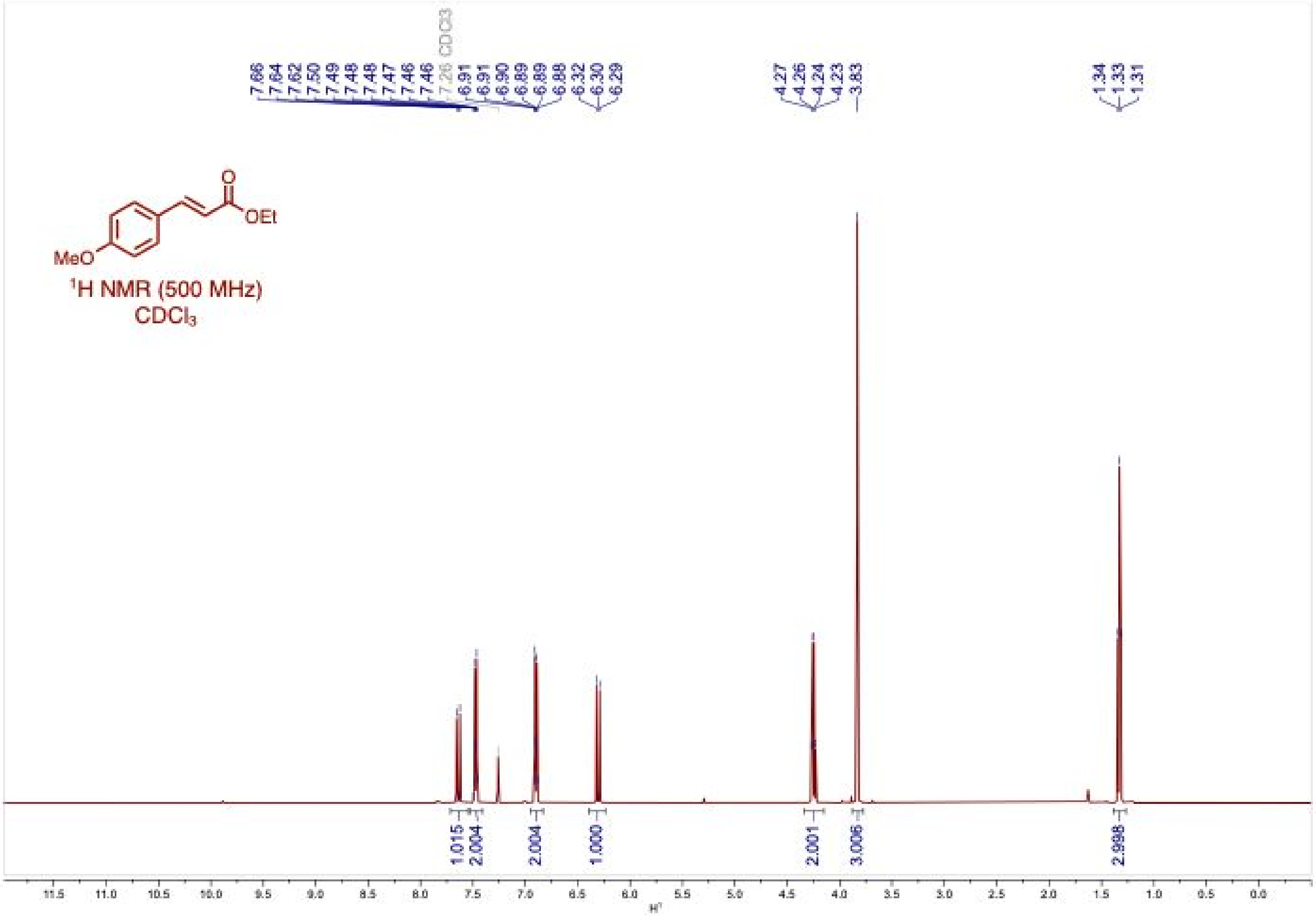

^{13}C (126 MHz, $CDCl_3$) NMR Spectrum of 43

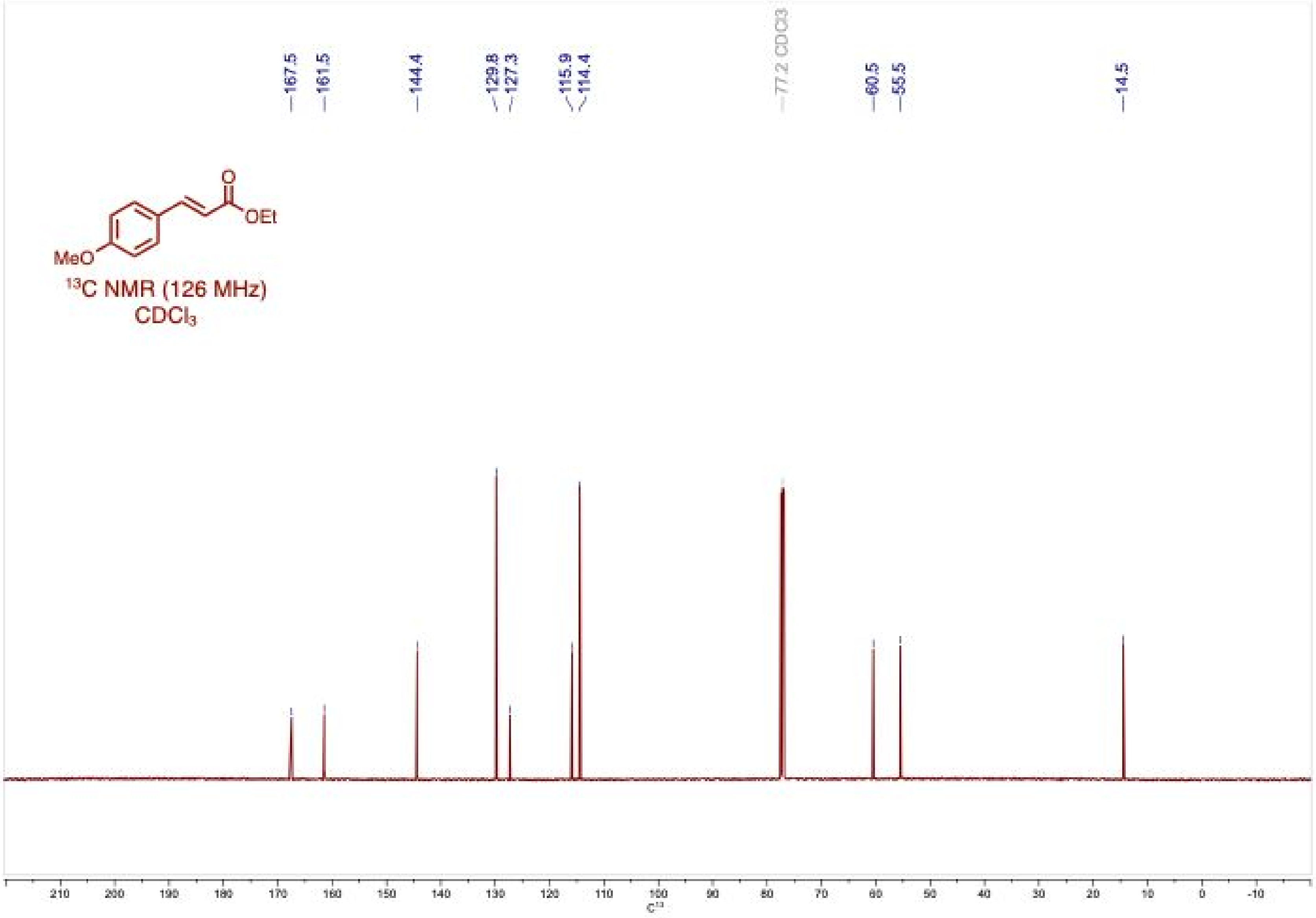

^{1}H (500 MHz, $CDCl_3$) NMR Spectrum of 44

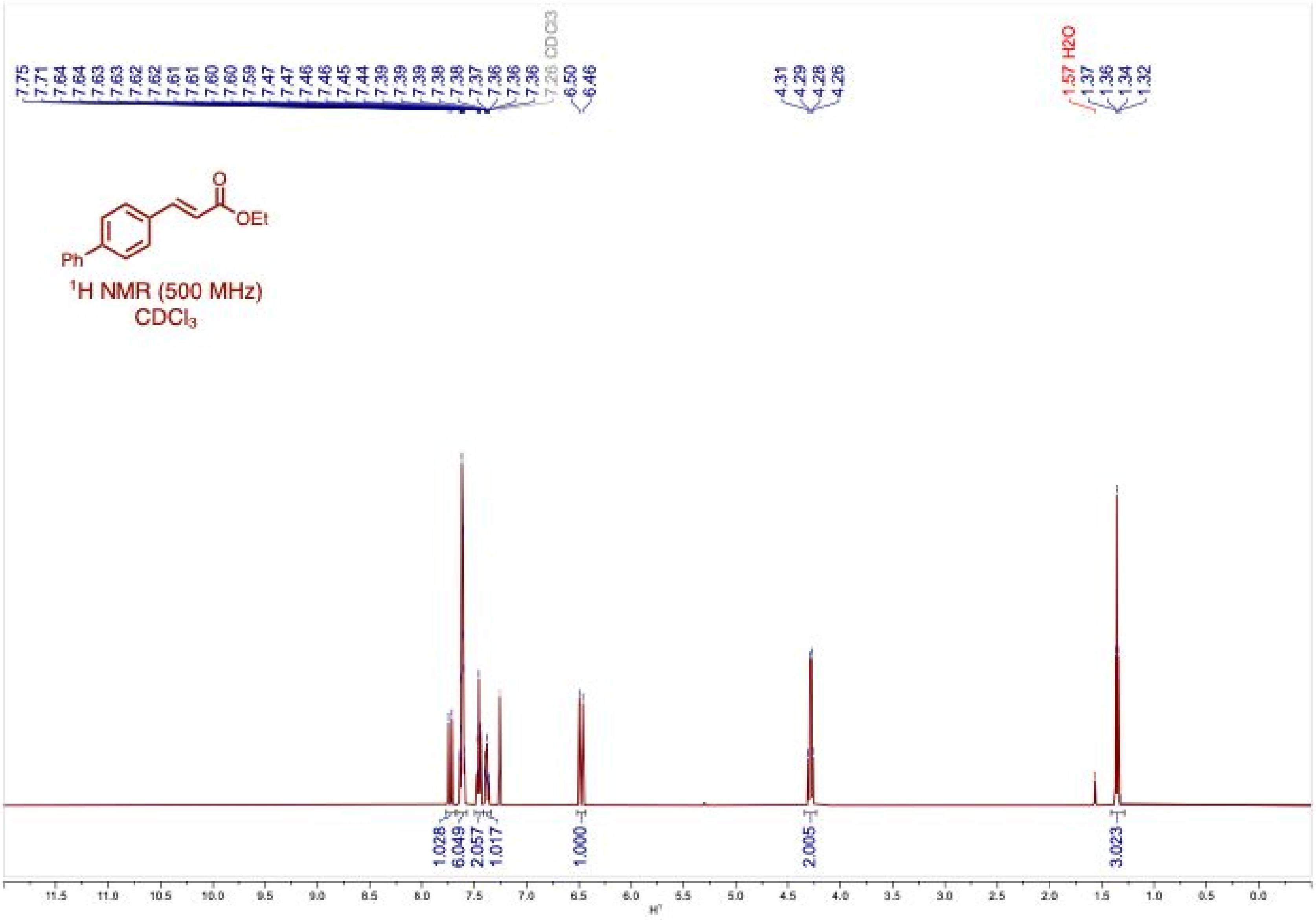

^{13}C (126 MHz, $CDCl_3$) NMR Spectrum of 44

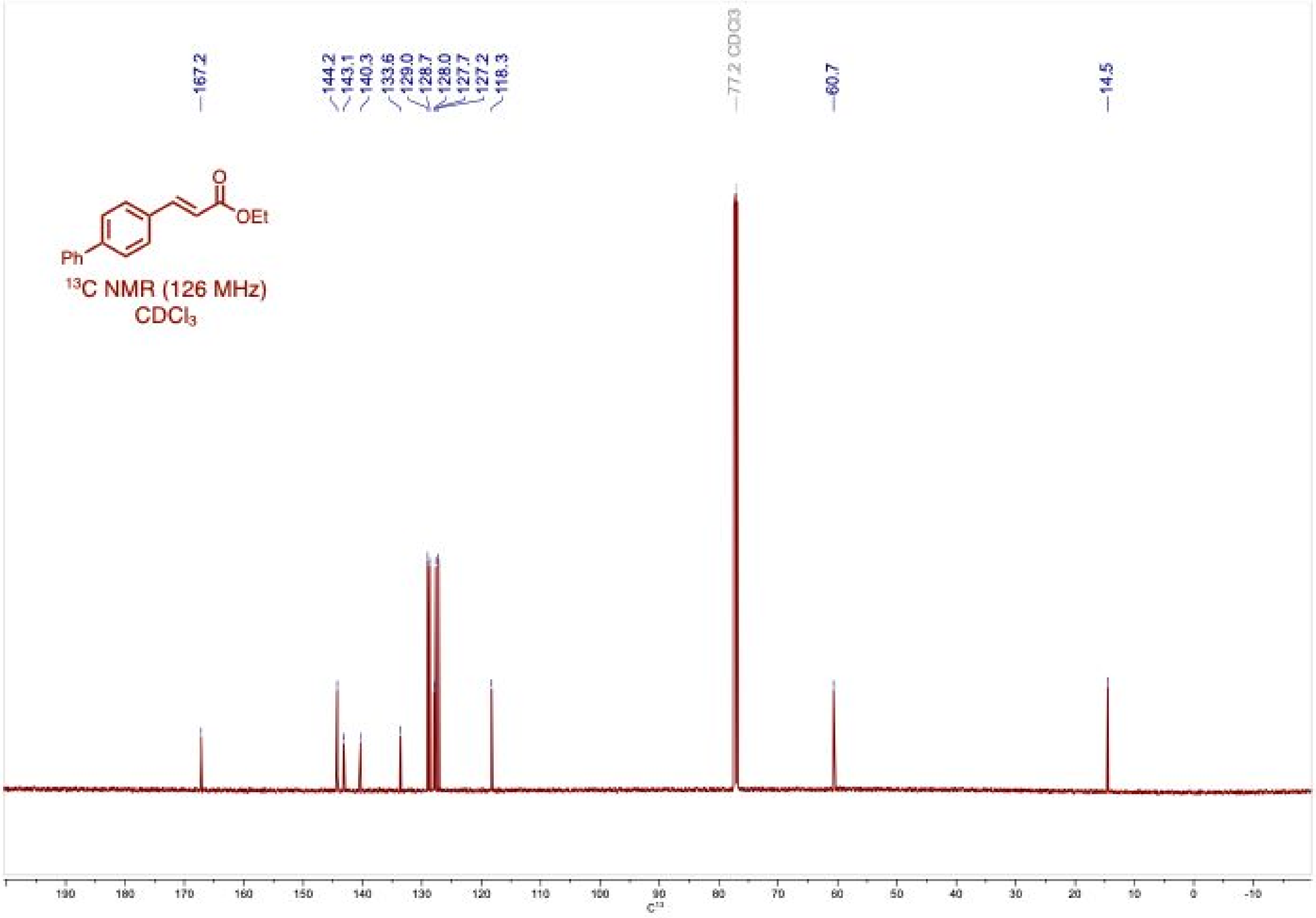

^{1}H (500 MHz, $CDCl_3$) NMR Spectrum of 45

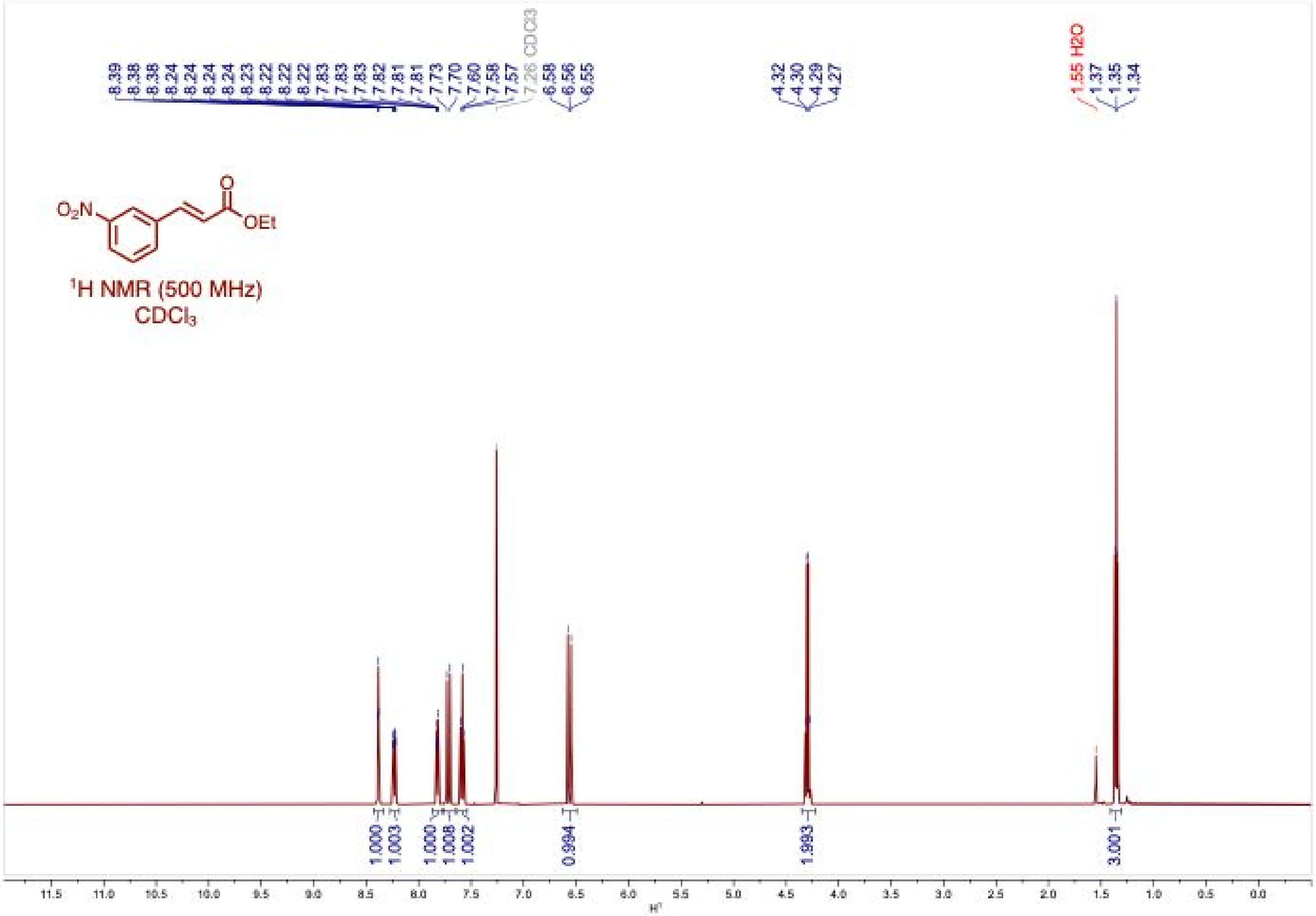

^{13}C (126 MHz, $CDCl_3$) NMR Spectrum of 45

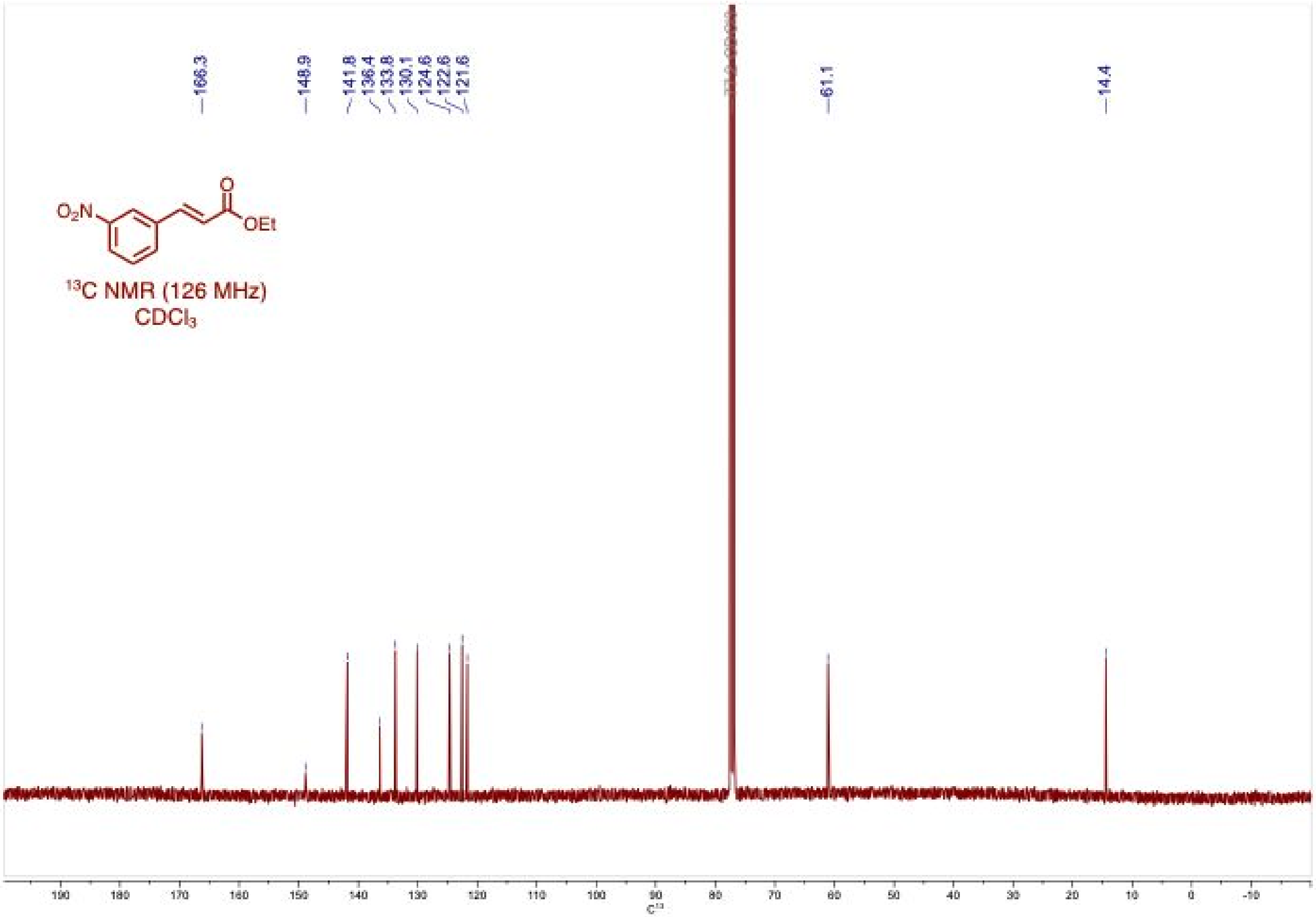

^{1}H (500 MHz, $CDCl_3$) NMR Spectrum of 46

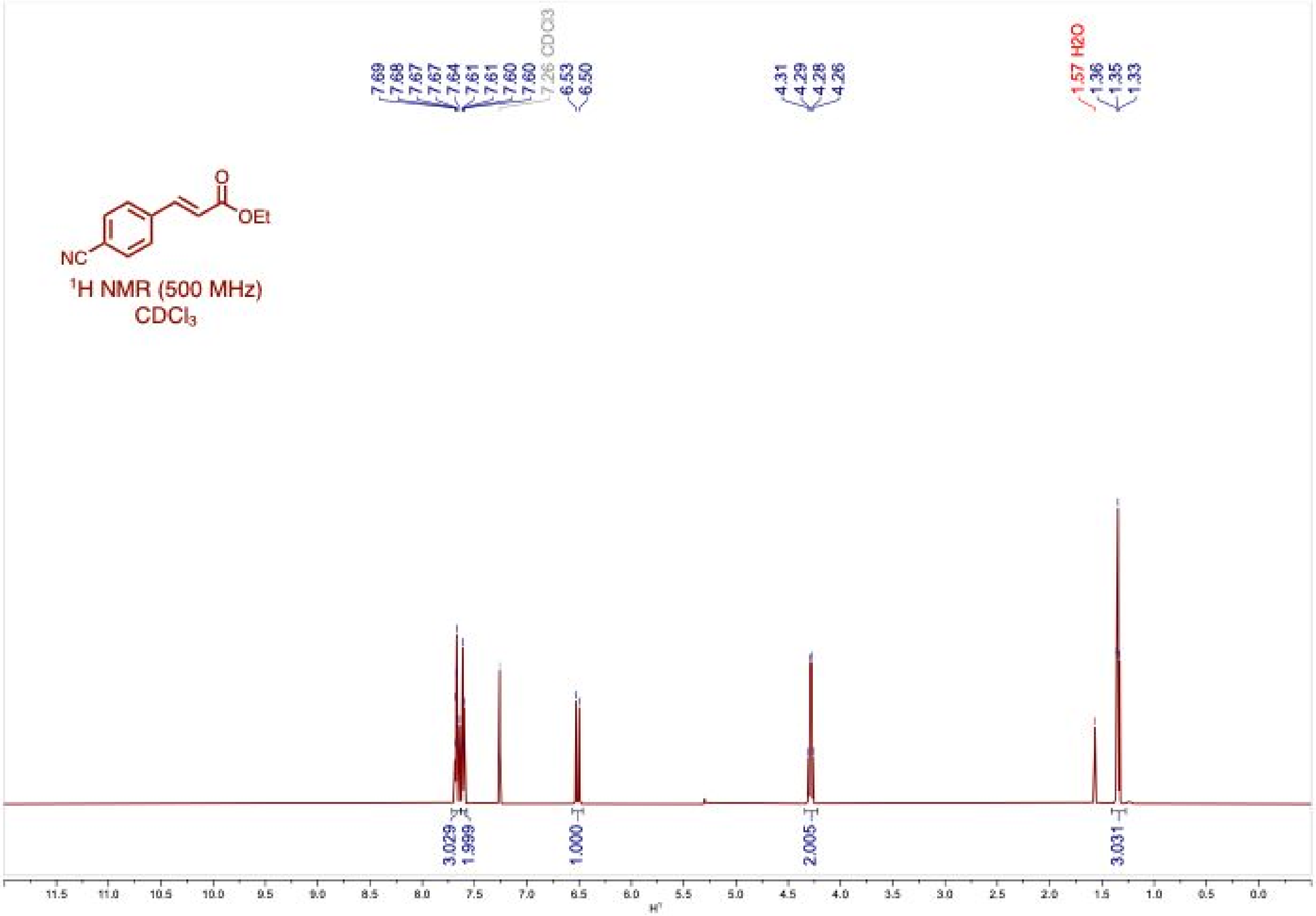

^{13}C (126 MHz, $CDCl_3$) NMR Spectrum of 46

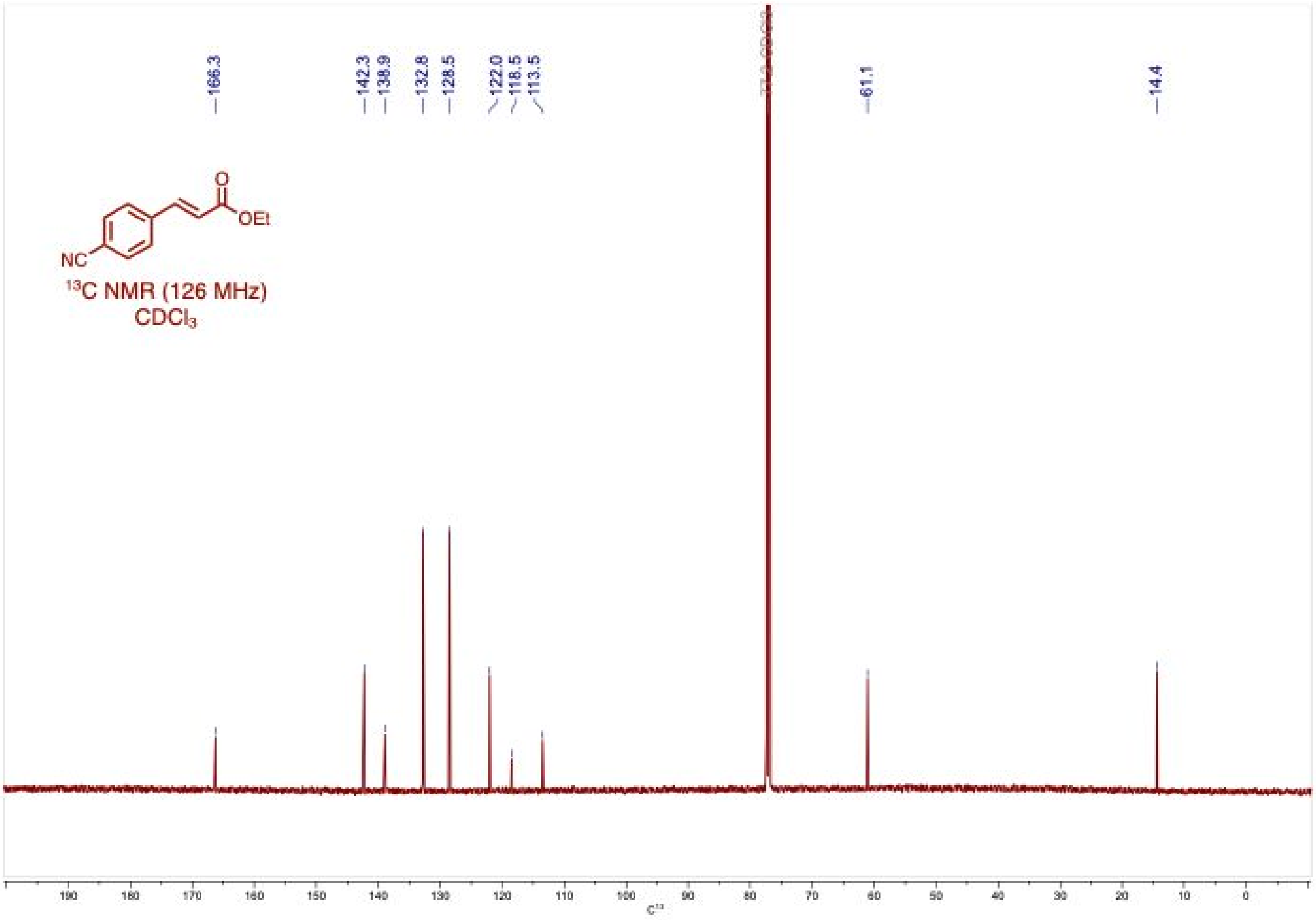

^{1}H (500 MHz, CDCl$_3$) NMR Spectrum of 47

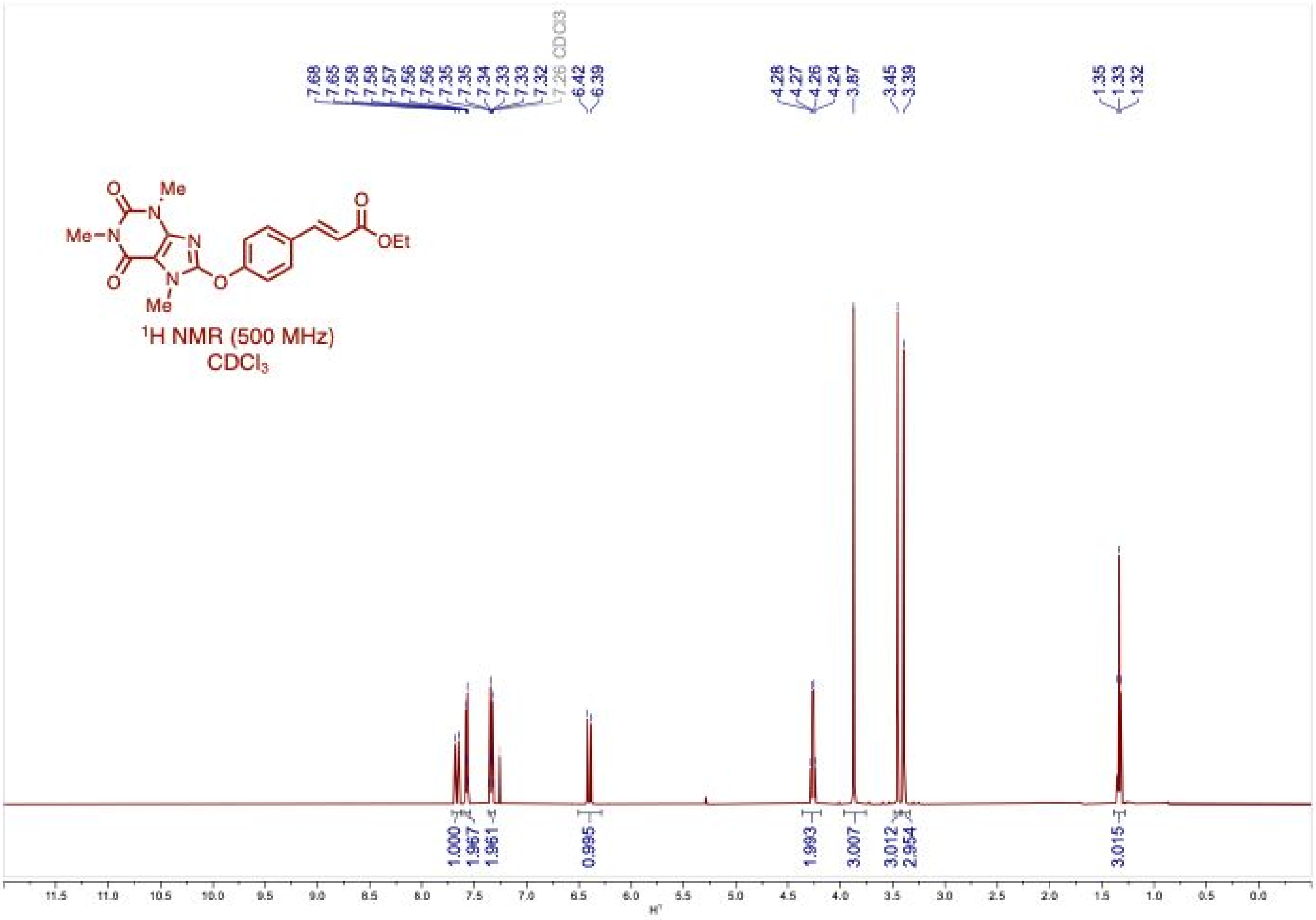

^{13}C (126 MHz, $CDCl_3$) NMR Spectrum of 47

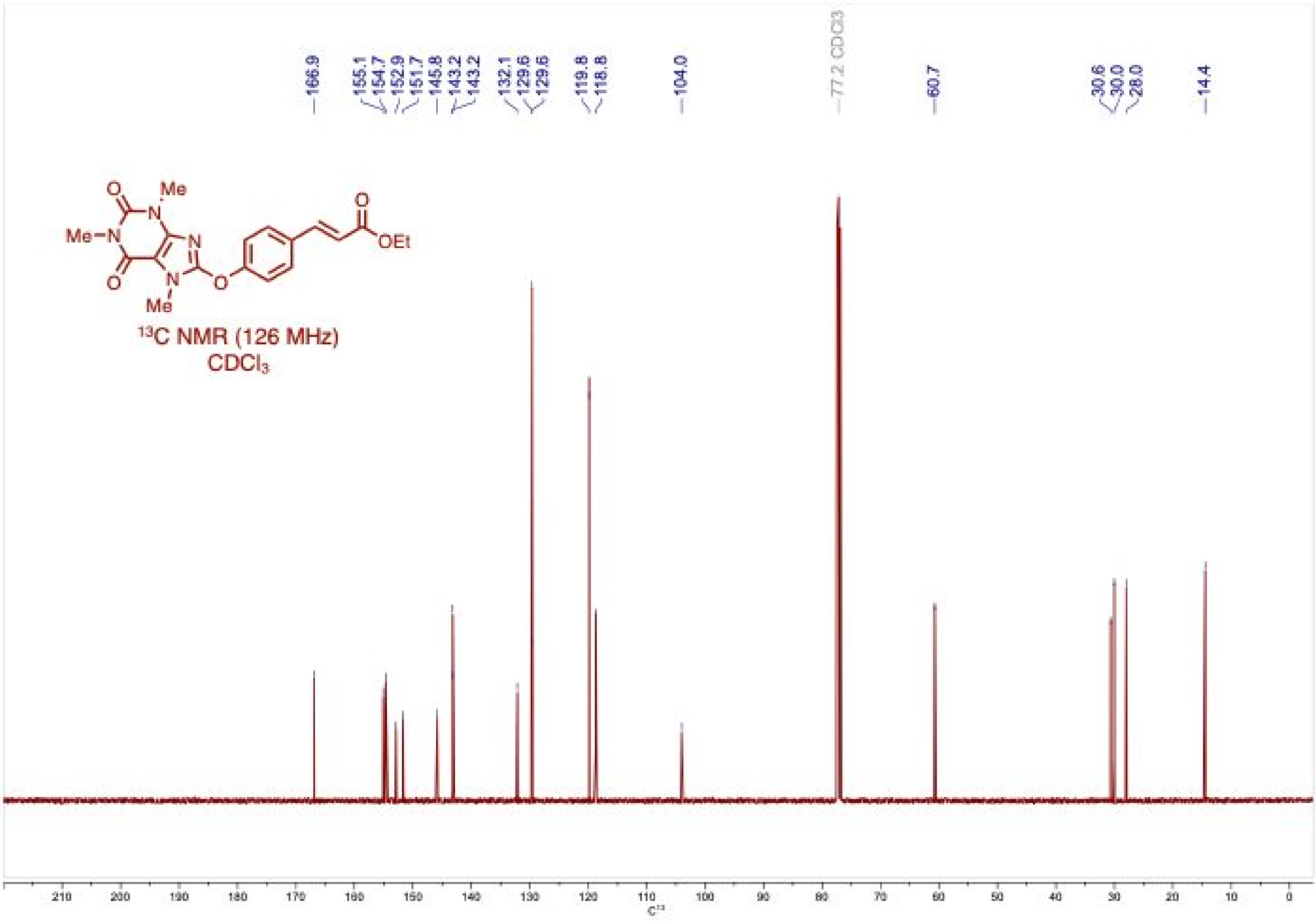

^{1}H (500 MHz, $CDCl_3$) NMR Spectrum of 48

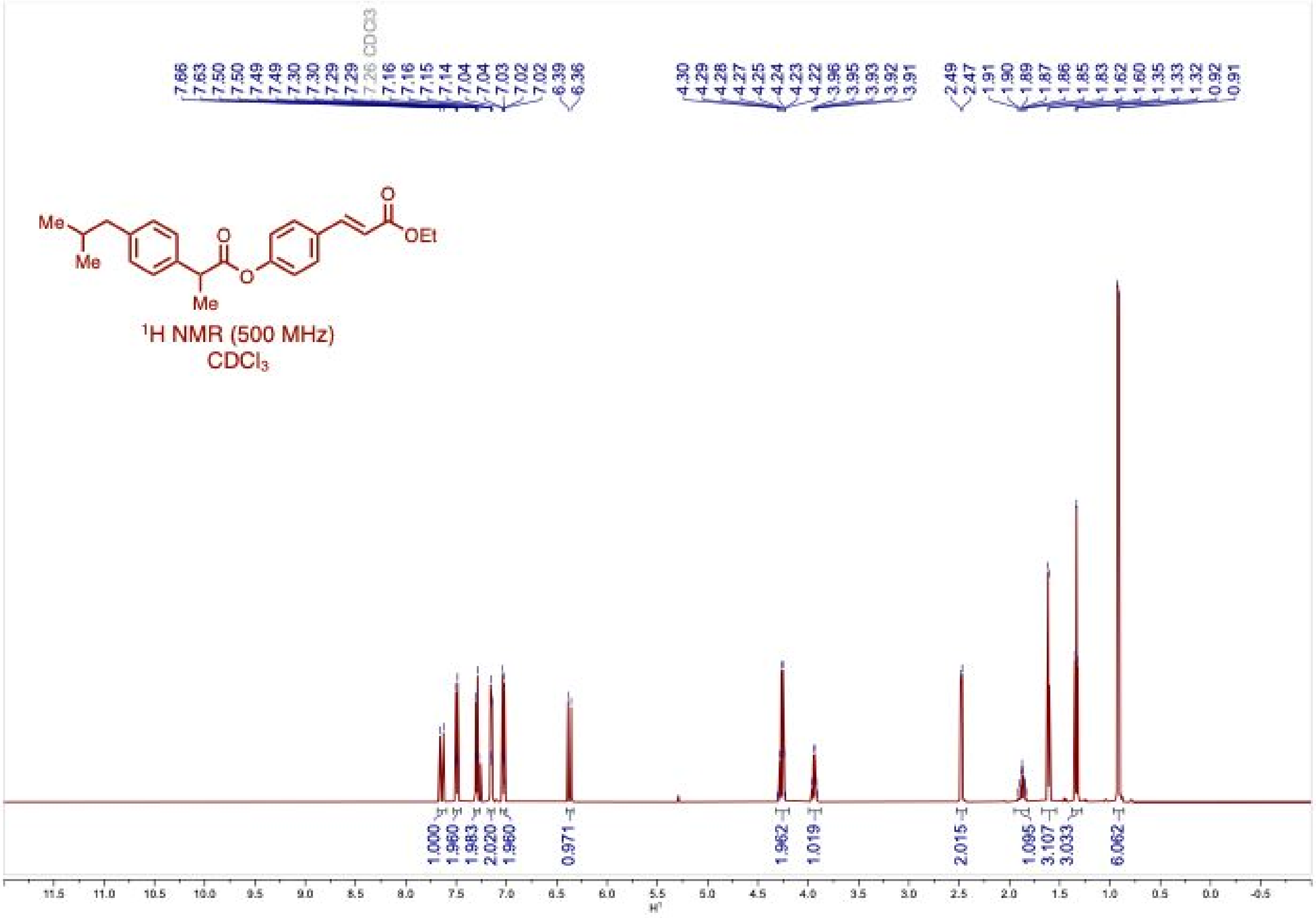

^{13}C (126 MHz, $CDCl_3$) NMR Spectrum of 48

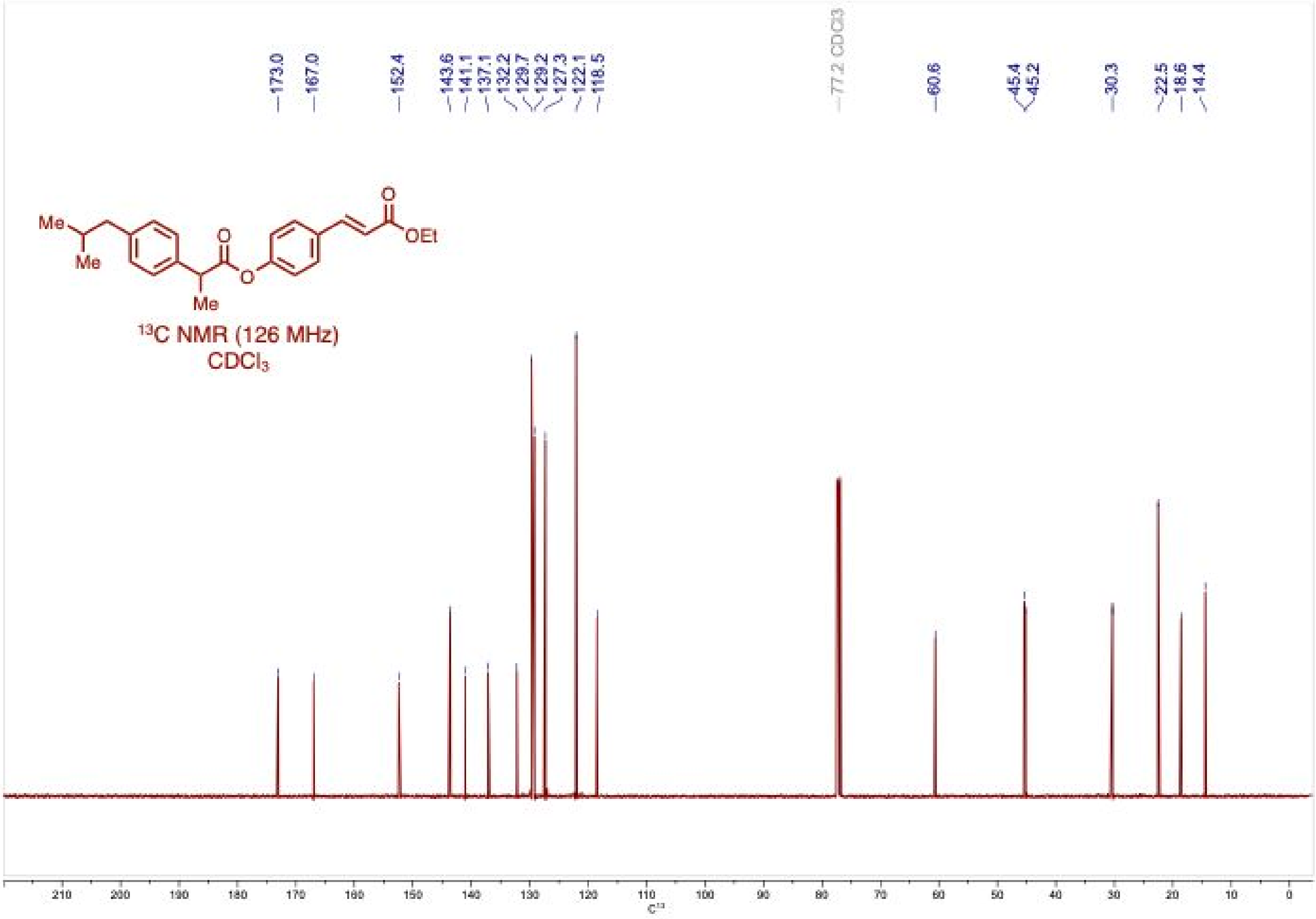

8. REFERENCES

1. Kellner-Rogers, J. S.; Wang, R.; Lambert, T. H. Diazene-Catalyzed Oxidative Alkyl Halide–Olefin Metathesis. *Org. Lett.* **2024**, *26*, 1078–1082
2. Quach, P. K.; Hsu, J. H.; Keresztes, I.; Fors, B. P.; Lambert, T. H. Metal-Free Ring-Opening Metathesis Polymerization with Hydrazonium Initiators. *Angew. Chem. Int. Ed.* **2022**, *134*, e202203344.
3. Zhang, Y.; Ji, P.; Hu, W.; Wei, Y.; Huang, H.; Wang, W. Organocatalytic Transformation of Aldehydes to Thioesters with Visible Light. *Chem. Eur. J.* **2019**, *25*, 8225–8228.
4. Jermaks, J.; Quach, P. K.; Seibel, Z. M.; Pomarole, J.; Lambert, T. H. Ring-Opening Carbonyl–Olefin Metathesis of Norbornenes. *Chem. Sci.* **2020**, *11*, 7884–7895.
5. Siddiqi, Z. R.; Ungarean, C. N.; Bingham, T. W.; Sarlah, D. Development of a Scalable and Sublimation-Free Route to MTAD. *Org. Process Res. Dev.* **2020**, *24*, 2953–2959.
6. Jiang, Y.-S.; Liu, F.; Huang, M.-S.; Luo, X.-L.; Xia, P.-J. Photocatalytic Modular Cyanoalkylamination of Alkenes Involving Two Different Iminyl Radicals. *Org. Lett.* **2022**, *24*, 8019–8024.
7. Lau, S.-H.; Bourne, S. L.; Martin, B.; Schenkel, B.; Penn, G.; Ley, S. V. Synthesis of a Precursor to Sacubitril Using Enabling Technologies. *Org. Lett*. **2015**, *17*, 5436–5439.
8. Yu, J.; Zhou, Y.; Lin, Z.; Tong, R. Regioselective and Stereospecific Copper-Catalyzed Deoxygenation of Epoxides to Alkenes. *Org. Lett*. **2016**, *18*, 4734–4737.
